# Magmatic-hydrothermal fluid evolution of the tin-polymetallic metallogenic systems from the Weilasituo ore district, Northeast China

**DOI:** 10.1038/s41598-024-53579-y

**Published:** 2024-02-06

**Authors:** Xu Gao, Zhenhua Zhou, Karel Breiter, Jingwen Mao, Rolf L. Romer, Nigel J. Cook, François Holtz

**Affiliations:** 1 MNR Key Laboratory of Metallogeny and Mineral Assessment, Institute of Mineral Resources, Chinese Academy of Geological Sciences, Beijing, 100037 China; 2 Institut für Mineralogie, Leibniz Universität Hannover, Callinstr. 3, 30167 Hannover, Germany; 3 Research School of Earth Sciences, Australian National University, 142 Mills Rd, Canberra, ACT 2601 Australia; 4 Institute of Geology of the Czech Academy of Sciences, Rozvojová 269, 16500 Praha 6, Czech Republic; 5 GFZ German Research Centre for Geosciences, Telegrafenberg, 14473 Potsdam, Germany; 6 School of Chemical Engineering, The University of Adelaide, Adelaide, SA 5005 Australia

**Keywords:** In situ LA-ICP-MS trace element analysis, H-O isotopes, Fluid mixing, Physico-chemical conditions, Sn-polymetallic ore systems, Planetary science, Solid Earth sciences

## Abstract

The large Weilasituo Sn-polymetallic deposit is a recent exploration discovery in the southern Great Xing’an Range, northeast China. The ore cluster area shows horizontal mineralization zoning, from the inner granite body outward, consisting of high-*T* Sn–W–Li mineralization, middle-*T* Cu–Zn mineralization and peripheral low-*T* Pb–Zn–Ag mineralization. However, the intrinsic genetic relationship between Sn-W-Li mineralization and peripheral vein-type Pb–Zn–Ag–Cu mineralization, the formation mechanism and the deep geological background are still insufficiently understood. Here, we use fluid inclusions, trace elements concentrations in quartz and sphalerite, and H–O isotope studies to determine the genetic mechanism and establish a metallogenic model. Fluid inclusion microthermometry and Laser Raman spectroscopic analysis results demonstrates that the aqueous ore-forming fluids evolved from low-medium salinity, medium–high temperature to low salinity, low-medium temperature fluids. Laser Raman spectroscopic analysis shows that CH_4_ is ubiquitous in fluid inclusions of all ore stages. Early ore fluids have δ^18^O_H2O (v–SMOW)_ values from + 5.5 to + 6.2‰ and δD values of approximately − 67‰, concordant with a magmatic origin. However, the late ore fluids shifted toward lower δ^18^O_H2O (v–SMOW)_ (as low as 0.3‰) and δD values (~ − 136‰), suggesting mixing between external fluids derived from the wall rocks and a contribution from meteoric water. Ti-in-quartz thermometry indicates a magmatic crystallization temperature of around 700 °C at a pressure of 1.5 kbar for the magmatic ore stage. Cathodoluminescence (CL) imaging and trace element analysis of quartz from a hydrothermal vug highlight at least three growth episodes that relate to different fluid pulses; each episode begins with CL-bright, Al-Li-rich quartz, and ends with CL-dark quartz with low Al and Li contents. Quartz from Episode 1 formed from early Sn-(Zn)-rich fluids which were likely derived from the quartz porphyry. Quartz from episodes 2 and 3 formed from Zn-(Sn)-Cu-rich fluid. The early magmatic fluid is characterized by low *f*S_2_. The SO_2_ produced by magma degassing reacted with heated water to form SO_4_^2−^, causing the shift from low *f*S_2_ to high *f*S_2_. The SO_4_^2−^ generated was converted to S^2–^ by mixing with CH_4_-rich, Fe and Zn-bearing external fluid which led to late-stage alteration and dissolution of micas in vein walls, thus promoting crystallization of pyrrhotite, Fe-rich sphalerite and chalcopyrite and inhibiting the precipitation of anhydrite. This study shows that ore formation encompassed multiple episodes involving steadily evolved fluids, and that the addition of external fluids plays an important role in the formation of the later Cu–Zn and Ag–Pb–Zn mineralization in the Weilasituo ore district.

## Introduction

In recent years, numerous examples of tin-polymetallic mineralization have been discovered in the southern Great Xing’an Range (SGXR), making the SGXR the most important Sn belt in North China. Among these newly explored deposits, the Weilasituo Sn-polymetallic deposit is the best suited for detailed investigation because of its large reserves and its large-scale metal zonation. The Weilasituo deposit was discovered in 2013 and contains 11.2 Mt of ore at grades of 0.8% Sn, 0.42% WO_3_, 0.12% Mo, and 2.59% Zn^1^. Other nearby deposits include the Weilasituo Cu–Zn deposit (~ 2 km NW) and the Bairendaba Ag–Pb–Zn deposit (~ 6 km NW). These three deposits have been studied previously, focusing on the geology of the deposit^2^, timing of ore formation^1–4^, character of ore-forming fluids^5–7^, and ore source and precipitation mechanisms^8–12^. However, the genetic relationship between the Sn-polymetallic deposit and the other two deposits in the district, if any exists, is poorly understood, thus hampering development of a holistic exploration model. If all mineralization types in the district are products of a single ore system, the observed lateral zoning of mineralization across the district may reflect migration and cooling of ore-forming fluids derived from a single magmatic-hydrothermal system. Alternatively, if ore formation was a multi-episode process, fluids and possibly also metals, may be derived from different sources.

For polymetallic deposits related to granitic intrusions, the magmatic-hydrothermal transition is a key process for the formation of hydrothermal ore deposits^13,14^. Consequently, constraining the physical and chemical evolution of ore-forming fluids during this process is important. Rapid advances in microanalytical capability in recent years have led to an improved understanding of the evolution of ore-forming fluids and ore precipitation mechanisms^15–17^. Given that quartz is mechanically and chemically resistant, quartz geochemistry is a robust tool to evaluate the fractionation mechanisms and crystallization processes across the magmatic-hydrothermal transition^18–23^. The Ge/Ti and Al/Ti ratios of quartz can be used as an index of igneous differentiation^20,24,25^ and the Ti content in quartz can indicate temperature, pressure, and TiO_2_ activity in magma^26,27^. In addition, experimental and microanalytical studies show that trace element contents and their ratios in sphalerite can effectively reflect the physical and chemical conditions of the mineralization process^28–30^.

In this contribution, we present new fluid inclusion, isotope geochemistry to ascertain the source and evolution of ore-forming fluids. The abundances of trace elements in sphalerite and quartz in different stages are determined to clarify the distribution and behavior of ore-forming elements during the magmatic-hydrothermal transitions and to trace ore-forming processes. These results enable us to establish a genetic model for the Sn-polymetallic mineralization.

### Regional geological background and geology of the Weilasituo ore district

**Figure 1:**
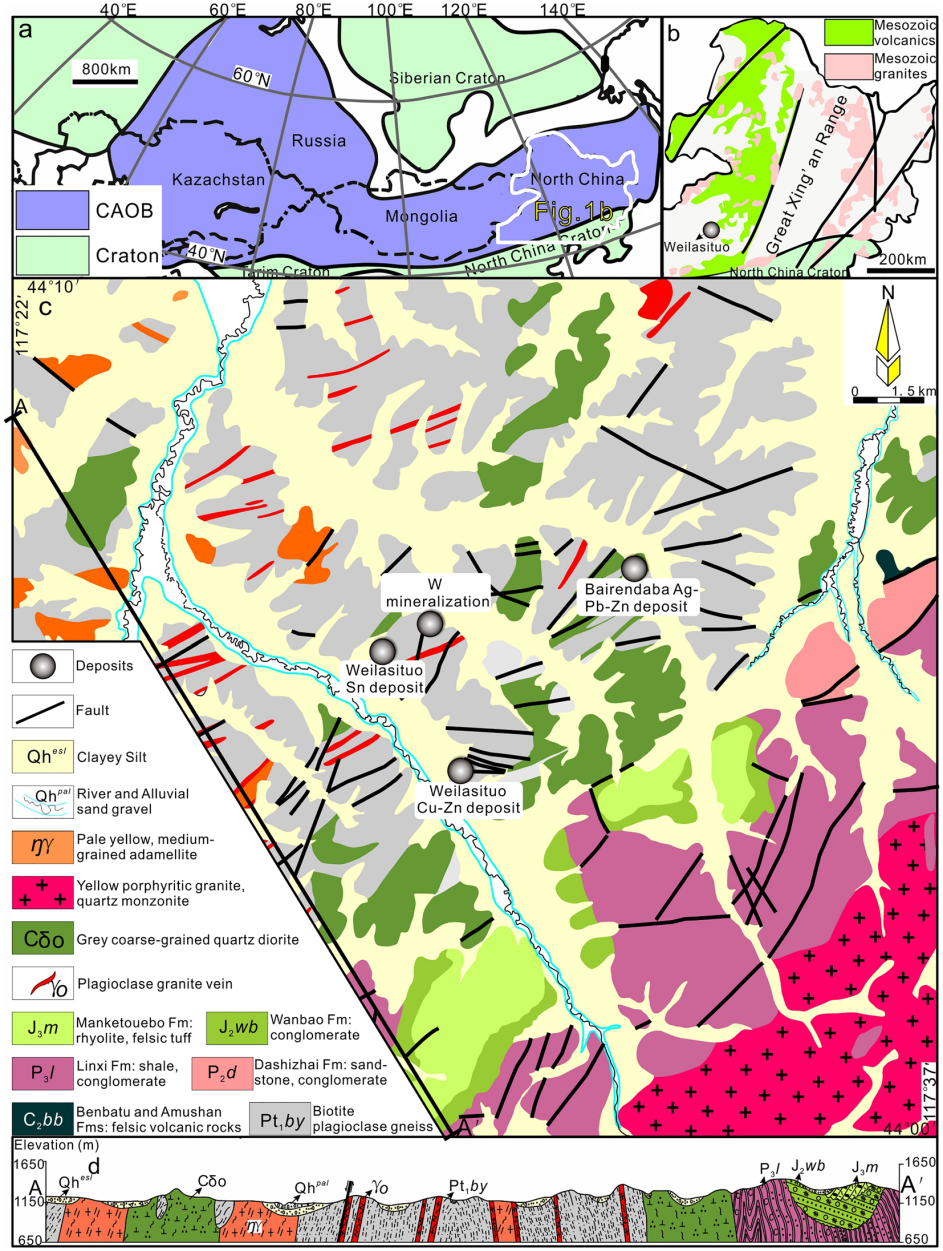
Geological maps. (**a**, **b**) Location of the Great Xing'an Range within the Central Asian Orogenic Belt^37,75,78^ (CAOB). (**c**) Simplified geological map of the Weilasituo and Bairendaba districts. (**d**) Representative vertical cross-section showing the lithology and the contact relations of the various units.

The SGXR is located in the southeastern part of Inner Mongolia, China, which belongs to the eastern part of the Central Asian Orogenic Belt (CAOB) (Fig. 1a, b). Amphibole-plagioclase and biotite-plagioclase gneisses of the Late Paleozoic Xilin Gol Complex^31^ (397–294 Ma) are overlain by (Carboniferous)-Permian to Jurassic sedimentary units (Fig. 1c, d). The latter are dominated by sandstones, siltstones, and carbonaceous mudstones deposited on a shallow shelf (Fig. 1d). There are intercalations of Upper Carboniferous and Jurassic felsic volcanic rocks^32,33^ and Permian andesite, as well as marine, typically bioclastic limestone (Fig. 1c).

**Figure 2:**
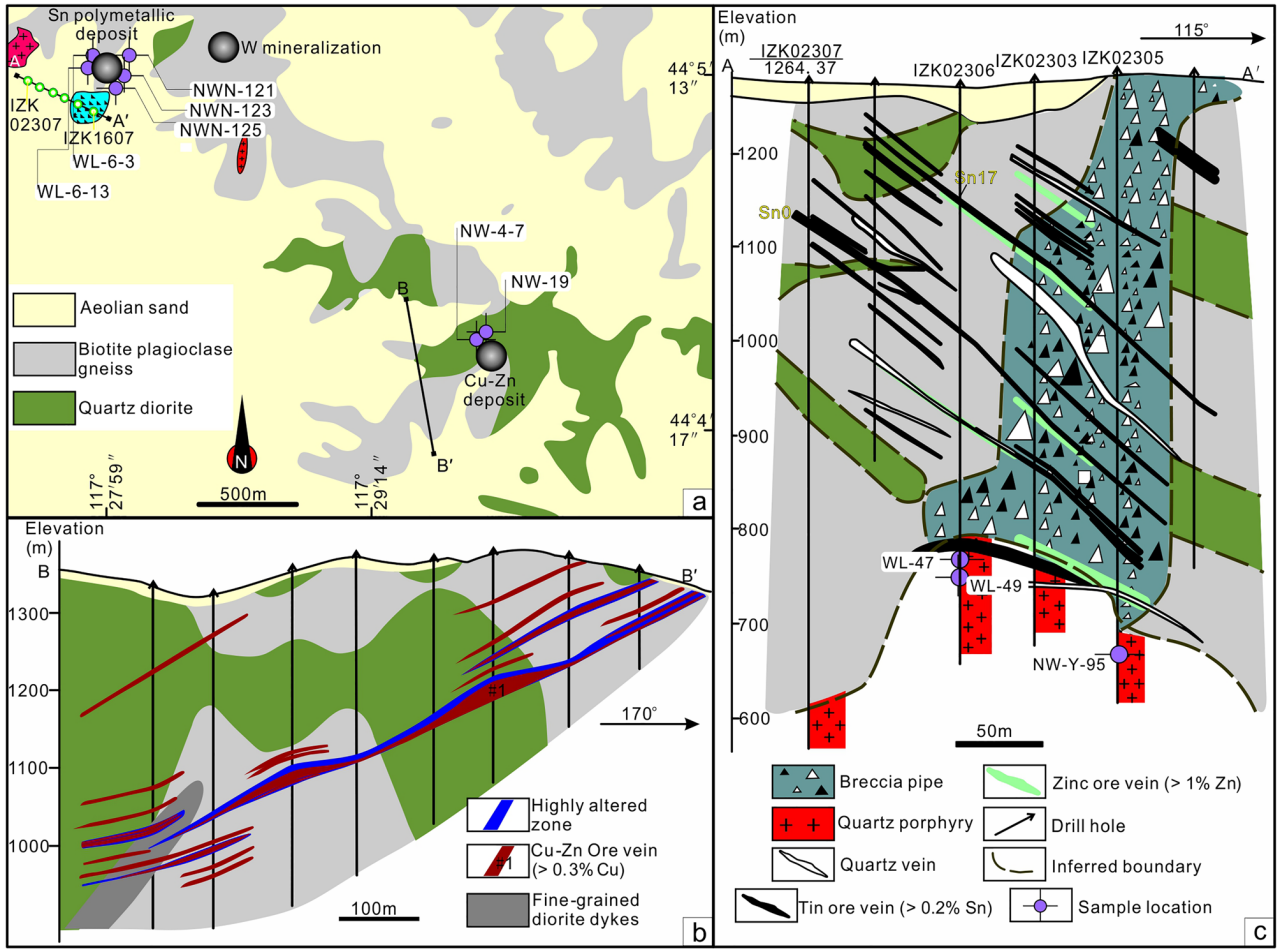
(**a**) Geological map of Weilasituo mining area, Inner Mongolia. (**b**) No. 0 prospection line cross-section of the Weilasituo Cu–Zn deposit (modified after Bureau of Geology and Mineral Resources of Inner Mongolia). (**c**) No. I023 prospection line cross-section of the Weilasituo Sn-polymetallic deposit.

The Weilasituo ore district, at the northern margin of Keshketen Banner in Inner Mongolia, includes the Weilasituo Sn-polymetallic and Weilasituo Cu–Zn deposits, within an exposed area of 8 km^2^. The Bairendaba Ag–Pb–Zn deposit is located ~ 4 km NE from the Weilasituo Cu–Zn deposit (Fig. 1c). These three deposits occur within the Xilin Gol Complex, which in this region is only covered by Quaternary sediments (Fig. 2a). Biotite plagioclase gneiss of the Xilin Gol Complex is the dominant rock type and the main lithology hosting ore (Fig. 2c; Table 1). Intrusive rocks include quartz diorite and quartz porphyry (in some publications also called fine-grained porphyritic alkali-feldspar granite), the latter associated with Sn-W-Li mineralization. Quartz diorite is exposed in the central part of the area and hosts Cu–Zn mineralization (Fig. 2b), zircon ages indicate late Carboniferous crystallization with a weighted-mean SHRIMP U–Pb age of 308.3 ± 4.2 Ma^3^. The quartz porphyry is much younger and has an Early Cretaceous U–Pb zircon age of 135 ± 2 Ma^2^, in agreement with U–Pb cassiterite ages of 138 ± 6 Ma to 135 ± 6 Ma^1^.

**Table 1:** Mineral assemblages, fluid characteristics, and formation age of the different mineralization stages in the Weilasituo and Bairendaba mining areas.

Category	Weilasituo tin-polymetallic deposit	Weilasituo Cu–Zn deposit	Bairendaba Ag-Pb–Zn deposit
Metals and grade	0.8% Sn, 0.83% WO_3,_ 2.59% Zn, 1.25% Li_2_O	5% Zn, 0.8% Cu and 75 g/t Ag	0.7% Cu, 250 g/t Ag, 4.8% Pb, and 6.02% Zn
Mineral association	Cassiterite, wolframite, sphalerite, molybdenite, Fe–Li mica	Sphalerite and chalcopyrite	Sphalerite, chalcopyrite, galena
Strike_and_dip	Strike 25°–41°, SE115°–131° inclin, dip 11–54°	Strike to E–W and dips 8–35° to the north	Strike to E–W and dips 8–50° to the north
Wall rock	Mainly biotite plagioclase gneiss	Quartz diorite and biotite plagioclase gneiss	Quartz diorite and biotite plagioclase gneiss
Fluid temperature	208–367 °C, with salinities of 3.9–8.0 wt% NaClequiv	192–372 °C, with salinities of 4.3–8.7 wt% NaClequiv	176–317 °C, with salinities of 5.3–8.8 wt% NaClequiv
Fluid composition	H_2_O; CH_4_: CO_2_	H_2_O; CH_4_: CO_2_	H_2_O; CH_4_: CO_2_
H–O isotopes	δD_V–SMOW_ = − 109 to − 79‰; δ^18^O_H2O(v–SMOW)_ = + 2.3 to + 4.0‰	δD_V–SMOW_ = − 139 to − 88; δ^18^O_H2O(v–SMOW)_ = + 1.2 to + 5.5‰	δD_V–SMOW_ = − 132 to − 104; δ^18^O_H2O(v–SMOW)_ = + 3.3 to + 6.0‰
Fluid sources	Magmatic and evolved by mixing with local meteoric water	Magmatic and evolved by mixing with local meteoric water	Magmatic and evolved by mixing with local meteoric water
S-Pb isotopes	δ^34^S* = − 4.9‰ to − 2.5‰; ^206^Pb/^204^Pb = 18.34, ^207^Pb/^204^Pb = 15.56, ^208^Pb/^204^Pb = 38.23^6^	δ^34^S = − 3.0‰ to + 3.4‰; ^206^Pb/^204^Pb = 18.33, ^207^Pb/^204^Pb = 15.55, ^208^Pb/^204^Pb = 38.24^5^	δ^34^S = − 4.0‰ ~ + 3.8‰; ^206^Pb/^204^Pb = 18.37, ^207^Pb/^204^Pb = 15.57, ^208^Pb/^204^Pb = 38.29^55^
Source of ore-forming materials	Mainly deep magma	Magma mixed with wall rock material	Magma mixed with wall rock material
Ages	138 ± 6 Ma to 135 ± 6 Ma (cassiterite U–Pb SHRIMP dating)^1^; 129 ± 4.6 Ma (Molybdenite Re-Os dating)^4^	129.5 ± 0.9 Ma (Ar–Ar plateau age of mica)^2^	135 ± 3 Ma (Ar–Ar plateau age of mica)^86^

* The lower δ^34^S may be due to the degassing of SO_2_.

### Concealed quartz porphyry intrusion with associated Weilasituo Sn-polymetalic deposit

The Weilasituo Sn-polymetallic deposit was discovered in 2013. Reserve estimates list 67 economic orebodies, containing a total of 87,281 t Sn, 12,633 t WO_3_, 357,200 t Li_2_O, 16,987 t Zn, and 271 t Mo^34^.

**Figure 3:**
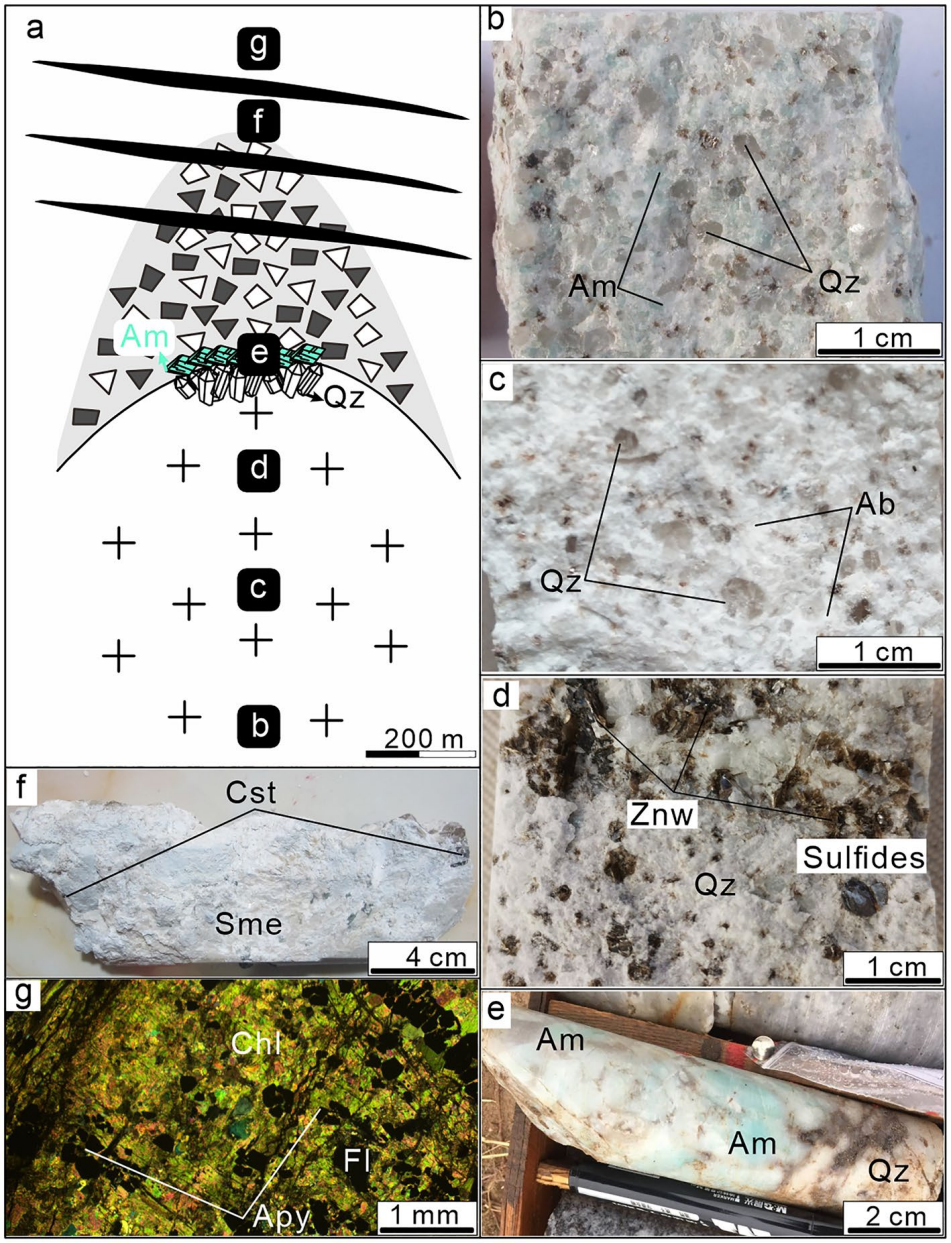
Main alteration types and relative positions within the Weilasituo Sn-polymetallic deposit. (**a**) Schematic sketch of the quartz porphyry intrusion. Lettering on solid black background shows schematically the sampling positions of specimens shown in (**b**–**f**). (**b**) Light sky-blue quartz porphyry with amazonite (K-feldspathization). (**c**) Albite alteration of quartz porphyry with abundant snowball-structured quartz phenocrysts. (**d**) Greisenization at the top of the quartz porphyry body. (**e**) Quartz porphyry in contact with the stockscheider. (**f**) Montmorillonite altered wall rock. (**g**) Chloritized wall rock. *Ab*–albite, *Am*–amazonite, *Apy*–arsenopyrite, *Cst*–cassiterite, *Chl*–chlorite, *Fl*–fluorite, *Qz*–quartz, *Sme*–smectite, *Znw*–zinnwaldite (rock-forming mineral abbreviations^79^).

**Figure 4:**
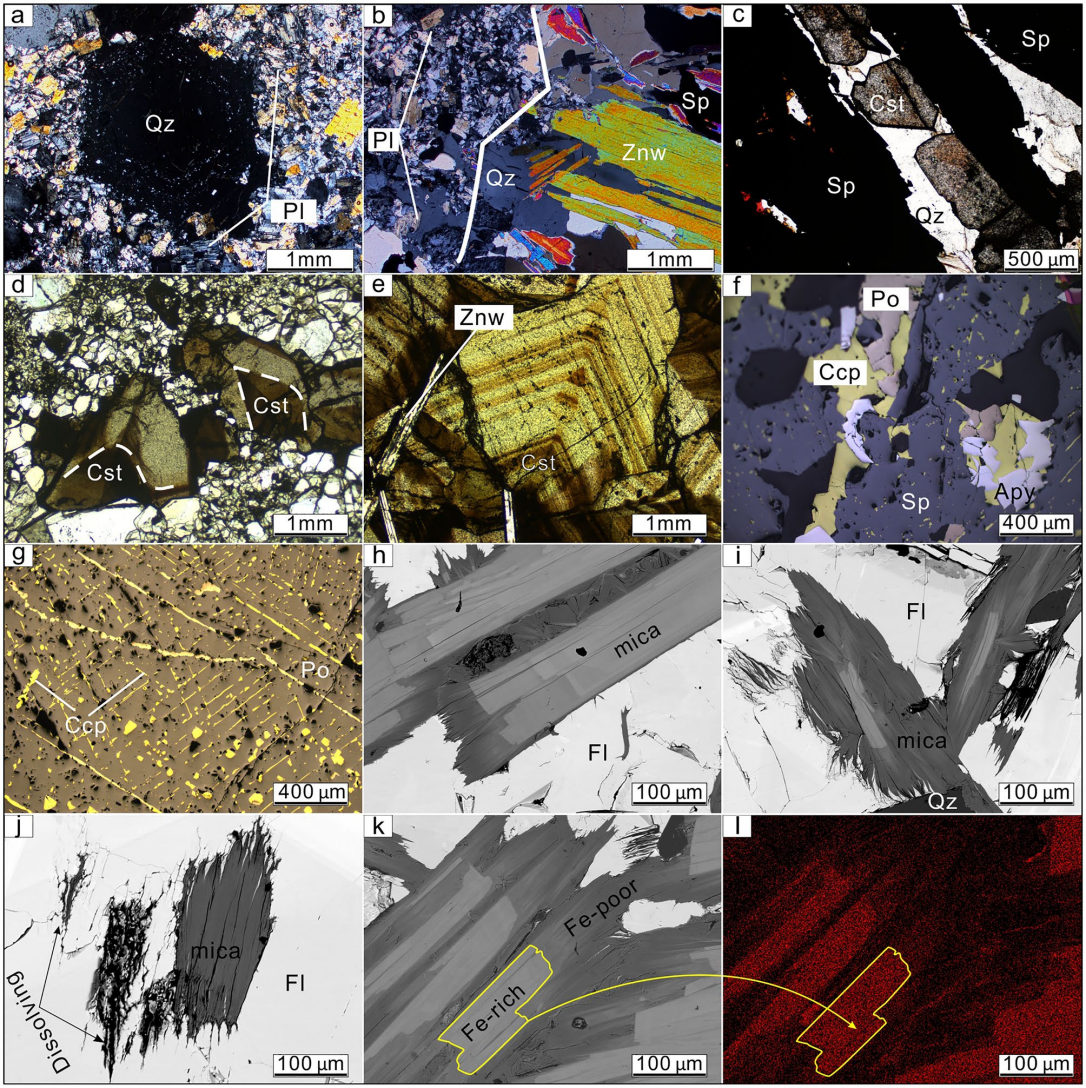
Photomicrographs of minerals from the Weilasituo deposit. (**a**) Snowball-structured quartz phenocrysts. (**b**) Qz + Znw + Sp vein in quartz porphyry. (**c**) Cassiterite associated with sphalerite has advanced white interference color. (**d**, **e**) Cassiterite has regular zonal structure with a dark core and brightly colored rim. (**f**) Association of sphalerite, chalcopyrite, pyrrhotite and arsenopyrite from the Weilasituo Cu–Zn deposit. (**g**) Exsolution of chalcopyrite in pyrrhotite along crystallographic axes. (**h**–**k**) BSE images of mica associated with fluorite from the wall rocks in Weilasituo Cu–Zn deposit indicating growth zones in the early stage (**h**) and strong hydrothermal alteration in later stages. (**l**) SEM–EDS map showing loss of Fe during hydrothermal alteration (the chemical formulae of mica may change from K(AlFe^2+^Li)(Si_3_Al)O_10_(OH,F)_2_ to KAl_2_(Si_3_Al)O_10_(OH,F)_2_). *Apy*–arsenopyrite, *Ccp*–chalcopyrite, *Cst*–cassiterite, *Fl*–fluorite, *Pl*–plagioclase, *Po*–pyrrhotite, *Qz*–quartz, *Sp*–sphalerite, *Toz*–topaz, *Znw*–zinnwaldite.

The concealed quartz porphyry body was identified in drill holes at depth > 400 m below surface (Figs. 2c, 3a). The deepest drillhole intersecting the quartz porphyry reached a depth of ~ 1450 m. Evaluation of this drillhole indicates three facies of quartz porphyry. The quartz porphyry in the deepest area is light sky-blue due to the presence of amazonite, has a massive structure and a porphyritic texture with dominant quartz phenocrysts (Fig. 3b). The matrix is composed of fine-grained quartz and K-feldspar, the accessory mineral assemblage consists of biotite, topaz, and zircon (Fig. 3b). The central part of the intrusion, about ~ 800 m below surface, is albitized and has a porphyritic texture with phenocrysts of quartz and albite occurring in a matrix of albite, quartz and some Fe-Li mica and accessory minerals including zircon (Figs. 3c, 4a). At shallower levels (400–500 m below surface), the intensity of albitization decreases, whereas quartz and mica contents increase (i.e., greisenization), accessory minerals include fluorite, topaz and zircon, Sn-W-Li and sulfide mineralization is present (Fig. 3d). The porphyry belongs to the high-K calc-alkaline series and is strongly enriched in Si, Al, and depleted in Ca, Ti and P. The A/CNK value of quartz porphyry usually ranges from 1.0 to 1.2; most analyses yield a value < 1.1, which is typical for I-type granites. The low values of K/Rb, Nb/Ta, and Zr/Hf of the porphyry reflect a high degree of magma differentiation^1,4^.

At the top of the stock, the quartz porphyry is rimmed by a pegmatite-like stockscheider zone with coarse K-feldspar crystals growing perpendicularly from the contact inside the stock. The stockscheider zone (Fig. 3e) occurs as a shell on top of the parent rock and has a maximum thickness of ~ 4 m. Its upper part is dominated by amazonite and its lower part by quartz. A pipe-shaped cryptoexplosive breccia body extends from the uppermost part of the granite porphyry body toward the surface (Fig. 2c). It is ~ 500 m in length with an elliptical outcrop of 30 m × 20 m at the present surface. The breccia comprises strongly altered clasts of biotite-plagioclase gneiss and quartz diorite in a cement of quartz, mica, and felsic igneous minerals (ESM Fig. 1c).

Three types of mineralization are associated with the quartz porphyry: (1) shallow quartz-cassiterite-sphalerite veins; disseminated ore in (2) breccia pipe and in (3) the upper part of the porphyry intrusion. The deeper part of the deposit is a lenticular body directly at the top of the porphyry stock (Fig. 2c). Eighteen disseminated mineralization zones (14 Sn and 4 Zn mineralization zones) have been delineated, including 7 zones that exceed the cut-off grade (Sn: 0.2%, Zn: 1%). Among these, orebody #Sn200 hosts > 82.6% of the dissemination-type ore reserves. It is 337 m long with a thickness of 10 m in its central part, has an overall strike of 25°, dip of 5° to E. The average Sn grade is 0.23%, with highest Zn and WO_3_ grades of 1.74% and 0.098%, respectively. Stannite, sphalerite, and molybdenite are the dominant ore minerals in this type of mineralization and are associated with cassiterite and wolframite (Table 1).

Breccia-type ore bodies occur in the cryptoexplosive breccia, which has the shape of a steep pipe with an elliptical cross-section 140–300 m in diameter, a long axis strike of 30°, and 640 m vertical extent^34^. Breccia clasts are commonly rimmed by zinnwaldite rich in Li, Nb, and Ta (ESM Fig. 1c). Lithium contents exceed cut-off grade (0.7% Li_2_O) with good prospects for exploitation^34^. Cassiterite, sphalerite and other ore minerals occur within the breccia cement.

The shallow quartz-cassiterite-sphalerite veins form inclined orebodies consisting of densely packed veins, with an overall strike of 25° and dip 11–54° SE (Table 1). A total of 231 quartz veins have been identified so far, 60 of these exceed cut-off grades (Sn: 0.2%, Zn: 1%) for resource/reserve estimation. The #Sn0 Sn-Zn-Mo-W vein contains ~ 66% of the total reserves, and is located in the middle part of the exploration area. It is accessible 130 m below the present surface and is 900 m long with a maximum thickness of 9 m in its central part, with a strike and dip of 13–45/23–25° SE^1^ (Fig. 2c). Cassiterite (ESM Fig. 1e; Fig. 4d), wolframite, and sphalerite (Fig. 4b, c) are the main ore minerals. Quartz, zinnwaldite (ESM Fig. 1a), and smectite (ESM Fig. 1g) are the main gangue minerals. Montmorillonite is widespread in the surrounding rock (Fig. 3f), and abundant zinnwaldite and cassiterite are observed attached to the montmorillonite.

About ~ 1.0 km east to the Weilasituo Sn-polymetallic deposit (Fig. 2a), there is a small tungsten deposit with an average grade of 0.83% that contains 603 t WO_3_. The main tungsten-polymetallic vein (#121–1) has a strike and dip of 35–41/38–52° SE. Wolframite, molybdenite and sphalerite are the main ore minerals (Table 1). The deposit has a molybdenite Re-Os isochron age of 129.0 ± 4.6 Ma^4^, comparable with the crystallization and mineralization age of the Weilasituo Sn-polymetallic deposit and the mineralization age of the Weilasituo Cu–Zn deposit (Table 1).

### Weilasituo Cu–Zn deposit

The Weilasituo Cu–Zn deposit lies ~ 2 km SE of the Weilasituo Sn-polymetallic deposit and contains a 10 Mt ore reserves at grades of 5% Zn, 0.8% Cu, and 75 g/t Ag (Table 1). The deposit is composed of 121 ore bodies, mostly simple quartz veins^5^, two of which outcrop at surface. The host rocks are mainly biotite plagioclase gneiss and locally also quartz diorite (Table 1). Thirty-five ore veins, exceeding cut-off grade (Cu: 0.3%, Zn: 1%, Pb: 0.7%, Ag: 50 g/t), are included in the reserve estimate. Vein size varies from tens of meters to > 1000 m in length and they extend from depths of tens of meters to more than 900 m; vein thickness locally reaches up to > 10 m. Except for some NNE-trending veins, the veins trend nearly E-W and dip to the N with an inclination of 8–35° (Table 1). Among these, the #1 vein is the main orebody (Fig. 2b), accounting for over half the total resource. It mainly contains Zn and Cu, accompanied by Ag and W. The sulfide ore is composed of sphalerite, chalcopyrite, pyrrhotite, arsenopyrite and galena (ESM Fig. 1d; Fig. 4f, g); gangue minerals include quartz and mica, as well as calcite, fluorite, and illite (Fig. 4h–l). Alteration in the main mineralization stage includes silicification and sericitization, as well as the formation of fluorite and topaz. Alteration during late mineralization stages includes carbonatization, chloritization (Fig. 3g) and kaolinization. Silicification and sericitization occur at the contact between quartz veins and plagioclase gneiss, accompanied by flurite and topaz. Carbonation and kaolinization often occur at the contact between plagioclase gneiss and quartz diorite, accompanied by chloritization.

### Bairendaba Ag–Pb–Zn deposit

The deposit is divided into an eastern and a western domain. In the eastern domain, 54 veins were identified and 22 are exploited for Ag, Pb, and Zn. The veins strike almost E–W and are inclined 10–50° to the north. The eastern domain has reserves of 0.9 Mt Zn, 0.45 Mt Pb and 3961 t Ag (Table 1). The #1 vein accounts for nearly 80% of the total ore reserves in the eastern domain^2^. It is 2075 m long with an average thickness of 4.6 m and an average ore grade of ~ 250 g/t Ag, 4.8% Pb, and 6.02% Zn. The dip of the vein gradually changes from 17° in the west to 20° in the deposit center (No. 0 exploration line), and 35° in the east. The western domain includes 167 veins, of which 47 are of exploitable grade: the main vein (No. 3) accounts for ~ 65% of the total reserves in the western domain (0.49 Mt Zn, 0.05 Mt Pb, and 276 t Ag). It is 1200 m long, extends to a depth of ~ 700 m, and has an average thickness of almost 5 m. The veins generally strike nearly E–W, and dip 8–50° to the north. The ore is mostly composed of pyrrhotite, sphalerite, and chalcopyrite, with an average Zn grade of about 5%. In the middle and lower parts of the veins, Cu-rich and/or Cu–Zn ores have average Cu grades of ~ 0.70%, whereas in the west, Ag-Pb–Zn ores have an average grade of 286.6 g/t Ag and 4.11% Pb.

Pre- and syn-ore wall rock alteration is represented by chloritization, silicification, and illite-fluorite alteration. Late-stage carbonatization and fluoritization is recognised. The main sulfides are sphalerite, chalcopyrite, pyrrhotite and galena (Table 1). Gangue minerals are mainly quartz, calcite, and fluorite.

## Samples and results

**Figure 5:**
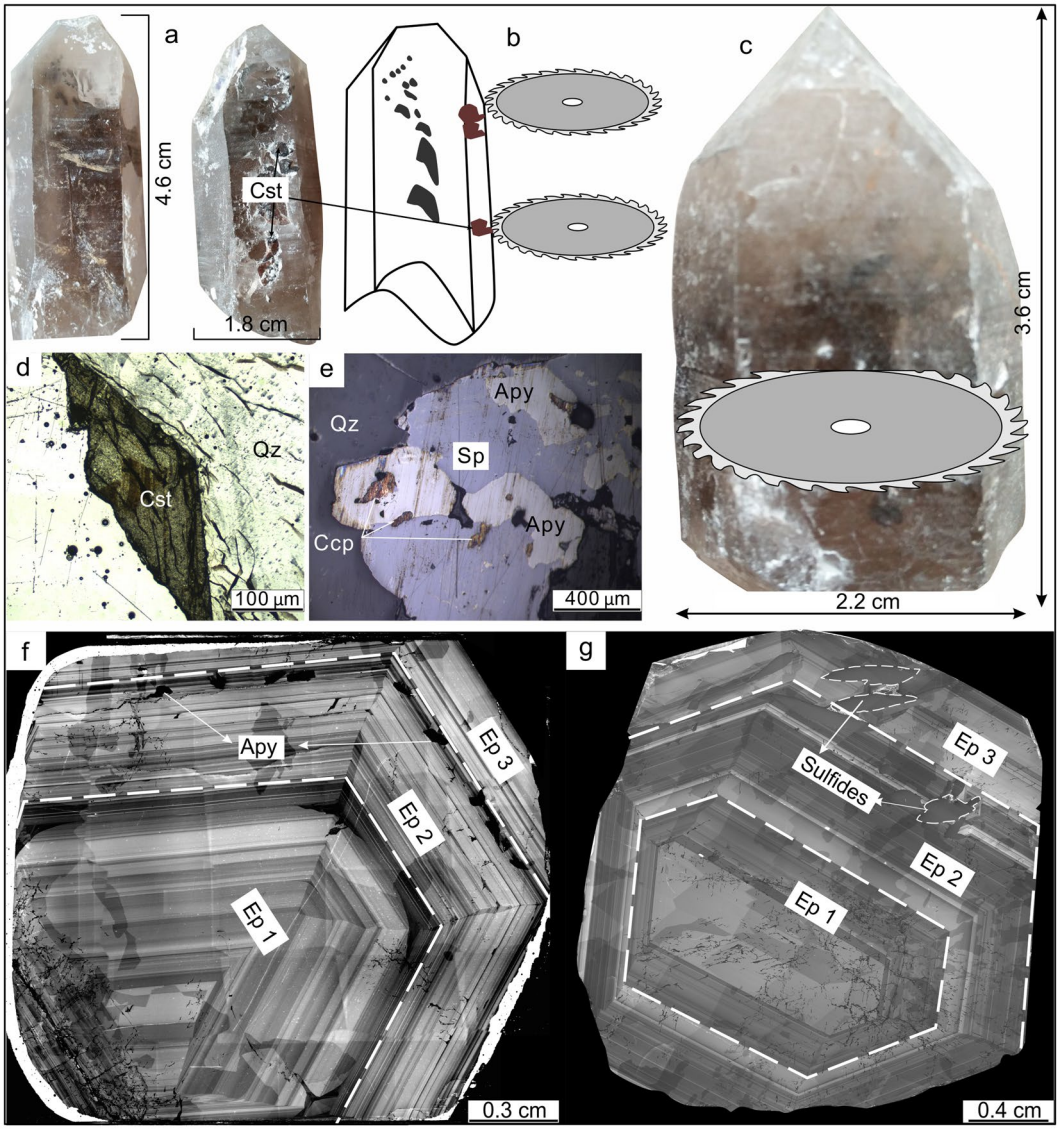
Two quartz crystals from vugs in Sn-Zn veins. (**a**–**c**) Photographs of crystals and position of thin sections of samples WQ-5 and WQ-6, respectively. (**d**) Photomicrographs of sample WQ-5 under orthogonal polarized light. (**e**) Photomicrographs of sample WQ-6 under reflected light. (**f**–**g**) Cathodoluminescence image of sample WQ-5 and sample WQ-6. Ep 1: first episode of fluid flow.

A suite of 38 samples were collected from outcrops, tunnels and drillcores in the Weilasituo deposit, 3 samples were sourced from tunnels in the Bairendaba deposit. Sample locations and characterization is presented in ESM Table 1. These samples originate from all four stages of ore formation^1,4^ described below. In addition to these rock samples, we collected two quartz crystals from vugs adjacent to the Sn-bearing quartz veins of the Weilasituo Sn deposit near the quartz porphyry. These quartz crystals have inclusions of cassiterite and opaque minerals with metallic appearance (samples WQ-5 and WQ-6; Fig. 5a–c). The applied analytical methods included cathodoluminescence imaging, fluid inclusion and Raman studies, hydrogen and oxygen isotope analysis and LA-ICP-MS trace element analysis (see ESM for details).

### Stages of mineralization

According to their different mineral assemblages combined with information from prior studies^1,4^, four stages are identified in the Weilasituo Sn-polymetallic deposit:

1. Magmatic stage (Stage I), including the evolution of the granitic magma, undercooling, and crystallization of the stockscheider (Fig. 3e) followed by crystallization of the quartz porphyry, which contains abundant snowball-structured quartz phenocrysts (Fig. 4a). Only minor amounts of ore minerals (zinnwaldite) precipitated at this stage, gangue minerals are mainly quartz, albite, amazonite, topaz and a small amount of fluorite and zircon.
2. Magmatic-hydrothermal transition stage (Stage II). This stage includes exsolution and accumulation of volatiles from the residual magma, followed by the formation of a cryptoexplosive breccia pipe due to the rapid separation and crystallization of volatiles. The mineralization mainly occurs in the breccia pipe (ESM Fig. 1c), which contains abundant zinnwaldite with a Li_2_O concentration of 4.7 wt%^4^. Zinnwaldite is the major ore minerals are mainly (ESM Fig. 1c) in a mineral paragenesis including quartz, albite, fluorite, topaz, and minor amazonite and zircon (Table 1).
3. High-temperature hydrothermal stage (Stage III), which includes early greisenization at the top of the quartz porphyry body and late quartz veins, is the main mineralization stage in the Weilasituo Sn-polymetallic deposit. The greisenization is associated with zones of disseminated mineralization. Minor disseminations of stannite and sphalerite also formed during this stage. Quartz + zinnwaldite + cassiterite + sphalerite veins were encountered in the 100–400 m interval (Fig. 2c; ESM Fig. 1a), where veins host abundant vugs containing crystals of euhedral quartz, sphalerite, cassiterite and fluorite (ESM Fig. 1b; Fig. 4c–e). Ore minerals are mainly cassiterite, wolframite, sphalerite and minor molybdenite (Table 1). Cassiterite is mainly associated with sphalerite (ESM Fig. 1e; Fig. 4c) and features a regular zonal structure with dark colored cores and brighter rims (Fig. 4d, e), while wolframite is associated with molybdenite. Furthermore, the mineral assemblage includes quartz, fluorite, zinnwaldite, montmorillonite, löllingite, and arsenopyrite (ESM Fig. 1a, b; Table 1). Pyrite is minor in this stage, and the main Fe-bearing minerals are löllingite, arsenopyrite, and zinnwaldite. Chalcopyrite is sometimes observed within quartz (Fig. 5e).
4. Low-temperature hydrothermal stage (Stage IV), representing both the main mineralization stage of the Weilasituo Cu–Zn deposit and a late overprint in the Sn-polymetallic deposit. Sulfide assemblages evolve from sphalerite + löllingite + arsenopyrite in the Sn deposit to sphalerite + chalcopyrite + pyrrhotite + pyrite + arsenopyrite in the Cu–Zn deposit. The main ore minerals are sphalerite and chalcopyrite (ESM Fig. 1d). Futhermore, there is quartz, mica, fluorite, pyrrhotite and arsenopyrite (Table 1). Mica, associated with fluorite in the wall rocks, displays obvious early-stage growth zoning (Fig. 4h).

### Fluid inclusion microthermometry

**Figure 6:**
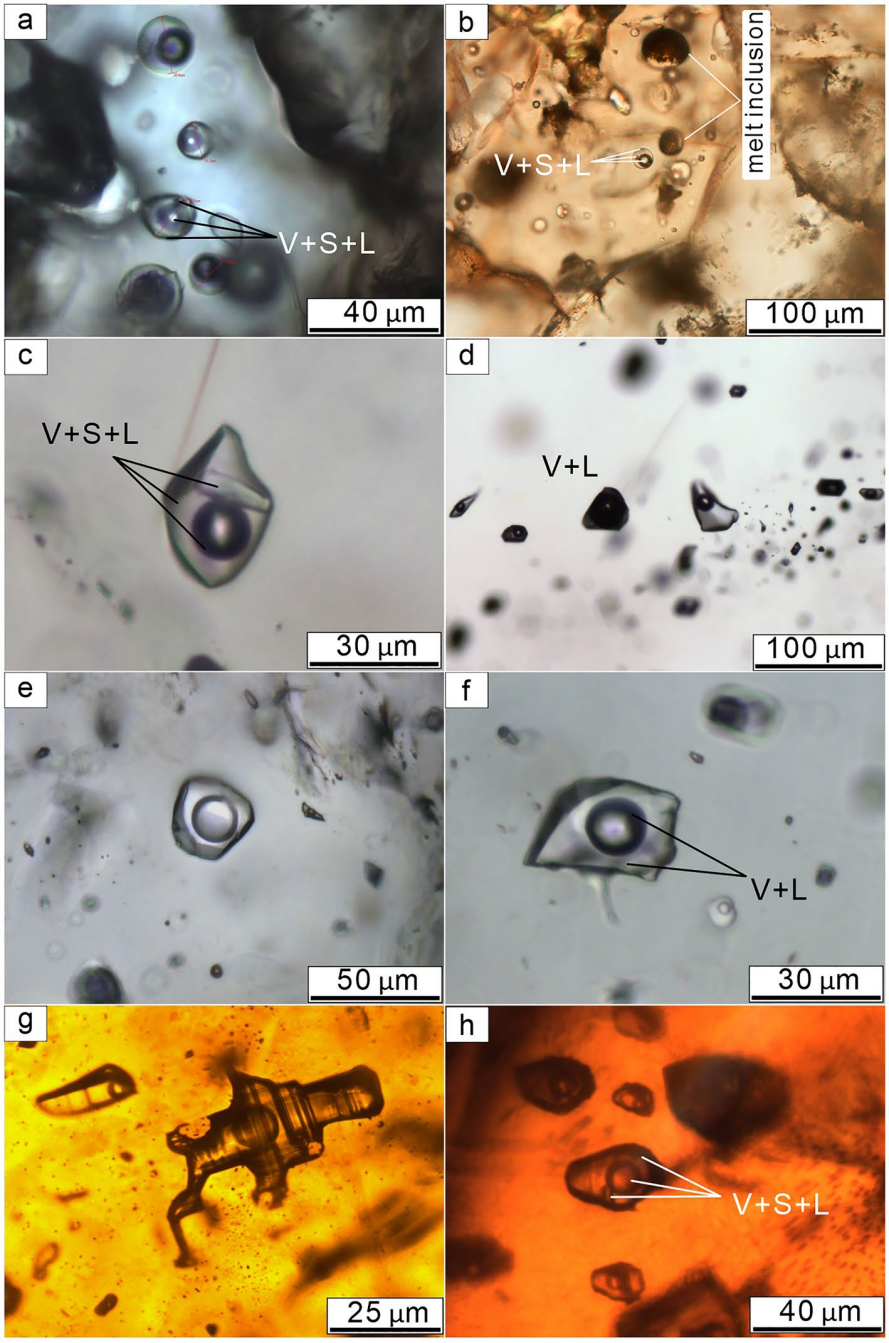
Transmitted light photomicrographs of different types of fluid inclusion in quartz and sphalerite. (**a**) L-V-S inclusions in quartz from the magmatic stage. (**b**) Melt inclusions coexist with fluid inclusions in quartz from the magmatic stage. (**c**) L-V-S inclusions in quartz from early Sn-Zn veins. (**d**) L-rich fluid inclusions coexist with V-rich fluid inclusions in quartz from early Sn-Zn veins. (**e**–**f**) Aqueous L-V inclusions in quartz from late quartz-sulfide veins. (**g**) Aqueous L-V inclusions in sphalerite from early Sn-Zn veins. (**h**) L-V-S inclusions in sphalerite from late quartz-sulfide veins.

Most of the fluid inclusion measurements were carried out on quartz phenocrysts from granite and quartz vein samples. The inclusions commonly show negative crystal shapes and generally have a size between 10 and 35 μm (Fig. 6a–f). Although fluid inclusions are occasionally observed in sphalerite (Fig. 6g, h), most are too small (typically 2–8 μm in diameter) to enable observation of phase changes during freezing-heating. It was, however, possible to obtain homogenization temperatures and salinity data from several large fluid inclusions in Stage III and IV sphalerite (Fig. 6g, h). Two main types of fluid inclusions are distinguished based on phase relations at room temperature: Type I liquid–vapor (LV) two-phase inclusions (Fig. 6d–g); and Type II liquid–vapor-solid (LVS) three-phase inclusions (Fig. 6a–c). Type I fluid inclusions are the dominant type in the Weilasituo ore district. Sporadic minor solid phases occur in all types of fluid inclusions, except for those of the final stage.

Type I fluid inclusions occur in three stages of ore formation (Stages I, III and IV). Compositions change from aqueous two-phase inclusions with 25–50 vol% H_2_O–vapor at room temperature (Stage I) (Fig. 6a, b), through vapor-rich aqueous two-phase inclusions with 33–40 vol% gas phase at room temperature (Stage III) (Fig. 6c, d), to 25–33 vol% gas phase at room temperature (Stage IV) (Fig. 6e, f). Stage III and Stage IV fluid inclusions in quartz are typically 10–30 μm in diameter, whereas in sphalerite, they may reach 40 μm in diameter and generally contain 25–33 vol% vapor (Fig. 6g, h).

**Figure 7:**
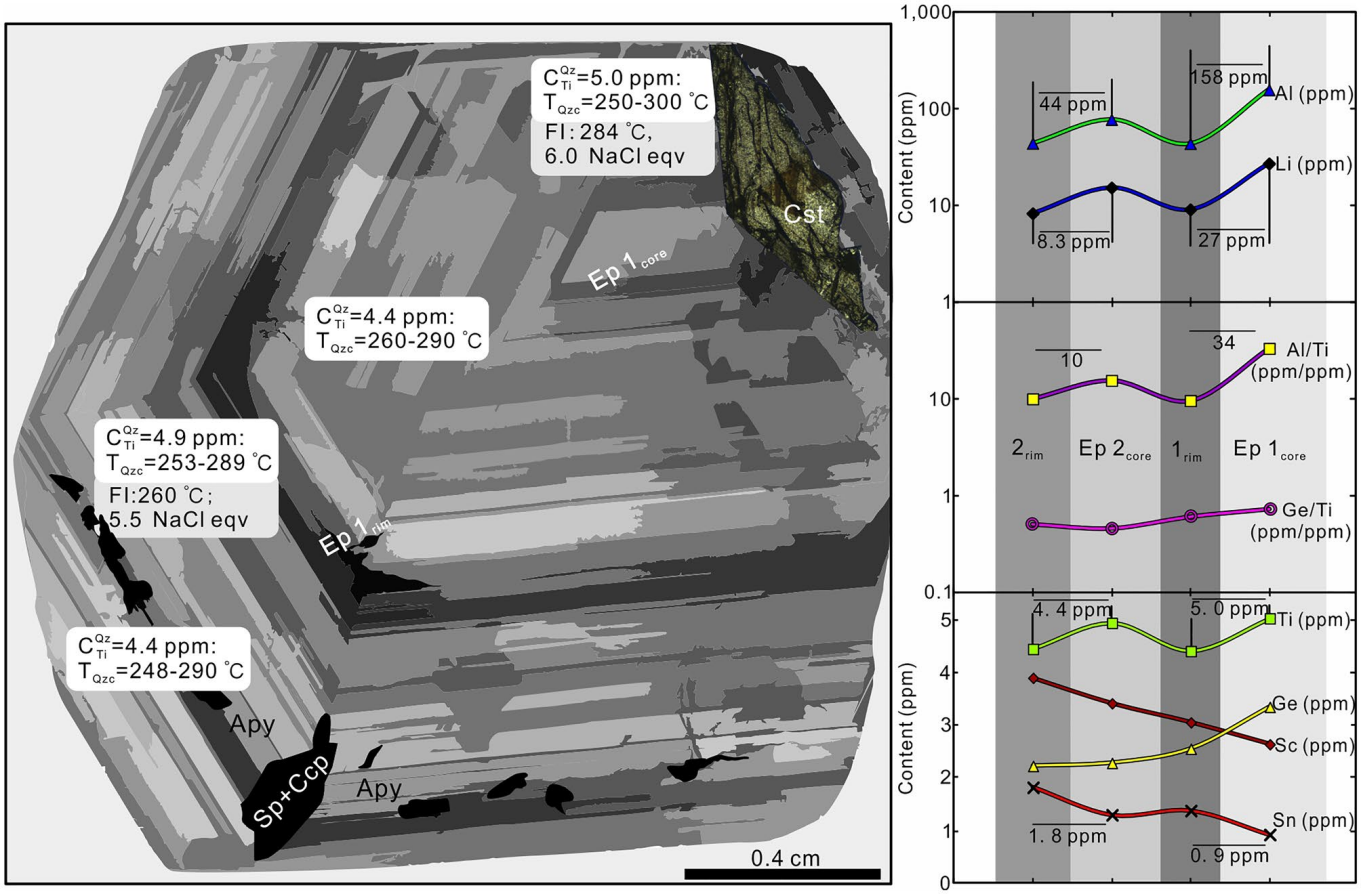
Schematic variation of trace element contents in the quartz crystal sample WQ-5. $\rm C_{\rm Ti}^{\rm Qz}$: average Ti concentrations in the quartz, T_Qzc_: crystallization temperature of quartz. For fluid inclusion microthermometry, it was necessary to remove the polished sample from the carrier glass and break it into an appropriate size. Therefore, trace element analysis of quartz was not conducted along a profile from the core to rim, but on different fragments taken at different distances from the rim. The element profiles were constructed using CL as a guide.

Abundant LV fluid inclusions with 30–35 vol% vapor occur in sample WQ-5. Groups of small (15–25 μm) fluid inclusions occur in the crystal core while individual larger (25–45 μm) inclusions were found in the crystal rim (Fig. 7).

### Th_LV–L_, salinity and Raman spectroscopy

**Figure 8:**
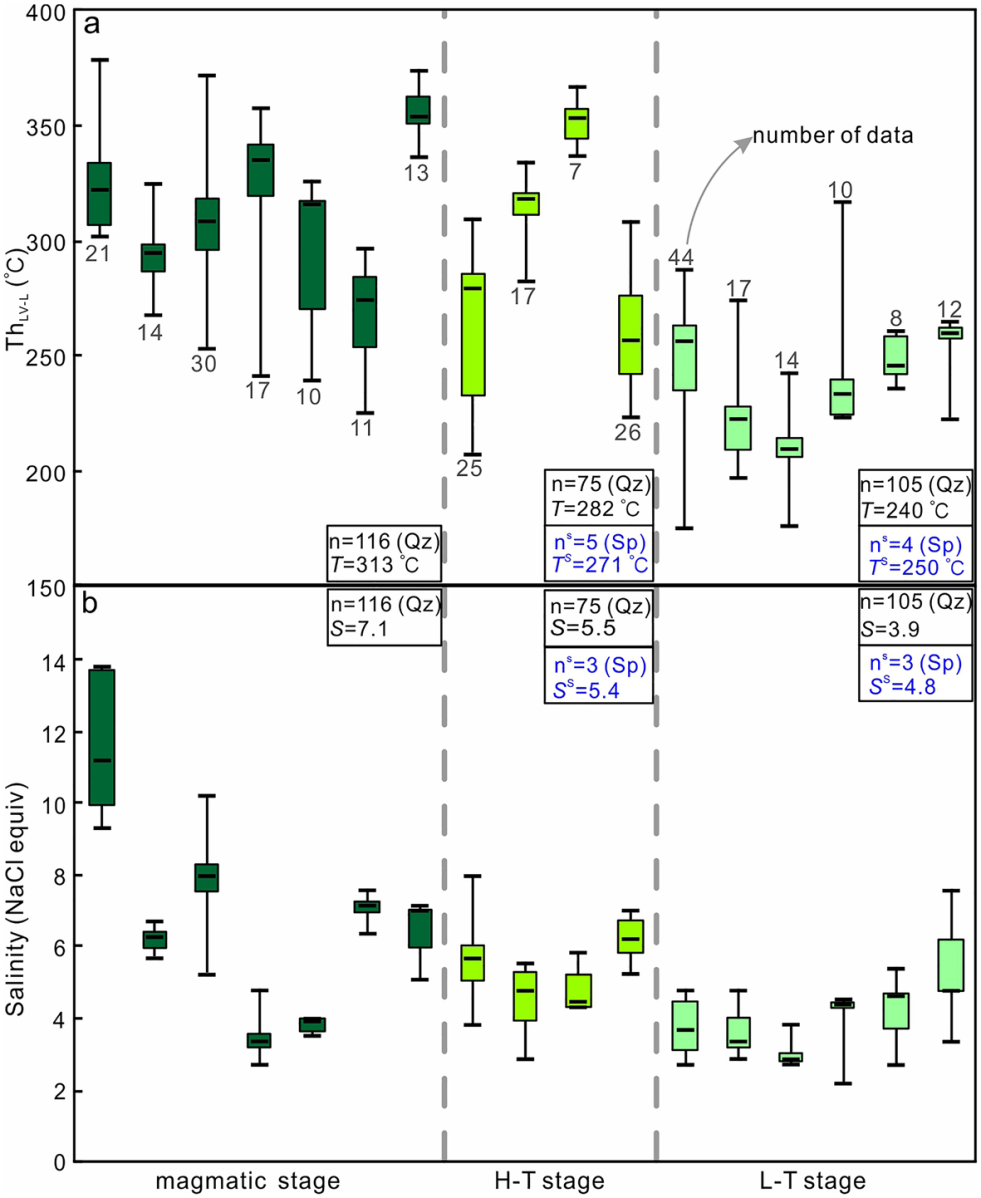
Boxplots showing (**a**) homogenization temperature (Th_LV–L_) and (**b**) salinity of inclusions from different stages of the Weilasituo Sn-polymetallic deposit. n: total number of fluid inclusions. *T* and* S*: average temperature and salinity of fluid inclusions in quartz. *T*^*s*^ and *S*^*s*^: average temperature and salinity of fluid inclusions in sphalerite.

The salinities of primary inclusions were determined from the final ice melting temperatures^35^. Primary Stage I and Stage III inclusions in quartz from the porphyry and from quartz veins containing W- and Sn-minerals have salinities of 2.7–13.7 wt% and 3.9–8.0 wt% NaCl_equiv_, respectively, and respective homogenization temperatures of 226–379 °C and 208–367 °C (Table 2). Stage IV fluid inclusions in quartz show liquid homogenization temperatures of 176–317 °C (mean 240 °C) and salinities of 2.2–7.6 wt% NaCl_equiv_ (mean 3.9 wt% NaCl_equiv_) (Fig. 8a, b). There are only few temperature and salinity determinations for Stage III and IV fluid inclusions in sphalerite. These data are shown for reference in Fig. 8a, b. For sample WQ-5, the mean Th_LV–L_ of the crystal core is 284 °C, consistent with the Th_LV–L_ data of Stage III (Fig. 7), and 260 °C for the crystal rim. The corresponding salinities are 6.0 wt% and 5.5 wt% NaCl_equiv_, respectively (Fig. 7).

**Table 2:** Homogenization temperature of fluid inclusions from different mineralization stages of the Weilasituo Sn-W-Li deposit.

Sample	Stage	Mineral	Type (n)	*t*_m–ice_/(-°C)	*t*_h-tot_/°C		*w*(NaCl_ep_)/%	
					Range	Mean	Range	Mean
WL-13	I	Qz	L(21)	6.1–9.8	304–379	325	9.3–13.7	11.9
WL-15	I	Qz	L(14)	3.5–4.2	268–325	295	5.7–6.7	6.3
WL-16	I	Qz	L(30)	3.2–6.8	254–372	309	5.3–10.2	7.5
WL-27	I	Qz	L(13)	3.1–4.5	337–374	355	5.1–7.2	6.5
WL-40	I	Qz	L(17)	1.6–2.9	242–358	326	2.7–4.8	3.4
WL-49	I	Qz	L(10)	2.1–2.4	240–326	298	3.5–4.0	3.9
NW-Y-95	I	Qz	L(11)	4.2–4.8	226–297	270	6.7–7.6	7.1
NWN-125	III	Qz	L(26)	3.2–4.4	224–309	260	5.3–7.0	6.2
WL-21	III	Qz	L(25)	2.3–5.1	208–310	263	3.9–8.0	5.6
NWN-123	III	Qz	L(7)	2.6–3.6	337–367	352	4.3–5.9	4.8
WL-41	III	Qz	L(17)	2.4–3.4	303–334	315	4.0–5.6	4.6
NWN-121	IV	Qz	L(12)	2.0–4.8	223–265	255	3.4–7.6	5.3
NW-Y-12	IV	Qz	L(8)	1.6–3.3	236–258	249	2.7–5.4	4.2
WL-45	IV	Qz	L(17)	1.7–2.9	198–274	224	2.9–4.8	3.6
WL-46	IV	Qz	L(44)	1.6–2.9	176–288	250	2.7–4.8	3.8
NWN-127	IV	Qz	L(10)	1.3–2.7	224–317	241	2.2–4.4	4.2
WL-24	IV	Qz	L(14)	1.7–2.3	176–243	210	2.9–3.9	3.0
WQ-5	WQ-5_Rim_	Qz	L(14)	2.8–3.9	251–272	260	4.7–6.3	5.5
WQ-5	WQ-5_Core_	Qz	L(13)	3.4–3.9	264–336	284	5.6–6.3	6.0

n, the number of fluid inclusions.

Raman spectra of five representative Type I and Type II fluid inclusions are shown in ESM Fig. 2. Most spectra show broad peaks for water and host quartz (464 cm^−1^) (Fig. ESM Fig. 2a–d), and sharp peaks for CH_4_ at 2918 cm^−1^ (ESM Fig. 2a, Stage I) and 2915 cm^−1^ (ESM Fig. 2d, Stage III). Peaks at 3077 cm^−1^ (C_6_H_6_) in Stage III inclusion (ESM Fig. 2e) indicate alkylation in the early and middle stages of fluid evolution. Sharp peaks at 1091 cm^−1^ in Stage I fluid inclusions (ESM Fig. 2b) are typical for carbonates. Fluids from Stage I inclusions show broad water peaks. The sharp peak at 877 cm^−1^ (ESM Fig. 2c) may indicate that the liquid contains F-bearing phase.

### H–O isotopes

Oxygen isotope data for 15 quartz samples and hydrogen isotope data for 14 quartz samples are presented in ESM Table 2. Two Stage I samples show δ^18^O_Q (v–SMOW)_ values of + 12.6 and + 12.8‰, corresponding to calculated δ^18^O_H2O (v–SMOW)_ values of + 5.5‰ and + 6.2‰. The δD_v–SMOW_ value of a single Stage I sample is − 67‰.

**Figure 9:**
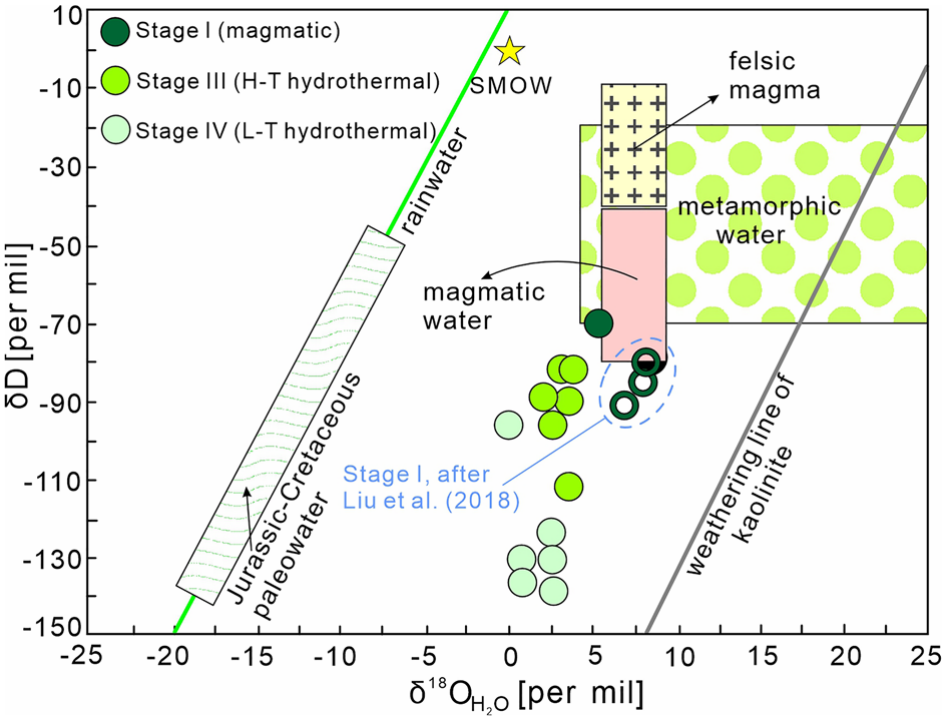
δ^18^O_H2O_‰ versus δD‰ diagram^13,43^ for mineral samples from the Weilasituo Sn-polymetallic deposit. Reference fields: SMOW–Standard Mean Ocean Water^80^; rainwater line^81^; Kaolinite line^82^; Jurassic–Cretaceous paleowater^83,84^; magmatic and metamorphic water^48,80^; felsic magma^85^.

Seven Stage III samples were analyzed. The δ^18^O_Q (v–SMOW)_ values fall within the range + 9.1‰ to + 11.7‰ and the calculated δ^18^O_H2O (v–SMOW)_ values lie between + 2.3‰ and + 4.0‰ (Fig. 9). The δD_v–SMOW_ values are around − 90‰, except for sample WL-18 which has a δD_v–SMOW_ value of − 109‰. The δD_v–SMOW_ and δ^18^O_Q (v–SMOW)_ values decrease from Stage I to Stage III (Fig. 9).

The δ^18^O_Q (v–SMOW)_ the δD_v–SMOW_ values of Stage IV samples fall in the range from + 11.3‰ and + 12.0‰, whereas those from Stage IV samples are around − 130‰ (except for sample WL-24 with a δD_v–SMOW_ value of − 93‰) (Fig. 9).

### Sphalerite geochemistry

Representative sphalerite samples from the Weilasituo Sn and Cu–Zn deposits including quartz-vein type, porphyry-type ore, and quartz crystals from vugs, and three quartz-vein ore samples from the Bairendaba Ag–Pb–Zn deposit were analyzed by LA-ICP-MS. Results (142 spot analyses) are presented in ESM Table 3.

**Figure 10:**
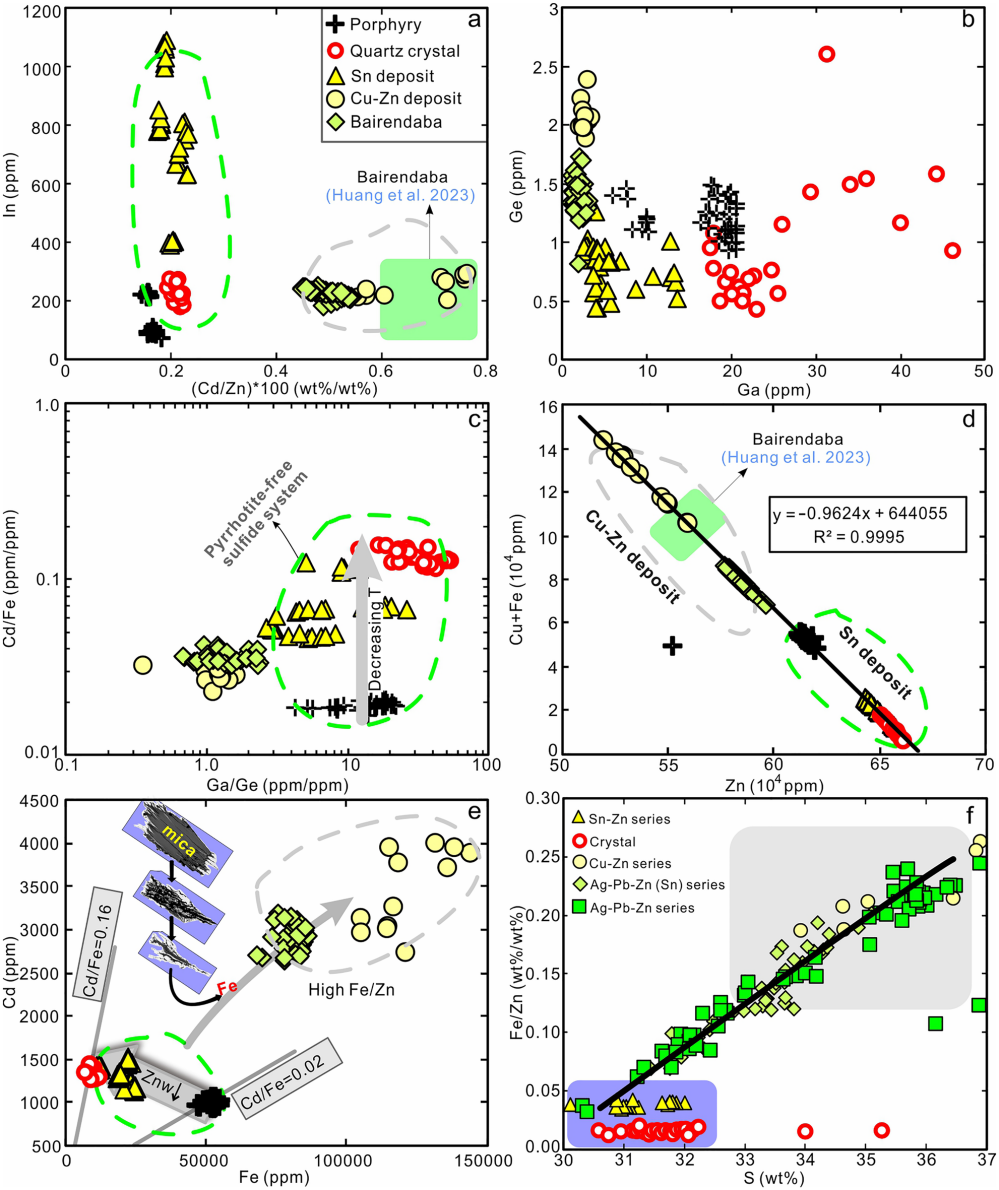
Binary diagrams for major and trace element contents of sphalerite from the Weilasituo and Bairendaba deposits (**a–e**) and chemical variation of sphalerite from different types of deposit (**f**). (**a**) In vs. (Cd/Zn)*100. (**b**) Ge vs. Ga. (**c**) Cd/Fe vs. Ga/Ge. (**d**) Cu + Fe vs. Zn. (**e**) Cd vs. Fe. (**f**) Fe/Zn vs. S (wt.%), Cu–Zn series: quartz-vein sphalerite from the Weilasituo Cu–Zn deposit; Sn-Zn series: quartz-vein sphalerite associated with cassiterite in the Weilasituo Sn-polymetallic deposit (the data of Cu–Zn, Sn–Zn series and sphalerite from the crystal are original work reported here, the data of Ag–Pb–Zn (Sn) and Ag–Pb–Zn series are unpublished data of the authors.

Sphalerite from all samples is enriched in Cu (117 ppm–1.87 wt.%), Fe (0.91–14.4 wt%), Mn (182–1580 ppm), and Cd (893–4050 ppm). Sphalerite from the Weilasituo Cu–Zn and Bairendaba Ag–Pb–Zn deposits have higher Cu + Fe, Mn, Cd contents and higher Fe/Zn ratios than sphalerites from the Weilasituo Sn deposit (Fig. 10d–f; ESM Fig. 3). In the latter, there is a systematic decrease of Cu and Fe content from porphyry-type sphalerite to quartz-vein-type sphalerite (Fig. 10d).

The concentrations of trace elements are highly variable, e.g. 0.08–312 ppm Sb, 0.44–5.18 ppm Ge, 1–8830 ppm Sn, and 1–1120 ppm In (Fig. 10a). Compared to the Weilasituo Sn deposit, the sphalerite from the Weilasituo Cu–Zn and Bairendaba Ag–Pb–Zn deposits has higher Ge contents. Different types of sphalerite from the Weilasituo Sn deposit show similar Ge content (Fig. 10b). Quartz-vein-type sphalerite has lower Ga contents than sphalerite from the quartz crystal and porphyry-type mineralization (Fig. 10b). Ga/Ge ratio of sphalerite decreases from Weilasituo Sn deposit to Cu–Zn deposit and shows a positive correlation with the Cd/Fe ratio (limited to the hydrothermal system) (Fig. 10c). Sphalerite from the quartz crystal has the highest Sn content, whereas quartz-vein-type sphalerite from the Weilasituo Sn deposit has the highest In contents (Fig. 10a).

### Cathodoluminescence textures and quartz chemistry

CL images show growth zoning for the quartz crystals WQ-5 and WQ-6 from the vug (Fig. 5f, g). Inclusions show a regular pattern with cassiterite inclusions in the crystal core (Fig. 5d), sphalerite, chalcopyrite and arsenopyrite inclusions in the transitional zone (Fig. 5e, g), and arsenopyrite in the crystal rim (Fig. 5f).

A total of 136 spot analyses were conducted, with 6–15 analyses of each quartz type in three representative rock samples (quartz porphyry, breccia pipe, quartz vein), and 86 analyses from two quartz crystals from vugs. Data are presented in ESM Table 4.

**Figure 11:**
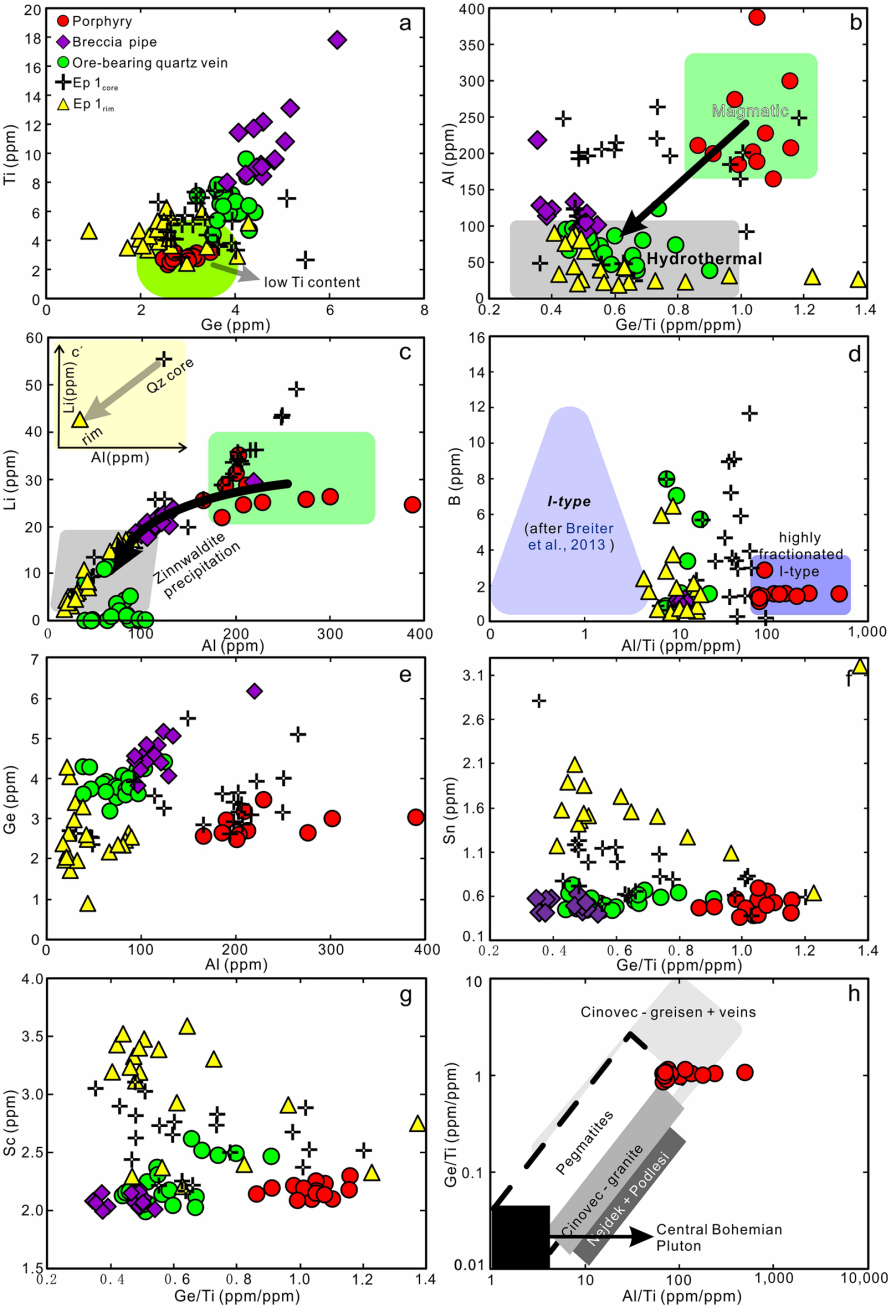
Trace element contents in quartz from different rock types of the Weilasituo deposit. (**a**) Ti vs. Ge. (**b**) Al vs. Ge/Ti. (**c**) Li vs. Al. (**d**) B vs. Al/Ti. (**e**) Ge vs. Al. (**f**) Sn vs. Ge/Ti. (**g**) Sc vs. Ge/Ti. (**h**) Ge/Ti vs. Al/Ti^25^.

Titanium contents in quartz (2.3–17.8 ppm, Fig. 11a) decrease from breccia pipe (8.0–17.8 ppm Ti) to quartz vein (4.4–9.6 ppm), quartz crystals from vugs (2.4–7.4 ppm Ti), and phenocrysts in quartz porphyry (2.3–3.2 ppm). Concentrations of Ge (Fig. 11e) show a similar trend with decreasing contents from breccia pipe (3.8–6.2 ppm) to quartz vein (3.2–4.4 ppm), granite porphyry (2.6–3.5 ppm), and quartz crystal (1.2–5.5 ppm). The Ge and Ti contents of quartz broadly correlate positively and the compositional ranges covered by the different types of quartz partially overlap (Fig. 11a, e). The Al content in quartz from quartz porphyry (165–695 ppm) is generally higher than in the other quartz types (mostly < 200 ppm and as low as 14.7 ppm in some cases; Fig. 11b). The Al contents of the quartz crystals from vugs range from 14.7 to 263 ppm, typically with higher concentrations in the core than the rim (Fig. 11b). Lithium contents of our samples range from 16–35 ppm in magmatic quartz to 0.05–29 ppm in hydrothermal quartz (Fig. 11c). Note that the cores of quartz crystals have very high Li contents (13.5–495 ppm Li). Our samples show Ge/Ti values in the range 0.86–1.16 for magmatic quartz (Fig. 11h), but only 0.19–1.0 for most hydrothermal quartz (Fig. 11b).

The contents of Sn, Sc, and B in quartz are low (Fig. 11d, f, g), typically < 1, < 3, and < 8 ppm, respectively. Higher contents of these elements are only observed in quartz crystals from vugs, which display markedly higher Sn (up to 3.1 ppm) (Fig. 11f) and Sc (up to 4.9 ppm) (Fig. 11g) in the rim. Boron contents of quartz range from 0.4 to 11.7 ppm with no clear distinction between core and rim (Fig. 11d).

## Discussion

### Properties and evolution of ore-forming fluids

From the magmatic to late hydrothermal stage, the homogenization temperatures of fluid inclusions decreased from relatively high temperature (313 °C, Fig. 8a) to low temperature (240 °C, Fig. 8a) and from relatively high salinity (7.1 wt% NaCl_equiv_, Fig. 8b) to low salinity (3.9 wt% NaCl_equiv_, Fig. 8b). Raman data show that the main ore-forming fluids in Stage I and III have H_2_O–NaCl–NaF ± CO_2_ ± CH_4_ compositions. The CH_4_ Raman signal is conspicuous, whereas the CO_2_ signal is weak, reflecting the relatively reduced nature of the ore-forming fluids. Furthermore, CH_4_ was previously shown to be ubiquitous in fluid inclusions from the Weilasituo Cu–Zn and Bairendaba Ag–Pb–Zn deposits^5,6^ (Table 1). The occurrence of falkmanite (Pb_5_Sb_4_S_11_) reported from the Bairendaba Ag–Pb–Zn deposit^36^ is further evidence for reducing conditions. The origin of CH_4_ can be related to microbial activity, thermal decomposition of organic matter, or abiogenic sources, such as the mantle or Fischer–Tropsch-type (FTT) reactions (reaction of CO_2_ or CO with H_2_)^37,38^. The temperature of microbial activity^39^ (< 120 °C) is considerably lower than the mineralization temperature in the Weilasituo mining area, suggesting that the effect of microbial activity on the formation of CH_4_ is negligible. It is hard to envisage how CH_4_ from deep mantle degassing might be incorporated into ore-forming fluid^40^ (CH_4_ reacts with O_2_ leading to the formation of CO_2_), it cannot be retained because of oxidation by Fe^3+^^41^. Formation of large quantities of CH_4_ by FTT reactions requires large volumes of hydrogen as well as catalysts like FeNi and FeCr oxides^42^, which may be present in mafic, but not in felsic rocks. Moreover, if all CH_4_ comes from the FTT reaction, higher hydrocarbons like C_6_H_6_ (ESM Fig. 2e) and C_2_H_6_ should not occur in extracted gas^37^. To sum up, we believe that the source of CH_4_ is mainly organic, and only partly originates from FTT reactions. Carbonaceous slates of the Upper Permian Linxi Formation (Fig. 1c), an important hydrocarbon source horizon in the SGXR, may represent one plausible CH_4_ source.

The hydrogen and oxygen isotope data (Fig. 9) indicate that samples from Stage I are within or near the field of magmatic fluids, indicating that the ore-forming fluid may be derived from magma. Whereas samples of Stage III and Stage IV fall below the field of magmatic fluids, showing lower δD values than Stage I fluids. In addition to changes in *f*O_2_ of ore-forming fluid and the influence of CH_4_ (in natural hydrothermal systems this effect is typically small^43^), there are several processes that may cause a decrease of δD values. These include addition of high-altitude meteoric water^43,44^, magma degassing^45,46^, boiling with loss of the vapor phase^45,47^, and mixing with external fluids, including formation water from host-rocks^48^.

The formation of a breccia pipe extending from the upper part of the granite porphyry body supports the important role of magma degassing during the magmatic-hydrothermal transition (Stage II). Moreover, in addition to a decrease in δD values, δ^18^O_H2O (v–SMOW)_ values also decreased slightly from Stage I to Stage III. Our fluid inclusion study provide evidence for fluid boiling in Stage III (Fig. 6d). Thus, the depleted δD values of the Stage III fluid can be attributed to magma degassing during Stage II and fluid boiling in Stage III. Magma degassing and fluid boiling cannot, however, explain the markedly depleted δD values (− 136‰) in Stage IV fluid (Fig. 9). The high CH_4_ concentrations in the fluids indicate the addition of external fluid to ore-forming fluids, which is confirmed by previous He-Ar isotopic data of sulfide minerals from Stage III and IV showing higher ^40^Ar/^36^Ar ratios than saturated rainwater but lower ^40^Ar^*^/^4^He ratios than crustal and mantle fluids^4^. The addition of external fluids can lead to a decrease in both the δD and δ^18^O values of the fluid. However, δ^18^O values show small displacement from Stage III (mean δ^18^O value: + 3.4‰) to Stage IV (mean δ^18^O value: + 1.8‰). Therefore, the continuous decrease of δD values from Stage III to Stage IV fluids could be interpreted as resulting from mixing with small amount external fluids that could be derived from the wall rocks and may have contributions of meteoric water.

### Tracing ore-forming processes: insights from sphalerite geochemistry and ***f***S_2_ evolution

Ions that have a similar radius as Zn^2+^ (0.60 Å) may easily substitute into sphalerite in tetrahedral coordination^30^. Cd^2+^ (0.78 Å), Mn^2+^ (0.66 Å), and Co^2+^ (0.58 Å) are all typically enriched in sphalerite relative to coexisting minerals^28^. Cu^+^ (0.60 Å) is preferentially incorporated into sphalerite via the coupled substitution Cu^+^  + X^3+^ ↔ 2Zn^2+^^49^, where trivalent X is commonly In or Sb^30^. Differences in concentrations of these substituting elements may reflect their variable temporal or spatial availability. Sphalerite from the central Sn-Zn veins in Weilasituo Sn deposit has higher In contents than sphalerite from the Weilasituo Cu–Zn and Bairendaba Ag–Pb–Zn deposits (Fig. 10a), possibly indicating that the early Sn-Zn veins formed from hydrothermal fluids with higher In content.

Sphalerite from different mineralization types within the Weilasituo Sn deposit, a pyrrhotite-free sulfide system, show a systematic decrease in Fe and increase in Cd content from porphyry-type to quartz vein-type mineralization and individual sphalerite inclusions in quartz (Fig. 10e). In contrast, sphalerite from the Weilasituo Cu–Zn and Bairendaba Ag-Pb–Zn deposits, which both carry pyrrhotite-bearing sulfide assemblages, shows elevated Fe and Cd (Fig. 10e). Factors controlling the Fe content in sphalerite, in addition to Fe availability and Py/Po buffering, include pressure and temperature. For instance, in pyrrhotite-free sulfide systems, the FeS content in sphalerite increases with fluid temperature^29^, whereas the Cd/Fe ratio decreases^50^.

Sphalerite from the Weilasituo Sn deposit show a systematic variation in Cd/Fe ratio from 0.02 (porphyry type), 0.05–0.12 (quartz-vein type), to 0.12–0.16 (inclusions in the quartz crystal), suggesting a temperature decrease (Fig. 10c, e). The increase of Cd/Fe with decreasing temperature may also be affected by the precipitation of Fe-rich zinnwaldite (which contains ~ 6.5 to 10.3 wt% FeO)^4^ during the magmatic-hydrothermal transition stage.

Sphalerite from Sn-Zn veins in the central part of the Sn deposit contains 2.09 wt% Fe (average value) and the fluid inclusions in sphalerite homogenize at 271 °C. However, sphalerites from Cu–Zn veins in the peripheral Cu–Zn deposit have much higher Fe contents (up to 12.15 wt%), while displaying similar homogenization temperatures of ~ 250 °C (Fig. 10d, e). These major differences in Fe content are not explained by temperature differences, but may have two alternative explanations. First, pyrrhotite-dominant sulfide systems buffered by Py/Po typically contain sphalerite with very high Fe content^51,52^. Second, availability of Fe may control the distribution of Fe-sulfides, with Fe being mainly hosted in minerals such as löllingite, arsenopyrite and zinnwaldite in the Sn deposit (Table 1) and in pyrrhotite in the peripheral Cu–Zn deposit (Table 1), where Fe is more available. The different Fe availability may be contributed by either of two processes.

(i) Magma degassing and boiling during formation of the breccia pipe released vapor-rich fluids with higher Fe content forming a distal Cu–Zn deposit, whereas the remaining liquid-rich fluid formed the Sn-Zn deposit. However, vapor-rich fluid inclusions account for only a small proportion in the Weilasituo area^5,6^. Additionally, Fe and Zn preferentially partition into a Cl^–^-bearing liquid-rich fluid, whereas Cu is preferentially found in the HS^–^-bearing vapor-rich fluid during phase separation^53,54^ (Cu concentrations remain higher in the coexisting liquid). Although part of metal endowment of the vapor-rich fluid could migrate further, that amount was insufficient to form peripheral Cu–Zn and Ag–Pb–Zn deposits (Table 1). We can thus rule out this possibility as unlikely.

(ii) Magmatic fluids had lower Fe contents than late fluids that had mixed with an external Fe-rich source. Our hydrogen and oxygen isotope data indicate the contribution of external fluids derived from the wall rocks in Stage IV. Stable (S-Pb) isotope data (Table 1) indicates that the source of ore-forming materials in the Weilasituo Cu–Zn and Bairendaba Ag–Pb–Zn deposits were mainly magmatic with some contribution from wall rocks^5,55^. The biotite plagioclase gneiss, the main ore-host, contains 3–6 wt% ^T^FeO, while the quartz porphyry is Fe-poor (~ 0.3 wt% ^T^FeO)^4^. Petrographic evidence shows that mica from the wall rock of Cu–Zn mining area underwent strong late-stage alteration, releasing a large amount of Fe (Fig. 4h–l). In addition, the mica accompanied by sphalerite or galena from the Cu–Zn deposit contains 2169–3772 ppm Zn but extremely low Cu (< 1 ppm Cu)^7^. Therefore, external fluids that altered or dissolved mica from wall rocks (Fig. 4j) may be an important source of Fe, potentially also Zn in the late stage of mineralization. The trace element content characteristics of sphalerite from the Bairendaba mining area are consistent with those of the Weilasituo Cu–Zn deposit suggesting similar formation processes or the involvement of similar sources (Fig. 10a–e).

### Evolution of quartz compositions and Ti-in-quartz geothermobarometry

Quartz has a highly variable trace element composition spanning the fields of magmatic quartz (quartz porphyry) and hydrothermal quartz (quartz veins) (Fig. 11b–e). Magmatic quartz shows extremely low Ti contents (2–4 ppm) and higher Al and Li, whereas hydrothermal quartz has higher Sn and Sc contents. The Al and Li contents in quartz decrease gradually from the quartz porphyry, through the breccia pipe, to the quartz veins (Fig. 11b, c), indicating that Al and Li availability diminished as the magma-hydrothermal system evolved.

The quartz phenocrysts from the porphyry have lower Ti contents than those from most rare-metal granites (mostly 20–110 ppm Ti)^21^. The quartz porphyry has low bulk-rock Ti contents (< 0.01 wt%) and low K/Rb, Nb/Ta, and Zr/Hf values^4^. These very low Ti contents may reflect the higher differentiation of the quartz porphyry^21^, or alternatively, a slower rate of quartz precipitation^27^.

Lithium contents are strongly correlated with Al contents in all types of quartz (Fig. 11c). The Li/Al molar ratios are 0.4–0.6 in magmatic quartz but 0.6–0.8 for most hydrothermal types. This is indicative of the coupled substitution shifting from Si^4+^  ↔ Al^3+^  + Li^+^_0.4–0.6_ (X^+^)_0.4–0.6_ to Si^4+^  ↔ Al^3+^  + Li^+^_0.6–0.8_ (X^+^)_0.2–0.4_ during magmatic-hydrothermal evolution, and that the monovalent X^+^ was most likely H^+^^56,57^. Variation of Li and Al concentrations in quartz are most probably related to the medium from which it crystallized (either melt or fluid) as well as pressure and temperature conditions. The Li concentration in the coexisting phases (melt, fluid and minerals) can be expected to be temperature (-pressure) dependent.

Five calibrations for the Ti-in-quartz thermobarometer have been published, one based on quartz coexisting with Ti-bearing silicate melts^58^, two for quartz coexisting with fluids (H_2_O ± NaCl fluids) and rutile^26,27^, and the others synthesized quartz in the presence of rutile and either aqueous fluid or hydrous silicate melt^59,60^. Growth entrapment mechanisms and growth rate were considered to cause deviations in calculation results^27^. Deviations of equilibrium concentration of Ti in quartz caused by growth entrapment is 10% in the worst-case scenario^61^. Osborne et al.^60^, however, found that concentrations of Ti in individual quartz crystals from each experiment do not show evidence for growth rate-related phenomena. Given that the calibrations of Wark and Watson^59^ and Thomas et al.^26^ were derived from high pressure data, we applied the Zhang et al.^58^ calibration to magmatic quartz and the latest calibration (by Osborne et al.^60^) to hydrothermal quartz. Application of the Ti-in-quartz thermobarometer requires two conditions to be met: (1) the activity of rutile (a_TiO2_) in the system needs to be constrained; and (2) either T or P needs to be constrained. The solubility of rutile is low in aqueous fluids, for instance, the calculated Ti solubility in H_2_O is ≤ 15 ppm at 800 °C, 1 kbar^62^. Thus, a_TiO2_ can be assumed as 1 for hydrothermal veins in the present study. In contrast for rutile undersaturated quartz porphyry, $\rm C_{\rm Ti}^{\rm Qz}$ (Ti concentrations of quartz), $\rm C_{\rm Ti}^{\rm liq}$ (Ti concentrations of coexisting silicate melt), and compositional parameters of the melt (FM values^63^) must be known, pressure or T can be constrained^58^. The estimated formation pressure for quartz in breccia pipe, and quartz veins from microthermometric data of fluid inclusions based on the PVTX properties of H_2_O–NaCl^64^ was around 0.1 kbar. We therefore assume a pressure of 0.1 kbar for the breccia pipe, the quartz veins, and the quartz crystals in vugs, and estimate the pressure of the quartz porphyry using the solidus curve of water-saturated granite^65^ (ESM Fig. 5).

For $\rm C_{\rm Ti}^{\rm Qz}$ = 2.81 ppm (average value),  $\rm C_{\rm Ti}^{\rm liq}$ = 20.6 ppm, FM = 1.526^4^, and assuming water-saturated at the granite solidus conditions^65^, crystallization conditions for the quartz in the porphyry can be constrained at around 700 °C for P = 1.5 kbar (ESM Fig. 5). For the breccia pipe and the quartz veins, Ti-in-quartz thermometry gives 302–345 °C and 270–310 °C, respectively.

### Zoning in quartz: multi-episodic fluid and implications for sulfide precipitation

Quartz crystals from the vug in Weilasituo Sn deposit display a distinct CL response and chemical zoning (Fig. 5f, g) reflecting the involvement of at least three different fluids during crystal growth and, implicitly, during deposit formation. Each episode begins with early CL-bright quartz and ends with CL-dark quartz. Solid inclusions in quartz are cassiterite for the first episode (Ep 1), and sphalerite + chalcopyrite + arsenopyrite for the second and third episodes (Ep 2, Ep 3). Sulfides are mostly observed within CL-dark quartz (Fig. 5f, g).

Trace element contents in quartz crystals (from vug) display a continuous transition from magmatic signatures in the core (Ep 1_core_) to hydrothermal signatures in the rim (Ep 1_rim_) (Fig. 11b, c). This implies that early fluids which separated from the magma inherited the characteristics of the quartz porphyry. The Ep 1 fluid is rich in Sn but depleted in Cu and Zn. Ep 2 and Ep 3 fluids are rich in Zn (Sn) and Cu. The variation characteristics of element contents in Ep 2 fluid are consistent with those in Ep 1, for instance, the contents of Al and Li decrease from Ep 1_core_ to Ep 1_rim_ and correspondingly from Ep 2_core_ to Ep 2_rim_, although at a slightly lower overall level in the later fluids (Fig. 7). Sulfides only appear in the late stages of fluid evolution and are concentrated within CL-dark quartz (Fig. 5f, g).

Three possible processes may cause a decrease of Al and Li concentrations in quartz from core to rim. First, with ongoing zinnwaldite precipitation, Li and Al concentrations in the fluid decrease. A second alternative involves temperature decrease. However, crystallization temperatures of quartz show broadly overlapping ranges with 251–298 °C (Ep 1_core_), 261–290 °C (Ep 1_rim_), 253–289 °C (Ep 2_core_), and 248–290 °C (Ep 2_rim_)^60^. Thus, a decrease of temperature is unlikely to apply here. Thirdly, the addition of heated external fluid or CH_4_-rich fluid from the Linxi Formation would dilute the Al and Li concentrations, resulting in transformation from a magmatic to a hydrothermal fluid (Fig. 11b, c), and provide CH_4_ and possibly also Fe, Zn. Furthermore, SO_2_ produced by magma degassing (ESM Fig. 4) reacted with heated water to form SO_4_^2−^, which was reduced by CH_4_ to S^2−^^37^. Thus, addition of a reducing fluid promotes precipitation of chalcopyrite and sphalerite and inhibits precipitation of anhydrite and magnetite. On the larger scale, i.e. the Weilasituo Cu–Zn and Bairendaba Ag-Pb–Zn deposits (Fig. 1c; Table 1), addition of externally sourced, CH_4_-rich fluids may have played an important role in formation of these deposits.

### Metallogenic model for the Weilasituo district and implications

Tin-(W)-Cu-Pb-Zn-Ag mineralization in the Weilasituo district is related to emplacement of Early Cretaceous Sn-rich quartz porphyry in an extensional setting^2,3^. The quartz porphyry is highly differentiated and rich in F, Cl, S and H_2_O^4^. During magma ascent and crystallization, zinnwaldite crystallized at the top of the granite pluton, consuming Fe^2+^ and Mg^2+^ from melt, reducing Cl solubility in the melt, and promoting exsolution of HCl ± HF-bearing fluids^66,67^. Reaction between magmatic HCl ± HF-bearing aqueous fluids and feldspar resulted in greisen formation and precipitation of disseminated mineralization^68,69^.

**Figure 12:**
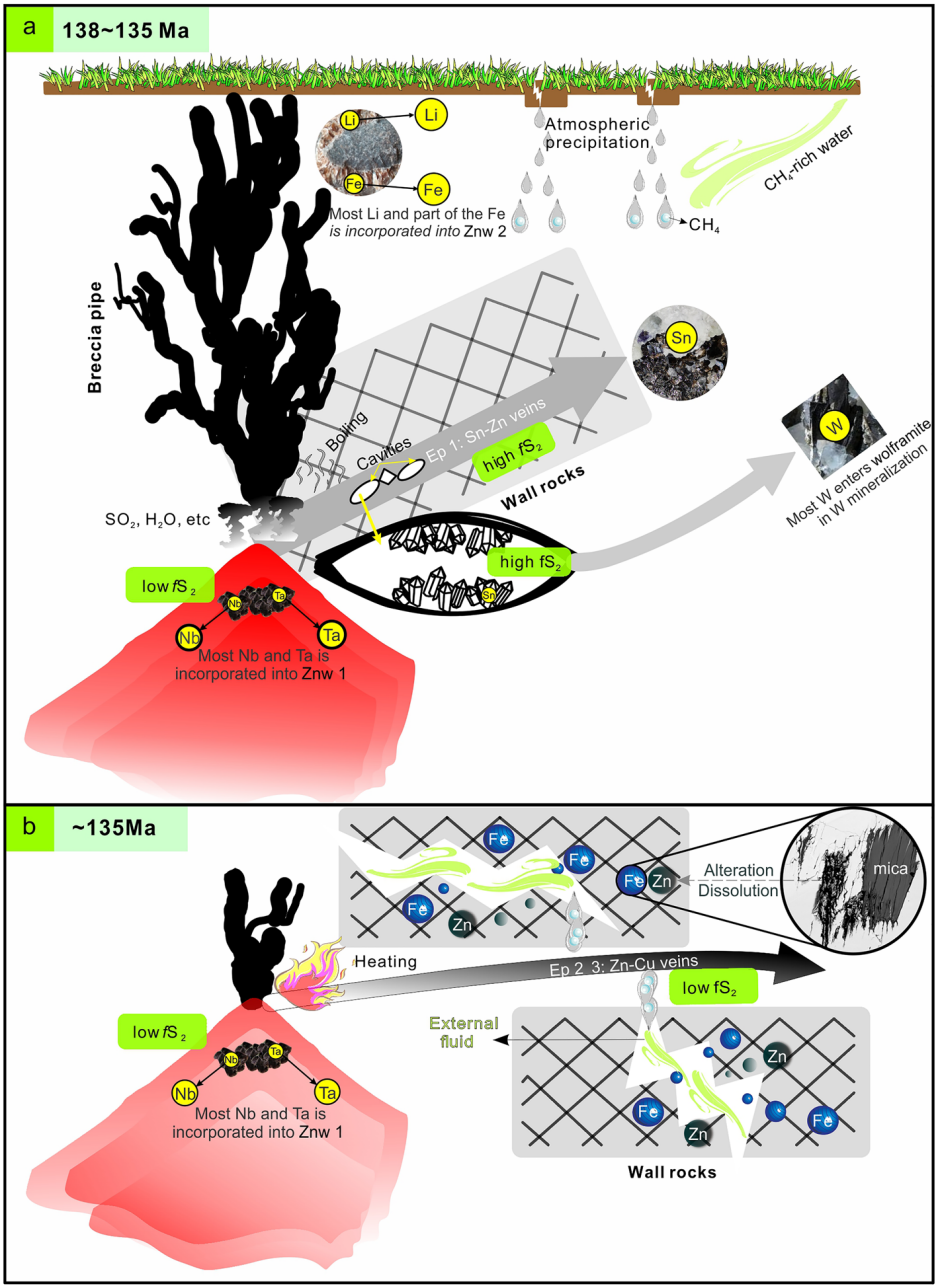
Schematic profiles illustrating the formation and metallogenic zoning in the Weilasituo district. (**a**) Intrusion and crystallization of the quartz porphyry. Separation of Sn-W-Zn rich fluid from the residual magma and explosive degassing. The SO_2_ produced by magma degassing causes a shift from low *f*S_2_ to high *f*S_2_ conditions. (**b**) Subsequent exsolution of Cu–Zn rich fluid that migrated far from the intrusion and mixed with external reduced fluids possibly derived from wall rocks, adding Fe, Zn and possibly also Cu to later stage ore-forming fluids. Znw 1: late magmatic Fe-Li mica in greisenized quartz porphyry; Znw 2: hydrothermal Fe–Li mica in a cryptoexplosive breccia pipe.

At the magmatic-hydrothermal transition stage, vapor was concentrated at the top of the porphyry intrusion, eventually resulting in the formation of a cryptoexplosive breccia pipe that was flushed by upwelling volatile-rich fluids. During this process, halogen (Cl-F)-rich fluids were likely to scavenge Sn, W, Zn, Li, and some Cu from highly evolved melts^70^. Formation of the breccia pipe resulted in a pressure drop that triggered fluid boiling, which in turn led to precipitation of ore minerals. Part of the Cu, Fe, and Zn separated into a low-density, vapor-rich fluid and migrated away from the parent fluid to form fine Cu–Zn veins in distant wall rocks. SO_2_ produced by magma degassing modified the ore-forming fluid from low *f*S_2_ to high *f*S_2_ (ESM Fig. 4)_._ Most of the Nb and Ta was incorporated into zinnwaldite^4^. Tungsten mineralization occurs in veins distal to the source of magmatic fluids, whereas Sn-Zn mineralization formed in veins proximal to the intrusion (Fig. 12a).

Fluids were exsolved from the intruding crystallizing granitic magma, leading to the formation of Ep 2 and Ep 3 fluids, which mobilized the remaining Zn, Sn and Cu from the residual melt. Copper and Zn probably migrated far from the intrusion in the form of Cl^–^, HS^–^ and SO_4_^2−^ complexes. Precipitation of chalcopyrite mainly took place via the reaction: CuCl − 2 + FeCl_2_ (aq) + H_2_S + ¼O_2_ = CuFeS_2_ + 4 Cl^−^  + 3H^+^  + ½H_2_O^71^. In the late stage, Ep 2 and Ep 3 fluids mixed with external fluid, which include meteoric water and reducing basin water from the Late Permian Linxi Formation. In addition to an increase in pH and decrease in temperature and Cl^−^ concentration, the mixing also provided Fe and Zn to the fluid by altering and dissolving mica (Fig. 4h − l) on the vein walls. Addition of Zn, Fe (an increase of FeCl_2(aq)_) and CH_4_ from external fluid facilitated reduction of SO_4_^2−^ to S^2−^, leading to precipitation of chalcopyrite and Fe-rich sphalerite, finally forming the peripheral Weilasituo Cu–Zn and Bairendaba deposits. The addition of external fluid also led to a transition from high *f*S_2_ to low *f*S_2_ fluid conditions (ESM Fig. 4 and Fig. 12b).

The SGXR has emerged as an important Sn-W-Mo-Zn-Cu-Pb-Ag belt^72–75^. Deposits such as Weilasituo, Anle, Baiyinchagan, and Bianjiadayuan, define spatial zonation with respect to metals, including Sn ± W (Mo) ± Zn mineralization proximal to, and Cu ± Pb ± Zn ± Ag mineralization distal from the intrusion. These mineralizations share similar metallogenic ages, although whether these data represent a continuous cooling of a single fluid event, or alternatively, the metal zonation pattern derived from the superposition of multi-episodic fluid processes is a question that remains controversial^2,3,76^. Our results suggest that the second alternative involving multi-episodic fluids with different ore-forming components is better suited to explain the observed metal zonation. Additional metals may have been leached from the wall rocks and contributed to late ore-forming fluid. The Linxi Formation, as an important hydrocarbon source horizon^77^, is a prominent unit of the SGXR. Our fluid inclusion analyses indicate that CH_4_-rich fluids from Linxi Formation may have played a key role in sulfide precipitation.

## Conclusions

The three deposits (Weilasituo Sn-polymetallic, Weilasituo Cu–Zn and Bairendaba Ag-Pb–Zn) are all related to emplacement of an Early Cretaceous, highly differentiated reduced quartz porphyry in plagioclase gneiss and quartz diorite. The district shows Sn (W) -Li-Zn-Cu-Pb zoning from the center of metallogenic activity to the margins. Fluid inclusion and H–O isotopic data, combined with chemical variations observed in the quartz and sphalerite from different stages of mineralization, allow us to draw the following conclusions.

1. The Weilasituo Sn-polymetallic deposit formed from low-medium temperature, low-salinity fluids. Magmatic fluids first evolved by magma degassing, fluid boiling, and eventually by addition of reducing basin water from the Late Permian Linxi Formation.
2. Magmatic quartz crystallized at around 700 °C, 1.5 kbar, and has higher contents of Al and Li, and lower Ge, Ti contents than hydrothermal quartz that crystallized at lower temperatures.
3. Sphalerite from the Weilasituo Cu–Zn and Bairendaba Ag–Pb–Zn deposits has much higher Cd, Fe + Cu, Fe/Zn, and Cd/Zn ratios than those in the Sn deposit.
4. Multi-episodic fluid processes and external fluid mixing contributed to the metal zonation in the Weilasituo and Bairendaba areas. Episode 1 fluids are characterized by high Sn-(Zn), and possibly also W concentrations, whereas fluids from episodes 2 and 3 mainly carried Zn-(Sn) together with Cu. Mixing between external fluids and episodes 2 and 3 fluids contributed to late-stage Cu, Zn mineralization, leading to precipitation of the peripheral Weilasituo Cu–Zn and Bairendaba deposits. The external fluids may have contributed to the metal endowment of these deposits.

### Supplementary Information

Supplementary Information.

## Data Availability

All data analysed during this study are included in the supplemental information files.
