# Supplementary material for: Magmatic-hydrothermal fluid evolution of the tin-polymetallic metallogenic systems from the Weilasituo ore district, Northeast China
Source: Sci Rep. 2024 Feb 6;14:3006. doi: 10.1038/s41598-024-53579-y (PMC10847423; doi:10.1038/s41598-024-53579-y)
Supplement: Supplementary file 1 — Supplementary Information. [file 41598_2024_53579_MOESM1_ESM.pdf]

Supplementary Information

Magmatic-hydrothermal fluid evolution of the tin-polymetallic metallogenic systems from the Weilasituo ore district, Northeast China

Scientific Reports

Xu Gao [1, 2], Zhenhua Zhou[1, 3*], Karel Breiter[4], Jingwen Mao[1], Rolf L. Romer[5], Nigel J. Cook[6], François Holtz[2]

[1] *MNR Key Laboratory of Metallogeny and Mineral Assessment, Institute of Mineral Resources, Chinese Academy of Geological Sciences, Beijing 100037, China*

[2] *Institut für Mineralogie, Leibniz Universität Hannover, Callinstr. 3, D-30167 Hannover, Germany*

[3] *Research School of Earth Sciences, Australian National University, Canberra, Australian Capital Territory 2601, Australia*

[4] *Institute of Geology of the Czech Academy of Sciences, Rozvojová 269, CZ-16500, Praha 6, Czech Republic*

[5] *GFZ German Research Centre for Geosciences, Telegrafenberg, D-14473 Potsdam, Germany*

[6] *School of Chemical Engineering, The University of Adelaide, SA 5005, Australia*

[*] Corresponding author (Z. H. Zhou) at: 142 Mills Rd, Research School of Earth Sciences, Australian National University, Canberra, ACT 2601, Australia.
E-mail address: zhzhoucags@sina.com

Additional information of analysed samples

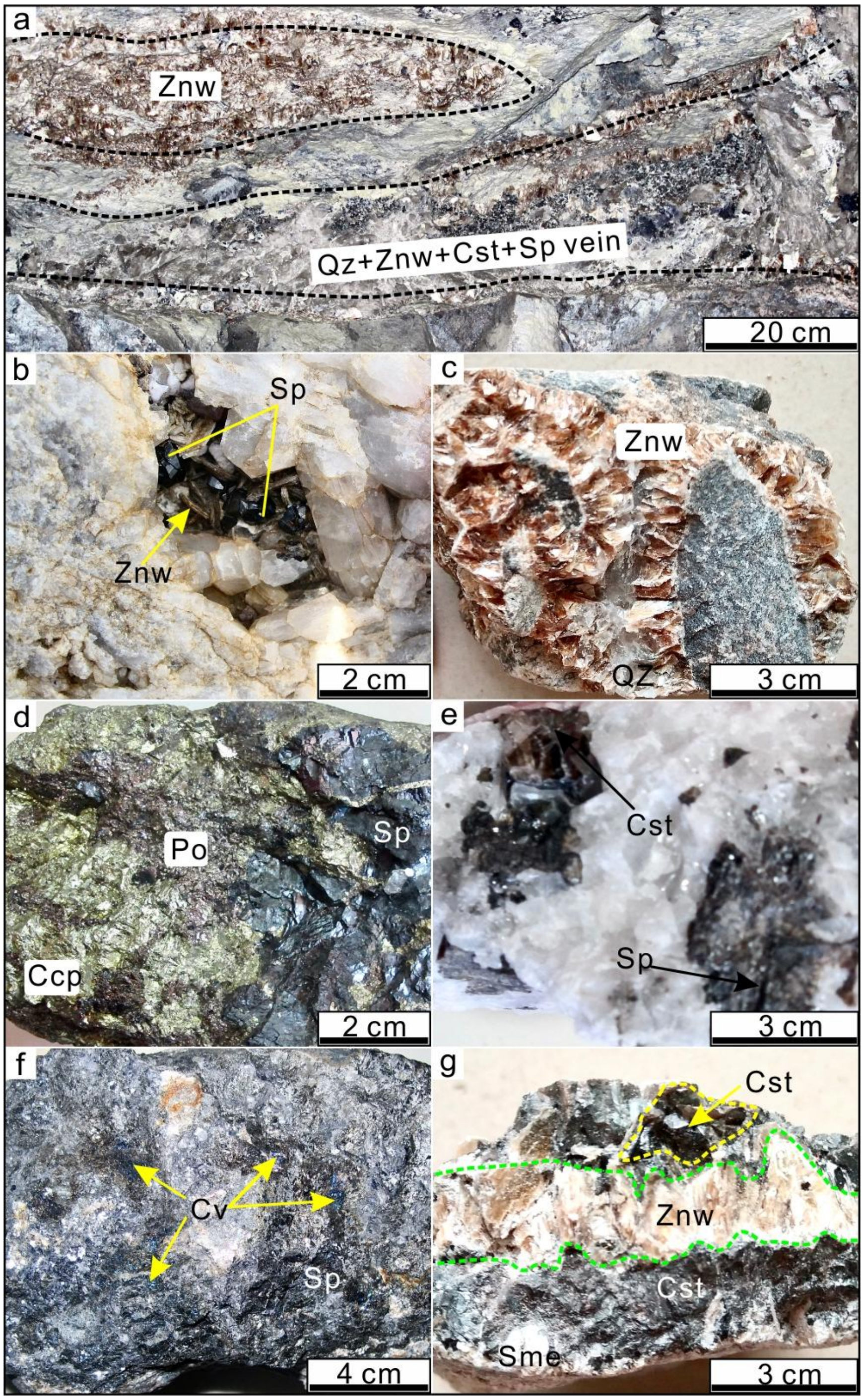

ESM Fig. 1. Minerals from the Weilasituo deposits. (a) Tunnel wall with zinnwaldite and smectite. (b) Euhedral sphalerite in a geode. (c) Mica on the surface of clasts of the cryptoexplosive breccia. (d) Sphalerite with chalcopyrite, pyrite, and pyrrhotite from the Weilasituo Cu-Zn deposit. (e) Cassiterite associated with sphalerite and quartz in the massive ore of the Weilasituo tin deposit. (f) Sphalerite with covellite from the tin-polymetallic deposit. (g) Cassiterite, zinnwaldite and sphalerite grown on surface of montmorillonite-altered wall rock. Abbreviations: *Ccp*–chalcopyrite, *Cst*–cassiterite, *Cv*–covellite, *Fl*–fluorite, *Py*–pyrite, *Po*–pyrrhotite, *Qz*–quartz, *Sp*–sphalerite, *Sme*–smectite, *Znw*–zinnwaldite.

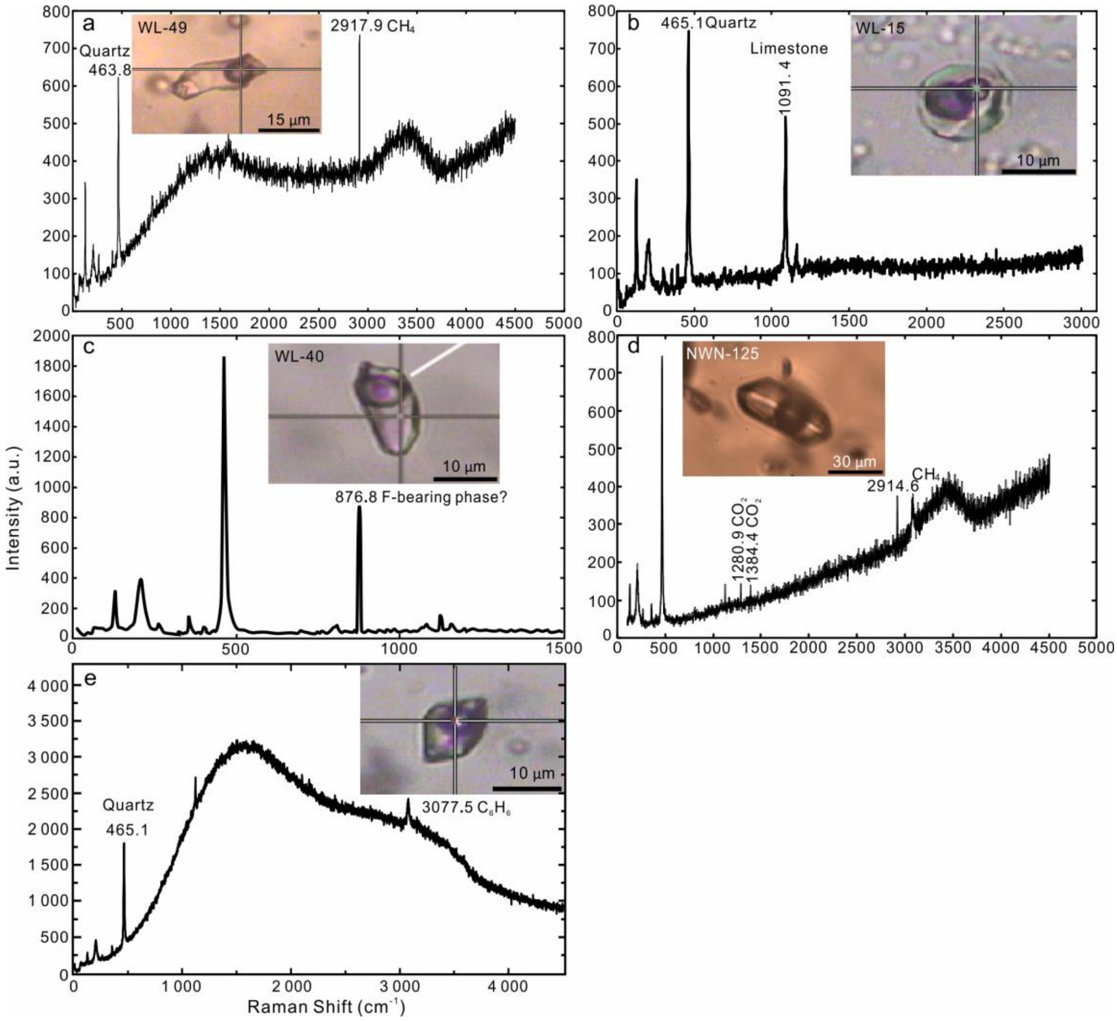

ESM Fig. 2. Raman spectroscopy of fluid inclusions in the Weilasituo tin-polymetallic deposit. (a) Fluid inclusions in Stage I quartz contain liquid (H_2O) and vapour (CH_4) bubble. (b) Stage I L-V-S inclusions with a limestone daughter crystal. (c) Aqueous L-V inclusions in Stage I quartz with liquid (H_2O) and vapour bubble. (d) Fluid inclusions in Stage III quartz with liquid (H_2O) and vapour (CH_4 and CO_2) bubble. (e) Stage III L-V inclusions with liquid (H_2O) and vapour (C_6H_6) bubble.

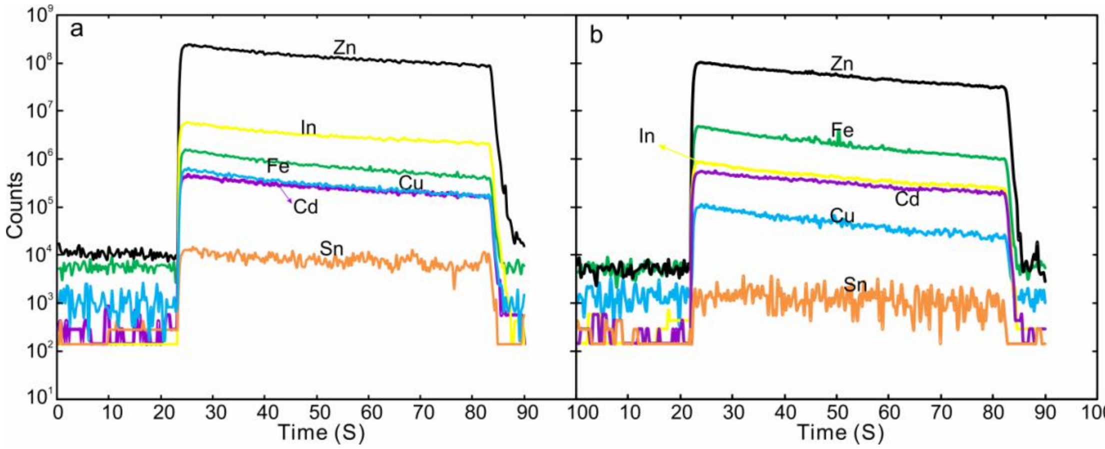

ESM Fig. 3. Representative single-spot LA-ICP-MS spectra for selected elements in sphalerite. (a) Sphalerite from a quartz vein in Weilasituo Sn deposit with In>Fe. (b) Sphalerite from a quartz vein in the Weilasituo Cu-Zn deposit with In<Fe.

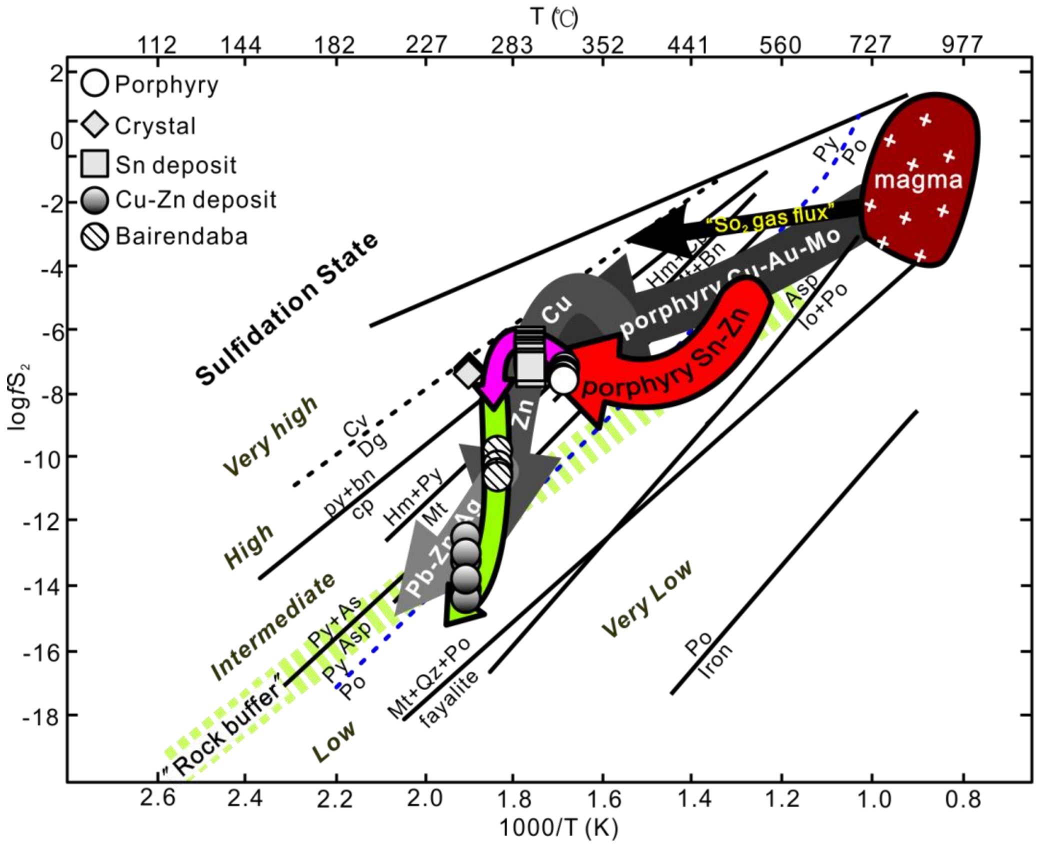

ESM Fig. 4. fS_2 versus 1000/T diagram showing the evolution of mineralizing fluids that deposited Sn and Cu-Zn-Pb-Ag mineralization in the Weilasituo-Bairendaba district[1]. The Weilasituo Sn-polymetallic deposit includes an early magma-related low sulfidation stage (red arrow, fS_2: –7.5 ~ –5.0), which is followed by a high-temperature hydrothermal stage (magenta arrow, fS_2: –6.9 ~ –6.3). Increasing sulfidation took place during cooling from near magmatic temperatures to 250~300 °C and increasing disproportionation of SO_2 via the reaction: $4SO_2(g) + 4H_2O = 4H_2SO_3(aq) = H_2S(aq) + 3\ H_2SO_4(aq)$, with H_2SO_4 dissociating to H^+ and HSO_4^-/SO_4^{2-} [1]. Addition of SO_2 from a degassing magma enhanced fluid reactivity (arrow labeled "SO_2 flux")[2,3]. In the periphery of Weilasituo tin-polymetallic deposit, i.e., in the Weilasituo Cu-Zn and Bairendaba Ag-Pb-Zn deposits, fS_2 evolved to a low sulfidation state (green arrow, fS_2: –14.3 ~ –9.7). Abbreviations: Asp–arsenopyrite; Bn–bornite; Cp–chalcopyrite; Cv–covellite; Dg–Digenite; En–Enargite; Fm–famatinite; Hm–hematite; Lo–löllingite; Mt–magnetite; Po–pyrrhotite; Py–pyrite; Qtz–quartz; vns–veins; Temperature ranges based on fluid inclusions data: “Porphyry Sn-Zn” stage (red arrow); “quartz-vein Sn-Zn” stage (magenta arrow); “quartz-vein Cu-Zn-Pb-Ag” stage (green arrow); “Porphyry Cu-Au-Mo” (black arrow) and related zoned base-metal veins (grey arrow)[1]. fS_2 was calculated using the equation $\log_{10}fS_2 = 11.01 - 9.49\ (1000/K) + [0.187 - 0.252(1000/K)]$ (mol% FeS in sphalerite), except for the last stage (quartz-vein Cu-Zn-Pb-Ag stage) that was calculated using the equation $\log_{10}fS_2 = 11.01 - 9.49\ (1000/K) + [0.187 - 0.252(1000/K)]$ (mol% FeS in sphalerite) + $[0.35 - 0.2\ (1000/K)]$ (mol% CuS in sphalerite) [4].

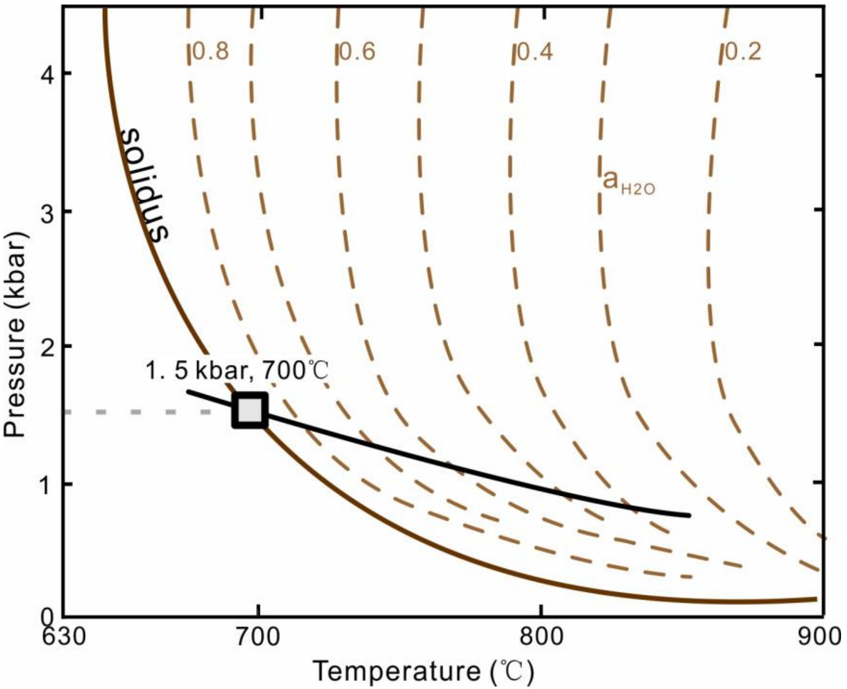

ESM Fig. 5. Pressure vs. temperature diagram showing quartz crystallization conditions at the top of the porphyry. The solid curve represents the solidus of water-saturated granite system, the black line represents pressure and temperature conditions at which C_{Ti}^{Qtz} = 2.81 ppm and C_{Ti}^{liq} = 20.6 ppm for a melt composition with FM = 1.526.

Analytical methods

Fluid inclusions and Laser Raman study

Fluid inclusion microthermometry was carried out at the Institute of Mineral Resources, Chinese Academy of Geological Sciences, Beijing (IMR-CAGS) using Linkam THMSG600 and THMSG1500 freeze-heating systems. The morphology, size, and phase change of the inclusions were observed using a Zeiss microscope. The gas-flow heating/freezing system was calibrated at –56.6, 0.0 and 374.1 °C using synthetic fluid inclusions produced by FLUIDINC (USA). Homogenization behavior and the phase change at the critical point was observed at a rate of 0.5 to 1 °C/min. The composition of individual inclusions was determined by Laser Raman spectroscopic analysis at IMR-CAGS, using a Renishaw System-2000 Viaconfocal Raman spectrometer, using a 514 nm argon laser as light source and a 1 μm-diameter laser spot. Laser power was 20 mW, scanning range was 100–4500 cm^{-1} and the spectral resolution was 1–2 cm^{-1}.

Hydrogen and oxygen isotope analysis

Hydrogen and oxygen isotopic compositions were determined at IMR-CAGS, using a MAT 253 EM mass spectrometer. Analytical precision of oxygen and hydrogen isotope data is ±0.2 ‰ and ±2 ‰, respectively. For hydrogen isotope analysis, quartz was heated to burst the fluid inclusions. Water vapor was extracted from the released volatiles. Hydrogen was released by reacting of water with zinc at 400 °C and collected on activated carbon[5]. For the oxygen isotope analysis, quartz was reacted with BrF_5 and C to produce CO_2 that was extracted[6]. The oxygen isotopic composition of water in quartz was calculated according to the fractionation equation $1000 \ln \alpha_{quartz\ water} = 3.38 \times 10^6 / T^2 – 3.40$[7].

Analysis of trace elements in sphalerite

Major and trace element analyses were conducted by LA-ICP-MS at the State Key Laboratory of Ore Deposit Geochemistry, Institute of Geochemistry Chinese Academy of Sciences (IGCAS). Laser sampling was performed using an ASI RESOLution-LR-S155 laser microprobe equipped with a Coherent Compex-Pro 193 nm ArF excimer laser. An Agilent 7700x ICP-MS instrument was used to acquire ion-signal intensities. Helium (350 ml/min) was used as a carrier gas that was mixed with Argon (900 ml/min) via a T-connector before entering the ICP-MS. Each analysis included 30 s background acquisition (gas blank) and 60 s of data acquisition from the sample. The laser was operated at a 5 Hz pulse frequency, 3 J/cm^2 fluence, and 26 μm-diameter spot size.

GSE–1G, GSD–1G, and FeSb were used as external standards and analyzed twice after each set of 10 analyses. Among them, FeSb was used to determine concentrations of chalcophile and siderophile elements, GSE–1G and GSD–1G were used to calibrate the concentrations of lithophile elements[8]. The sulfide reference material MASS–1 was analyzed as an unknown to check analytical accuracy of the results. Uncertainties were determined to be <5% for major elements and <10% for trace elements (except for Co, Ni, Sb).

Cathodoluminescence and analysis of trace elements in quartz

Hyperspectral CL images were obtained using a TESCAN field emission scanning electron microscope (model MIRA 3LMH) with a CL detector system at Nanjing Hongchuang Exploration Technology Service Co., Ltd. Acceleration voltage and beam current were set to 15-20 kV and 1.2 nA, respectively, with 80 s scan time. Quantitative analysis of trace elements in quartz was conducted at the State Key Laboratory of Ore Deposit Geochemistry, IGCAS, using an Agilent 7900 ICP-MS

equipped with a GeoLasPro 193 nm ArF excimer laser. Helium was used as a carrier gas and was mixed with Argon via a T-connector before entering the ICP-MS. During analysis, the laser was operated with a repetition rate of 10 Hz, an energy density of 12 J/cm^2, and a spot size of 44 μm. Before analysis, the performance of the ICP-MS was optimized using SRM610 to achieve best sensitivity and ionization efficiency (U/Th≈1), minimum oxide yield (ThO/Th<0.3%), and a low background value. The content was calculated using multiple external standards and the total normalization method[9]. NIST SRM610 was used as external standard and analyzed twice after each set of 10 analyses. The natural quartz standards (QZ) and NIST SRM612 were analyzed to monitor the accuracy of the results that have uncertainties <10% for most elements.

References

1. Einaudi, M.T., Hedenquist, J.W. & Inan, E. Sulfidation state of hydrothermal fluids: the porphyry-epithermal transition and beyond. In: Simmons SF, Graham I (ed) Volcanic, geothermal and ore-forming fluids: rulers and witnesses of processes within the Earth. Soc Econ Geol and Geochem Soc Spec Publ.**10**, 285–313 (2003).

2. Brimhall, G.H. Jr. Lithologic determination of mass transfer mechanisms of multiple-stage porphyry copper mineralization at Butte, Montana: Vein formation by hypogene leaching and enrichment of potassiumsilicate protore. Econ Geol. **74**, 558–589 (1979).

3. Brimhall, G.H. Jr. Deep hypogene oxidation of porphyry copper potassium–silicate protore at Butte, Montana: A theoretical evaluation of the copper remobilization hypothesis. Econ Geol. **75**, 384–409 (1980).

4. Lusk, J. & Calder, B.O.E. The composition of sphalerite and associated sulfides in reactions of the Cu-Fe-Zn-S, Fe-Zn-S and Cu-Fe-S systems at 1 bar and temperatures between 250 and 535 °C. Chem Geol. **203**, 319–345 (2004).

5. Coleman, M.L., Shepherd, T.J., Durham, J.J., John, E.R. & Gillian, R. Reduction of water with zinc for hydrogen isotope analysis. Anal Chem. **40**, 993–995 (1982).

6. Clayton, R.N. & Mayeda, T.K. The use of bromine pentafluoride in the extraction of oxygen from oxides and silicates for isotopic analysis. Geochim Cosmochim Acta. **40**, 43–52 (1963).

7. Clayton, R.N., O' Neil, J.R. & Mayeda, T.K. Oxygen isotope exchange between quartz and water. J Geophys Res. **40**, 3057–3067 (1972).

8. Danyushevsky, L., Robinson, P., Gilbert, S., Norman, M., Large, R., McGoldrick, P. & Shelley, M. Routine quantitative multi-element analysis of sulphide minerals by laser ablation ICP-MS: Standard development and consideration of matrix effects. GEOCHEM-EXPLOR ENV A. **11**, 51–60 (2011).

9. Liu, Y.S., Hu, Z.C., Gao, S., Günther, D., Xu, J., Gao, C.G. & Chen, H.H. In situ analysis of major and trace elements of anhydrous minerals by LA-ICP-MS without applying an internal standard. Chem Geol. **40**, 34–43 (2008).

ESM Table 1. Sample description

Sample no.	Lithology	Sampling location	Sample description	Main Experiments
**NW-19**	Quartz vein-type ore	Ore Dump of No. 4 well in Copper-zinc deposit; 44°4′16″ N, 117°29′15″ E	massive Sphalerite	LA-ICP-MC analysis of sphalerite
**NW-4-7**	Quartz vein-type ore	Ore Dump of No. 4 well in Copper-zinc deposit; 44°4′16″ N, 117°29′15″ E	massive Sphalerite	LA-ICP-MC analysis of sphalerite
**NWN-3**	Quartz vein-type ore	South inclined shaft; 44°5′39″ N, 117°27′56″ E	sphalerite with arsenopyrite and wolframite	LA-ICP-MC analysis of sphalerite
**NWN-29**	Quartz vein-type ore	South inclined shaft; 44°5′39″ N, 117°27′56″ E E	sphalerite with arsenopyrite and wolframite	LA-ICP-MC analysis of sphalerite
**WL-6-3**	Quartz vein-type ore	Ore Dump of No. 6 well in tin deposit	Quartz-vein-type sphalerite	LA-ICP-MC analysis of sphalerite
**WL-6-13**	Quartz vein-type ore	Ore Dump of No. 6 well in tin deposit	Quartz-vein-type sphalerite with a little amount of molybdenite	LA-ICP-MC analysis of sphalerite
**WL-6**	Quartz vein-type ore	ZK02410 155~158m	sphalerite with arsenopyrite	LA-ICP-MC analysis of sphalerite
**WL-Y-11**	Quartz porphyry	ZK01505 467-470m	Qz(65%), Ab (15%), Znw (5%), K-feldspar (10%), with some disseminated sphalerite	LA-ICP-MC analysis of sphalerite
**WL-Y-4**	Quartz porphyry	ZK01505 523-526m	Qz(60%), Ab (25%), K-feldspar (10%), Znw(5%), with some disseminated sphalerite	LA-ICP-MC analysis of sphalerite
**WQ-6**	Quartz crystal	No.3 middle segment,South inclined shaft	dark sulfides in the crystal	LA-ICP-MC analysis of sphalerite

**WB-91**	massive sulfide ore	Ore Dump of No. 1 well in Bairendaba deposit	mainly sphalerite, containing a little amount of chalcopyrite and pyrrhotite	LA-ICP-MC analysis of sphalerite
**WB-92**	massive sulfide ore	Ore Dump of No. 1 well in Bairendaba deposit	mainly sphalerite with some pyrrhotite	LA-ICP-MC analysis of sphalerite
**WB-97**	massive sulfide ore	Ore Dump of No. 1 well in Bairendaba deposit	mainly sphalerite with some pyrrhotite	LA-ICP-MC analysis of sphalerite
**WL-45**	Quartz vein-type ore	ZK03211 377~380m	massive sphalerite	LA-ICP-MC analysis of Qz and H-O isotopes
**NW-Y-127**	Breccia pipe	ZK2305, 207m	a large amount of zinnwaldite on the surface of clasts	LA-ICP-MC analysis of Qz
**WL-47**	Quartz porphyry	ZK02306 482~485m	quartz porphyry with some disseminated sphalerite	LA-ICP-MC analysis of Qz
**WQ-5**	Quartz crystal	No.3 middle segment, South inclined shaft	dark sulfides and some cassiterite	LA-ICP-MC analysis of Qz
**WL-13**	Quartz porphyry	ZK02408 323~326m	Qz(60%), Ab (20%), Znw (10%), K-feldspar (5%). Albite phenocryst, matrix quartz	Thermometry of fluid inclusions
**WL-15**	Quartz porphyry	ZK02408 323~326m	Qz(60%), Ab (20%), K-feldspar (10%), Znw (5%). Albite phenocryst, matrix quartz	Thermometry of fluid inclusions and H-O isotopes
**WL-16**	Quartz porphyry	ZK02408 323~326m	Qz(60%), Znw (10%), Ab (15%), K-feldspar (10%), also contains a little amount of zircon	Thermometry of fluid inclusions and H-O isotopes
**WL-18**	Quartz vein-type ore	ZK03205 406-409m	massive arsenopyrite	H-O isotopes
**WL-19**	Quartz vein-type ore	ZK03205 406-409m	massive sphalerite and arsenopyrite, with vugs in quartz vein	H-O isotopes
**WL-20**	Quartz vein-type ore	ZK03205 406-409m	massive arsenopyrite, with vugs in quartz vein	H-O isotopes

**WL-21**	Quartz vein-type ore	ZK03205 406-409m	massive wolframite and arsenopyrite	Thermometry of fluid inclusions and H-O isotopes
**WL-24**	Quartz vein-type ore	ZK01506 415-418m	mainly arsenopyrite, with a little amount of sphalerite	Thermometry of fluid inclusions and H-O isotopes
**WL-27**	Quartz porphyry	ZK1610 352-355m	Qz(65%), Ab (15%), K-feldspar (5%), Znw (10%), with snowball-structured quartz	Thermometry of fluid inclusions
**WL-28**	Quartz vein-type ore	ZK0002 151-154m	Mainly sphalerite, with a little amount of cassiterite	H-O isotopes
**WL-40**	Quartz porphyry	ZK0705 544-547m	Qz (65%), Ab (20%), K-feldspar (10%), Znw (5%), with strong albitization	Thermometry of fluid inclusions
**WL-41**	Quartz vein-type ore	ZK0705 478-481m	massive sphalerite with some cassiterite	Thermometry of fluid inclusions and H-O isotopes
**WL-42**	Quartz vein-type ore	ZK0705 478-481m	massive sphalerite with some cassiterite	Thermometry of fluid inclusions and H-O isotopes
**WL-43**	Quartz vein-type ore	ZK03102 256-259m	massive sphalerite and arsenopyrite	H-O isotopes
**WL-44**	Quartz vein-type ore	ZK0808 183-186m	containing a little amount of sphalerite	H-O isotopes
**WL-46**	Quartz vein-type ore	ZK03211 377-380m	massive sphalerite and löllingite	Thermometry of fluid inclusions

**WL-49**	Albitization quartz porphyry	ZK02306 482-485m	Qz (60%), Ab (20%), K-feldspar (5%), Znw (10%), with snowball-structured quartz and a little amount of sphalerite	Thermometry of fluid inclusions
**WL-52**	Quartz vein-type ore	ZK03211 377-380m	massive sphalerite and some arsenopyrite	H-O isotopes
**NW-Y-12**	Quartz vein-type ore	Ore dump near South inclined shaft	massive sphalerite and arsenopyrite	Thermometry of fluid inclusions
**NW-Y-95**	Quartz porphyry	IZK02305 622-625m	Qz(65%), Ab (15%), K-feldspar (5%), Znw (10%), with snowball-structured quartz	Thermometry of fluid inclusions
**NWN-121**	Quartz vein-type ore	South inclined shaft; 44°5′39″ N, 117°27′56″ E	massive sphalerite	Thermometry of fluid inclusions
**NWN-123**	Quartz vein-type ore	South inclined shaft; 44°5′32″ N, 117°27′59″ E	Quartz-vein-type cassiterite with sphalerite	Thermometry of fluid inclusions
**NWN-125**	Quartz vein-type ore	South inclined shaft; 44°5′32″ N, 117°27′59″ E	Quartz-vein-type cassiterite	Thermometry of fluid inclusions
**NWN-127**	Quartz vein-type ore	South inclined shaft; 44°5′32″ N, 117°27′59″ E	Quartz-vein-type sphalerite and löllingite	Thermometry of fluid inclusions

ESM Table 2. H and O isotope data for the various mineralization stages

Sample	Stage	mineral	$\delta D_{v\text{-}SMOW}$ ‰	$\delta^{18}O_{Q(v\text{-}SMOW)}$ ‰	$\delta^{18}O_{H2O(v\text{-}SMOW)}$ ‰	Temperature/°C
WL-15	I	Quartz	-67	12.6	5.5	295
WL-16	I	Quartz		12.8	6.2	309
WL-18	III	Quartz	-109	11.7	3.8	273
WL-19	III	Quartz	-86	11.3	3.6	278
WL-20	III	Quartz	-87	11.5	3.8	278
WL-21	III	Quartz	-93	11.2	2.8	263
WL-28	III	Quartz	-79	11.2	4.0	292
WL-41	III	Quartz	-79	9.7	3.3	315
WL-42	III	Quartz	-86	9.1	2.3	302
WL-45	IV	Quartz	-128	11.3	1.0	224
WL-46	IV	Quartz	-136	11.8	2.9	250
WL-52	IV	Quartz	-128	12.0	2.8	244
WL-24	IV	Quartz	-93	11.4	0.3	210
WL-43	IV	Quartz	-121	11.6	2.7	251
WL-44	IV	Quartz	-134	11.5	1.1	222

ESM Table 3. LA–ICP–MS analytical results for sphalerite from the Weilasituo and Bairendaba deposits

1. Weilasituo deposit

Measured concentrations

	Sample	Mn	Fe	Cu	Zn	Cd	Ga	Ge	As	Se	Mo	Pd	Ag	In	Sn	Sb
		wt.%	wt.%	wt.%	wt.%	wt.%	ppm	ppm	ppm	ppm	ppm	ppm	ppm	ppm	ppm	ppm
Quartz vein;Cu-Zn deposit	NW-19-SP.44	0.08	10.5	0.12	55.9	0.30	2.95	2.04	12.9	1.86	0.07	142	30.7	211	3.43	4.68
Quartz vein;Cu-Zn deposit	NW-19-SP.45	0.07	10.5	0.14	55.9	0.32	2.16	2.23	0.50	2.32	b.d.l.	152	33.4	217	16.7	4.48
Quartz vein;Cu-Zn deposit	NW-19-SP.46	0.08	11.6	0.16	54.7	0.33	3.33	2.07	1.33	1.54	b.d.l.	156	23.3	218	6.40	0.44
Quartz vein;Cu-Zn deposit	NW-19-SP.47	0.09	11.5	0.03	55.0	0.31	1.96	1.99	4.16	1.89	0.06	146	16.1	212	3.66	1.78
Quartz vein;Cu-Zn deposit	NW-19-SP.48	0.09	11.5	1.36	53.6	0.31	2.71	1.89	22.8	1.97	0.13	146	57.7	239	26.8	8.00
Quartz vein;Cu-Zn deposit	NW-19-SP.49	0.09	12.1	1.55	52.8	0.28	2.35	2.13	61.1	3.46	0.15	133	53.5	222	44.2	5.42
Quartz vein;Cu-Zn deposit	NW-4-7-SP.37	0.06	11.8	1.87	52.7	0.38	1.83	5.18	b.d.l.	b.d.l.	b.d.l.	139	59.9	202	108	12.9
Quartz vein;Cu-Zn deposit	NW-4-7-SP.38	0.09	13.8	0.03	52.5	0.40	2.92	2.39	b.d.l.	16.4	b.d.l.	146	10.6	273	8.33	b.d.l.
Quartz vein;Cu-Zn deposit	NW-4-7-SP.39	0.09	13.5	0.05	52.8	0.38	2.51	1.98	b.d.l.	14.8	b.d.l.	135	10.8	279	2.55	0.19
Quartz vein;Cu-Zn deposit	NW-4-7-SP.40	0.07	11.5	0.03	54.9	0.40	2.30	2.00	b.d.l.	13.1	b.d.l.	144	12.0	265	13.4	1.49
Quartz vein;Cu-Zn deposit	NW-4-7-SP.41	0.10	14.4	0.03	51.9	0.39	2.61	2.08	b.d.l.	12.8	b.d.l.	139	8.72	288	2.52	0.18
Quartz vein;Cu-Zn deposit	NW-4-7-SP.42	0.10	13.1	0.02	53.2	0.41	2.38	1.98	b.d.l.	10.4	b.d.l.	147	8.71	293	3.14	b.d.l.
Quartz vein;Sn deposit	NWN-3-SP.58	0.04	2.32	0.08	64.5	0.12	2.97	1.03	b.d.l.	36.1	b.d.l.	61.2	4.11	1060	4.96	0.01
Quartz vein;Sn deposit	NWN-3-SP.59	0.05	2.34	0.08	64.5	0.12	2.95	0.97	0.16	35.0	b.d.l.	62.2	3.86	1080	4.03	0.05
Quartz vein;Sn deposit	NWN-3-SP.60	0.04	2.25	0.08	64.6	0.12	2.82	0.97	0.18	37.7	b.d.l.	62.3	6.15	1010	8.55	0.87
Quartz vein;Sn deposit	NWN-3-SP.61	0.04	2.26	0.08	64.6	0.12	2.64	0.9	b.d.l.	41.1	b.d.l.	62.3	4.42	996	5.18	0.01
Quartz vein;Sn deposit	NWN-3-SP.62	0.04	2.33	0.08	64.5	0.12	2.48	0.85	b.d.l.	40.4	b.d.l.	62.4	4.16	1060	4.52	0.02
Quartz vein;Sn deposit	NWN-3-SP.63	0.05	2.38	0.08	64.4	0.12	2.66	0.89	b.d.l.	39.2	b.d.l.	62.5	3.6	1090	3.31	b.d.l.
Quartz vein;Sn deposit	NWN-3-SP.64	0.05	2.37	0.08	64.4	0.12	2.67	0.92	b.d.l.	42.2	b.d.l.	62.7	4.14	1070	3.37	0.02
Quartz vein;Sn deposit	NWN-3-SP.65	0.04	2.34	0.08	64.5	0.12	2.54	0.96	b.d.l.	40.0	b.d.l.	63.3	6.23	1030	8.33	0.02
Quartz vein;Sn deposit	NWN-29-SP.50	0.05	2.46	0.07	64.4	0.11	5.03	0.96	b.d.l.	65.4	b.d.l.	48.2	10.9	778	9.04	0.46

Quartz vein;Sn deposit	NWN-29-SP.51	0.06	2.50	0.07	64.3	0.12	3.64	0.96	b.d.l.	69.5	b.d.l.	49.3	9.04	802	35.8	b.d.l.
Quartz vein;Sn deposit	NWN-29-SP.52	0.05	2.44	0.07	64.4	0.11	4.39	0.79	b.d.l.	40.7	b.d.l.	49.5	8.81	781	17.2	b.d.l.
Quartz vein;Sn deposit	NWN-29-SP.53	0.06	2.49	0.07	64.3	0.12	3.68	0.57	1.11	62.7	1.66	48.4	9.32	809	39.3	1.45
Quartz vein;Sn deposit	NWN-29-SP.54	0.04	2.41	0.07	64.4	0.12	6.86	0.84	0.33	51.1	b.d.l.	49.1	30.2	783	58.0	3.46
Quartz vein;Sn deposit	NWN-29-SP.55	0.04	2.37	0.07	64.4	0.12	3.91	0.86	b.d.l.	61.1	b.d.l.	49.6	6.48	785	55.3	b.d.l.
Quartz vein;Sn deposit	NWN-29-SP.56	0.06	2.48	0.07	64.3	0.12	4.51	0.65	b.d.l.	59.7	b.d.l.	49.7	6.95	822	38.3	b.d.l.
Quartz vein;Sn deposit	NWN-29-SP.57	0.03	2.11	0.07	64.7	0.12	8.67	0.60	b.d.l.	60.1	b.d.l.	49.8	24.1	852	10.1	0.33
Quartz vein;Sn deposit	WL-6-13-SP.66	0.05	2.37	0.07	64.4	0.15	3.92	1.26	5.22	98.7	1.17	60.3	2.44	811	26.1	0.37
Quartz vein;Sn deposit	WL-6-13-SP.67	0.05	2.22	0.07	64.6	0.14	4.08	0.95	1.62	92.0	b.d.l.	58.4	2.32	805	33.4	b.d.l.
Quartz vein;Sn deposit	WL-6-13-SP.68	0.05	2.23	0.07	64.6	0.15	4.21	0.93	0.26	81.8	b.d.l.	61.5	2.29	749	34.4	b.d.l.
Quartz vein;Sn deposit	WL-6-13-SP.69	0.05	2.20	0.05	64.6	0.15	5.75	0.83	0.41	73.6	b.d.l.	61.5	2.40	630	14.3	0.15
Quartz vein;Sn deposit	WL-6-13-SP.70	0.05	2.24	0.07	64.6	0.15	3.96	0.61	0.67	95.7	b.d.l.	61.7	2.03	778	18.7	0.12
Quartz vein;Sn deposit	WL-6-13-SP.71	0.05	2.25	0.07	64.6	0.15	4.34	0.83	b.d.l.	95.9	b.d.l.	61.8	2.18	769	30.1	0.10
Quartz vein;Sn deposit	WL-6-13-SP.72	0.04	2.24	0.05	64.6	0.15	5.47	0.84	b.d.l.	72.8	b.d.l.	61.9	9.02	631	6.15	1.13
Quartz vein;Sn deposit	WL-6-3-SP.78	0.04	1.86	0.03	65.0	0.13	10.7	0.71	0.82	21.5	b.d.l.	53.8	356	396	19.8	31.4
Quartz vein;Sn deposit	WL-6-3-SP.79	0.04	1.89	0.03	65.0	0.13	12.8	1.01	b.d.l.	7.52	b.d.l.	55.3	104	389	6.72	2.76
Quartz vein;Sn deposit	WL-6-3-SP.80	0.04	1.88	0.03	65.0	0.13	12.8	0.70	1.90	8.88	b.d.l.	55.8	261	398	20.3	37.9
Quartz vein;Sn deposit	WL-6-3-SP.81	0.04	1.94	0.04	64.9	0.14	13.4	0.66	b.d.l.	4.79	b.d.l.	55.9	41.2	401	51.9	4.74
Quartz vein;Sn deposit	WL-6-3-SP.82	0.04	1.97	0.04	64.9	0.13	13.2	0.74	0.63	9.70	b.d.l.	54.9	186	406	39.7	15.1
Quartz vein;Sn deposit	WL-6-3-SP.83	0.04	1.98	0.03	64.9	0.13	13.6	0.52	0.57	5.36	b.d.l.	57.0	98.7	402	12.4	14.5
Quartz vein;Sn deposit	WL-6(2)-SP.84	0.04	1.21	0.06	65.7	0.14	4.12	0.44	22.8	36.7	b.d.l.	57.6	8.19	675	55.0	0.32
Quartz vein;Sn deposit	WL-6(2)-SP.85	0.04	1.21	0.06	65.7	0.14	5.71	0.48	10.7	26.2	b.d.l.	55.9	4.46	666	11.2	0.14
Quartz vein;Sn deposit	WL-6(2)-SP.86	0.04	1.29	0.07	65.6	0.14	3.96	0.44	93.1	46.0	0.11	58.0	10.2	699	74.0	7.30
Quartz vein;Sn deposit	WL-6(2)-SP.87	0.04	1.14	0.07	65.7	0.14	3.64	0.72	4.74	53.3	b.d.l.	57.6	12.2	701	60.8	4.77
Quartz vein;Sn deposit	WL-6(2)-SP.88	0.04	1.22	0.07	65.7	0.14	5.32	0.59	1.70	57.3	0.09	59.2	5.58	721	71.9	0.61
from porphyry	WL-Y-11(17)-SP.89	0.16	5.20	0.02	61.5	0.10	17.3	1.37	0.69	60.5	0.12	41.6	5.45	211	10.0	0.22
from porphyry	WL-Y-11(17)-SP.90	0.15	5.22	0.02	61.5	0.10	19.3	1.35	0.68	63.1	0.11	40.7	5.46	214	18.0	0.14
from porphyry	WL-Y-11(17)-SP.91	0.15	5.20	0.02	61.5	0.10	18.4	1.40	0.66	36.5	0.55	41.3	3.62	211	26.0	0.21
from porphyry	WL-Y-11(17)-SP.92	0.16	5.46	0.02	61.3	0.10	17.6	1.49	b.d.l.	48.4	0.54	43.8	3.32	220	27.4	0.27

from porphyry	WL-Y-11(17)-SP.93	0.15	5.30	0.02	61.4	0.10	7.54	1.46	b.d.l.	89.1	b.d.l.	42.2	3.65	213	32.2	b.d.l.
from porphyry	WL-Y-11(17)-SP.94	0.16	5.24	0.02	61.5	0.10	16.4	1.17	0.35	66.3	b.d.l.	41.6	4.83	215	19.9	b.d.l.
from porphyry	WL-Y-11(17)-SP.95	0.15	5.29	0.03	61.4	0.09	9.89	1.17	1.94	56.5	0.26	40.3	4.31	217	44.8	0.15
from porphyry	WL-Y-11(17)-SP.96	0.15	5.21	0.02	61.5	0.10	19.8	1.24	0.42	60.8	0.21	41.3	4.83	213	13.6	b.d.l.
from porphyry	WL-Y-11(17)-SP.97	0.15	5.26	0.03	61.5	0.10	19.9	1.43	0.54	69.6	0.20	42.7	4.48	215	53.0	b.d.l.
from porphyry	WL-Y-11(17)-SP.98	0.16	5.35	0.03	61.4	0.09	19.6	1.41	1.45	43.1	0.26	39.4	5.19	220	43.8	0.11
from porphyry	WL-Y-11(17)-SP.99	0.15	5.33	0.03	61.4	0.10	9.99	1.20	0.98	97.6	b.d.l.	42.3	4.17	223	31.4	0.73
from porphyry	WL-Y-11(17)-SP.100	0.15	5.22	0.03	61.5	0.10	9.42	1.11	1.19	98.1	b.d.l.	41.3	3.46	227	32.0	0.08
from porphyry	WL-Y-11(17)-SP.101	0.15	5.19	0.03	61.5	0.10	8.26	1.11	0.30	94.6	0.28	41.8	4.49	214	81.3	b.d.l.
from porphyry	WL-Y-11(17)-SP.102	0.15	5.08	0.03	61.7	0.10	17.0	1.26	b.d.l.	92.9	b.d.l.	42.1	4.81	212	4.38	0.73
from porphyry	WL-Y-11(17)-SP.103	0.15	5.23	0.02	61.5	0.10	9.92	1.22	b.d.l.	98.6	0.10	41.7	3.30	221	11.9	b.d.l.
from porphyry	WL-Y-11(17)-SP.104	0.15	5.26	0.03	61.5	0.10	7.70	1.37	0.44	72.3	0.12	40.6	3.50	226	35.3	b.d.l.
from porphyry	WL-Y-11(17)-SP.105	0.14	5.22	0.03	61.5	0.10	5.92	1.40	0.30	69.8	0.24	42.1	3.99	229	56.4	b.d.l.
from porphyry	WL-Y-4-SP.106	0.16	5.25	0.06	61.5	0.10	20.6	1.33	15.1	24.4	0.45	38.8	4.40	92.3	18.9	0.60
from porphyry	WL-Y-4-SP.107	0.15	4.71	0.15	61.9	0.10	18.8	1.07	2.98	30.4	b.d.l.	38.9	7.93	84.7	19.2	0.31
from porphyry	WL-Y-4-SP.108	0.15	5.21	0.09	61.5	0.11	19.9	1.11	0.31	51.2	b.d.l.	41.6	28.6	95.5	39.6	0.09
from porphyry	WL-Y-4-SP.109	0.16	5.33	0.01	61.4	0.10	19.1	1.20	0.71	54.9	b.d.l.	41.7	16.2	95.5	10.5	0.23
from porphyry	WL-Y-4-SP.110	0.15	5.37	0.15	61.2	0.10	20.0	0.99	2.35	63.0	b.d.l.	42.1	14.1	104	39.8	0.15
from porphyry	WL-Y-4-SP.111	0.13	4.77	0.45	61.6	0.10	17.9	1.48	4.53	61.8	0.19	38.5	66.2	91.3	86.2	135
from porphyry	WL-Y-4-SP.112	0.15	5.08	0.12	61.6	0.10	18.2	1.25	10.5	9.48	0.34	41.7	218	86.2	32.4	312
from porphyry	WL-Y-4-SP.113	0.16	5.33	0.01	61.4	0.11	20.5	1.08	0.84	51.1	b.d.l.	42.2	2.46	83.4	29.0	0.15
from porphyry	WL-Y-4-SP.114	0.15	5.41	0.01	61.3	0.10	20.5	1.11	0.97	43.0	b.d.l.	40.4	2.92	97.6	12.1	0.16
from porphyry	WL-Y-4-SP.115	0.14	5.05	0.03	61.7	0.10	18.5	1.01	2.59	31.9	0.96	40.7	17.5	91.9	70.8	3.72
from porphyry	WL-Y-4-SP.116	0.15	5.48	0.01	61.3	0.10	20.5	1.29	0.95	60.0	b.d.l.	41.4	3.68	93.2	14.2	0.09
from porphyry	WL-Y-4-SP.117	0.15	5.19	0.01	61.6	0.10	20.5	1.00	1.30	49.1	b.d.l.	40.3	1.99	95.4	18.9	b.d.l.
from porphyry	WL-Y-4-SP.118	0.15	5.27	0.04	61.4	0.10	19.7	0.93	1.63	38.3	b.d.l.	39.4	37.3	95	22.5	10.6
from porphyry	WL-Y-4-SP.119	0.15	5.31	0.08	61.4	0.10	19.6	1.11	1.99	47.0	0.15	40.1	13.2	90.9	7.57	2.57
from porphyry	WL-Y-4-SP.120	0.15	5.38	0.01	61.4	0.10	19.3	1.10	2.51	60.5	b.d.l.	40.8	13.0	94.0	5.05	2.25
from porphyry	WL-Y-4-SP.121	0.14	5.37	0.02	61.3	0.10	10.2	1.09	1.44	98.1	b.d.l.	41.4	8.77	109	42.6	0.57

from porphyry	WL-Y-4-SP.122	0.13	5.20	0.26	61.3	0.10	17.3	1.12	1.76	44.1	b.d.l.	39.0	38.1	88.2	11.4	6.78
from porphyry	WL-Y-4-SP.123	0.15	5.31	0.12	61.3	0.10	17.4	1.23	0.85	25.7	b.d.l.	40.6	16.5	87.9	13.4	3.72
from porphyry	WL-Y-4-SP.124	0.15	5.40	0.04	61.4	0.10	20.5	0.94	0.70	27.1	0.13	41.4	6.12	90.4	27.7	b.d.l.
from porphyry	WL-Y-4-SP.125	0.14	5.26	0.07	61.8	0.10	20.6	1.14	0.31	33.4	b.d.l.	41.2	36.9	91.9	189	1.59
from porphyry	WL-Y-4-SP.126	0.15	4.92	0.04	55.2	0.10	19.2	1.04	b.d.l.	25.1	b.d.l.	39.8	5.60	71.0	21.9	0.06
from crystal	WQ-6(6)-SP.1	0.02	0.97	0.32	65.7	0.15	26.0	1.16	b.d.l.	350	b.d.l.	55.3	3.56	196	2280	0.30
from crystal	WQ-6(6)-SP.2	0.02	0.89	0.07	66.0	0.14	17.8	1.09	b.d.l.	314	b.d.l.	55.0	2.01	185	309	0.39
from crystal	WQ-6(6)-SP.3	0.03	1.07	0.33	65.6	0.14	34.1	1.50	b.d.l.	330	b.d.l.	53.6	2.64	199	1520	0.18
from crystal	WQ-6(6)-SP.4	0.02	0.92	0.04	66.0	0.14	17.5	0.96	b.d.l.	297	0.31	54.9	1.55	183	85.1	0.25
from crystal	WQ-6(6)-SP.5	0.03	1.03	0.18	65.7	0.14	39.8	1.17	b.d.l.	323	b.d.l.	52.0	1.81	200	773	0.14
from crystal	WQ-6(6)-SP.6	0.04	1.05	0.09	65.8	0.13	25.3	0.58	b.d.l.	66.2	b.d.l.	51.1	0.94	233	368	b.d.l.
from crystal	WQ-6(6)-SP.7	0.04	1.10	0.17	65.7	0.13	20.0	0.57	b.d.l.	71.7	b.d.l.	52.3	1.70	257	599	b.d.l.
from crystal	WQ-6(6)-SP.8	0.04	1.13	0.29	65.5	0.13	22.5	0.72	b.d.l.	70.1	b.d.l.	52.8	3.17	268	1180	0.09
from crystal	WQ-6(6)-SP.9	0.04	1.14	0.03	65.8	0.13	21.3	0.51	b.d.l.	53.5	b.d.l.	51.2	0.51	263	10.2	b.d.l.
from crystal	WQ-6(6)-SP.10	0.04	1.05	0.08	65.8	0.13	29.2	1.44	b.d.l.	420	b.d.l.	52.0	0.97	260	188	b.d.l.
from crystal	WQ-6(6)-SP.11	0.04	1.05	0.08	65.8	0.13	21.2	0.57	b.d.l.	68.0	b.d.l.	51.7	1.00	255	299	b.d.l.
from crystal	WQ-6(6)-SP.12	0.02	0.93	0.13	65.9	0.14	19.7	0.75	b.d.l.	76.5	b.d.l.	55.2	9.17	206	598	1.75
from crystal	WQ-6(6)-SP.13	0.04	1.05	0.03	65.9	0.13	24.6	0.77	b.d.l.	154	b.d.l.	52.6	0.51	231	21.7	b.d.l.
from crystal	WQ-6(6)-SP.13	0.03	0.92	0.91	65.1	0.14	31.2	2.62	b.d.l.	444	b.d.l.	54.1	8.48	263	8830	0.52
from crystal	WQ-6(6)-SP.14	0.03	1.04	0.48	65.4	0.14	44.2	1.59	b.d.l.	337	b.d.l.	53.1	5.17	217	3550	0.37
from crystal	WQ-6(6)-SP.15	0.03	1.03	0.06	65.9	0.13	22.9	0.44	b.d.l.	66.8	b.d.l.	51.3	1.87	217	207	1.26
from crystal	WQ-6(6)-SP.16	0.02	1.03	0.07	65.9	0.14	19.2	0.67	0.37	18.0	5.51	54.9	6.15	208	457	6.79
from crystal	WQ-6(6)-SP.17	0.02	0.91	0.17	65.9	0.14	18.6	0.51	b.d.l.	47.3	b.d.l.	54.1	5.75	198	1060	1.28
from crystal	WQ-6(6)-SP.18	0.04	1.10	0.04	65.8	0.13	20.8	0.63	b.d.l.	106	b.d.l.	52.2	0.60	216	122	b.d.l.
from crystal	WQ-6(6)-SP.19	0.03	1.02	0.25	65.7	0.13	46.1	0.94	b.d.l.	233	b.d.l.	50.7	1.99	228	1250	0.11
from crystal	WQ-6(6)-SP.20	0.02	0.99	0.60	65.4	0.15	17.8	0.79	b.d.l.	82.4	b.d.l.	55.5	8.31	223	7640	0.98
from crystal	WQ-6(6)-SP.21	0.03	1.11	0.79	65.0	0.14	35.8	1.55	b.d.l.	343	b.d.l.	54.1	8.91	217	5530	0.69
from crystal	WQ-6(6)-SP.22	0.04	1.09	0.08	65.8	0.13	22.0	0.71	b.d.l.	68.2	b.d.l.	51.6	0.99	264	230	b.d.l.

Detection limits (ppm)

	Sample	Mn	Fe	Cu	Zn	Cd	Ga	Ge	As	Se	Mo	Pd	Ag	In	Sn	Sb
Quartz vein;Cu-Zn deposit	NW-19-SP.44	0.33	15.0	0.49	2.55	0.45	0.03	0.42	0.14	0.73	0.06	0.09	0.09	0.01	0.06	0.07
Quartz vein;Cu-Zn deposit	NW-19-SP.45	0.30	14.1	0.50	2.75	0.35	0.03	0.39	0.13	0.65	0.05	0.09	0.09	0.01	0.05	0.06
Quartz vein;Cu-Zn deposit	NW-19-SP.46	0.30	13.2	0.50	3.03	0.35	0.03	0.41	0.15	0.66	0.05	0.09	0.08	0.01	0.05	0.06
Quartz vein;Cu-Zn deposit	NW-19-SP.47	0.29	14.0	0.47	3.02	0.37	0.03	0.42	0.17	0.80	0.05	0.10	0.08	0.01	0.05	0.06
Quartz vein;Cu-Zn deposit	NW-19-SP.48	0.37	15.9	0.59	3.88	0.40	0.03	0.43	0.19	0.89	0.06	0.12	0.09	0.01	0.06	0.06
Quartz vein;Cu-Zn deposit	NW-19-SP.49	0.44	18.7	0.71	2.51	0.39	0.05	0.61	0.23	0.96	0.08	0.14	0.10	0.01	0.07	0.07
Quartz vein;Cu-Zn deposit	NW-4-7-SP.37	0.56	22.9	0.90	5.67	0.68	0.07	0.64	0.35	6.95	0.16	0.19	0.19	0.02	0.08	0.08
Quartz vein;Cu-Zn deposit	NW-4-7-SP.38	0.50	20.2	0.82	4.78	0.56	0.06	0.59	0.34	5.22	0.13	0.15	0.19	0.02	0.08	0.07
Quartz vein;Cu-Zn deposit	NW-4-7-SP.39	0.51	20.2	0.76	4.51	0.50	0.06	0.55	0.39	6.90	0.14	0.16	0.16	0.02	0.09	0.09
Quartz vein;Cu-Zn deposit	NW-4-7-SP.40	0.49	20.9	0.87	4.53	0.55	0.07	0.60	0.39	7.35	0.15	0.16	0.19	0.02	0.09	0.09
Quartz vein;Cu-Zn deposit	NW-4-7-SP.41	0.54	23.2	0.99	4.39	0.57	0.07	0.65	0.39	6.54	0.15	0.17	0.20	0.02	0.09	0.08
Quartz vein;Cu-Zn deposit	NW-4-7-SP.42	0.78	26.7	1.11	4.36	0.53	0.08	0.68	0.37	6.44	0.17	0.18	0.22	0.02	0.09	0.09
Quartz vein;Sn deposit	NWN-3-SP.58	0.27	10.3	0.43	2.13	0.23	0.02	0.35	0.12	0.61	0.04	0.08	0.06	0.01	0.04	0.04
Quartz vein;Sn deposit	NWN-3-SP.59	0.27	9.30	0.45	2.50	0.25	0.03	0.34	0.13	0.59	0.06	0.07	0.07	0.01	0.05	0.04
Quartz vein;Sn deposit	NWN-3-SP.60	0.28	9.20	0.43	2.43	0.27	0.03	0.34	0.13	0.63	0.05	0.07	0.07	0.01	0.04	0.04
Quartz vein;Sn deposit	NWN-3-SP.61	0.27	10.5	0.43	2.74	0.28	0.03	0.32	0.14	0.65	0.05	0.08	0.07	0.01	0.05	0.04
Quartz vein;Sn deposit	NWN-3-SP.62	0.28	11.8	0.40	2.89	0.29	0.03	0.36	0.13	0.64	0.05	0.08	0.07	0.01	0.05	0.05
Quartz vein;Sn deposit	NWN-3-SP.63	0.31	12.0	0.38	2.84	0.34	0.02	0.36	0.11	0.63	0.04	0.09	0.06	0.01	0.05	0.04
Quartz vein;Sn deposit	NWN-3-SP.64	0.30	11.4	0.40	3.26	0.33	0.02	0.35	0.12	0.59	0.04	0.09	0.07	0.01	0.04	0.04
Quartz vein;Sn deposit	NWN-3-SP.65	0.26	11.0	0.41	3.75	0.31	0.03	0.36	0.12	0.65	0.04	0.08	0.07	0.01	0.04	0.05
Quartz vein;Sn deposit	NWN-29-SP.50	0.39	22.9	0.83	2.23	0.29	0.04	0.50	0.26	5.38	0.12	0.12	0.13	0.02	0.06	0.07
Quartz vein;Sn deposit	NWN-29-SP.51	0.36	23.0	0.76	4.02	0.34	0.05	0.48	0.27	5.54	0.11	0.12	0.13	0.02	0.07	0.08
Quartz vein;Sn deposit	NWN-29-SP.52	0.37	25.1	0.80	5.08	0.36	0.06	0.52	0.31	5.91	0.12	0.11	0.12	0.01	0.07	0.09
Quartz vein;Sn deposit	NWN-29-SP.53	0.36	26.1	0.83	5.18	0.32	0.05	0.49	0.30	5.50	0.11	0.10	0.12	0.01	0.07	0.08
Quartz vein;Sn deposit	NWN-29-SP.54	0.38	27.0	0.93	6.41	0.35	0.05	0.51	0.27	5.72	0.11	0.11	0.12	0.01	0.07	0.07
Quartz vein;Sn deposit	NWN-29-SP.55	0.40	24.9	0.88	6.88	0.36	0.06	0.55	0.24	5.91	0.10	0.12	0.13	0.01	0.07	0.06

Quartz vein;Sn deposit	NWN-29-SP.56	0.37	23.8	0.79	7.10	0.33	0.06	0.54	0.25	6.30	0.09	0.10	0.13	0.01	0.07	0.07
Quartz vein;Sn deposit	NWN-29-SP.57	0.39	24.7	0.85	7.52	0.38	0.07	0.53	0.31	6.28	0.12	0.11	0.13	0.01	0.07	0.08
Quartz vein;Sn deposit	WL-6-13-SP.66	0.41	22.0	0.83	3.63	0.34	0.06	0.60	0.30	6.45	0.11	0.11	0.13	0.01	0.07	0.08
Quartz vein;Sn deposit	WL-6-13-SP.67	0.33	17.6	0.61	3.78	0.33	0.04	0.45	0.22	4.62	0.09	0.09	0.11	0.01	0.05	0.06
Quartz vein;Sn deposit	WL-6-13-SP.68	0.30	16.2	0.59	4.91	0.34	0.04	0.42	0.19	4.17	0.08	0.08	0.10	0.01	0.05	0.06
Quartz vein;Sn deposit	WL-6-13-SP.69	0.32	15.7	0.61	5.37	0.28	0.04	0.44	0.22	4.23	0.08	0.08	0.10	0.01	0.04	0.06
Quartz vein;Sn deposit	WL-6-13-SP.70	0.34	19.1	0.60	6.08	0.30	0.04	0.45	0.24	4.59	0.08	0.09	0.10	0.01	0.04	0.07
Quartz vein;Sn deposit	WL-6-13-SP.71	0.33	20.2	0.66	6.53	0.31	0.04	0.42	0.24	4.83	0.08	0.11	0.10	0.01	0.05	0.07
Quartz vein;Sn deposit	WL-6-13-SP.72	0.31	18.1	0.66	6.46	0.32	0.04	0.41	0.21	4.98	0.07	0.10	0.10	0.01	0.05	0.06
Quartz vein;Sn deposit	WL-6-3-SP.78	0.34	18.1	0.66	3.27	0.35	0.04	0.45	0.21	4.38	0.09	0.09	0.11	0.01	0.05	0.07
Quartz vein;Sn deposit	WL-6-3-SP.79	0.36	16.3	0.71	4.82	0.37	0.04	0.46	0.21	4.55	0.08	0.09	0.11	0.01	0.05	0.06
Quartz vein;Sn deposit	WL-6-3-SP.80	0.38	15.0	0.75	5.91	0.35	0.04	0.44	0.22	4.52	0.08	0.08	0.11	0.01	0.05	0.06
Quartz vein;Sn deposit	WL-6-3-SP.81	0.32	15.2	0.76	5.49	0.37	0.04	0.43	0.22	4.54	0.07	0.08	0.12	0.01	0.06	0.06
Quartz vein;Sn deposit	WL-6-3-SP.82	0.29	17.5	0.62	3.13	0.30	0.04	0.47	0.22	4.39	0.08	0.09	0.11	0.01	0.06	0.06
Quartz vein;Sn deposit	WL-6-3-SP.83	0.33	17.6	0.62	4.46	0.33	0.05	0.47	0.21	4.34	0.09	0.09	0.12	0.01	0.05	0.06
Quartz vein;Sn deposit	WL-6(2)-SP.84	0.34	14.8	0.65	5.01	0.31	0.04	0.44	0.23	4.66	0.10	0.08	0.11	0.01	0.04	0.06
Quartz vein;Sn deposit	WL-6(2)-SP.85	0.37	16.4	0.62	5.85	0.28	0.04	0.48	0.23	5.05	0.09	0.08	0.10	0.01	0.05	0.06
Quartz vein;Sn deposit	WL-6(2)-SP.86	0.33	16.3	0.64	6.03	0.30	0.04	0.44	0.23	4.60	0.08	0.08	0.10	0.01	0.05	0.06
Quartz vein;Sn deposit	WL-6(2)-SP.87	0.32	15.1	0.67	6.09	0.32	0.04	0.41	0.24	3.84	0.08	0.08	0.10	0.01	0.05	0.06
Quartz vein;Sn deposit	WL-6(2)-SP.88	0.32	16.9	0.67	6.37	0.31	0.04	0.46	0.23	3.96	0.08	0.09	0.11	0.01	0.05	0.06
from porphyry	WL-Y-11(17)-SP.89	0.38	18.0	0.80	6.77	0.38	0.05	0.53	0.29	5.59	0.10	0.13	0.12	0.01	0.06	0.07
from porphyry	WL-Y-11(17)-SP.90	0.37	18.7	0.82	3.59	0.39	0.06	0.49	0.31	5.43	0.08	0.09	0.13	0.01	0.05	0.07
from porphyry	WL-Y-11(17)-SP.91	0.43	17.7	0.85	3.98	0.39	0.05	0.49	0.32	5.20	0.08	0.12	0.13	0.01	0.06	0.07
from porphyry	WL-Y-11(17)-SP.92	0.58	21.3	0.97	5.64	0.50	0.05	0.66	0.37	6.47	0.11	0.14	0.15	0.02	0.08	0.09
from porphyry	WL-Y-11(17)-SP.93	0.45	21.7	0.82	5.53	0.44	0.05	0.60	0.32	5.74	0.10	0.12	0.15	0.01	0.08	0.08
from porphyry	WL-Y-11(17)-SP.94	0.39	19.7	0.76	5.03	0.35	0.05	0.53	0.30	5.07	0.10	0.11	0.13	0.01	0.08	0.07
from porphyry	WL-Y-11(17)-SP.95	0.42	18.6	0.73	4.94	0.30	0.05	0.50	0.26	4.96	0.10	0.11	0.13	0.01	0.07	0.08
from porphyry	WL-Y-11(17)-SP.96	0.38	18.1	0.72	5.19	0.36	0.04	0.48	0.23	4.55	0.09	0.09	0.12	0.01	0.05	0.07
from porphyry	WL-Y-11(17)-SP.97	0.37	18.1	0.78	5.50	0.42	0.04	0.55	0.22	5.03	0.10	0.09	0.13	0.01	0.06	0.07

from porphyry	WL-Y-11(17)-SP.98	0.41	17.6	0.80	3.29	0.33	0.06	0.50	0.27	5.19	0.12	0.11	0.14	0.02	0.08	0.08
from porphyry	WL-Y-11(17)-SP.99	0.48	18.4	0.82	4.35	0.34	0.05	0.55	0.28	5.95	0.12	0.11	0.13	0.01	0.07	0.09
from porphyry	WL-Y-11(17)-SP.100	0.41	16.7	0.77	4.96	0.34	0.06	0.50	0.30	5.42	0.11	0.11	0.12	0.02	0.06	0.07
from porphyry	WL-Y-11(17)-SP.101	0.39	16.0	0.71	4.51	0.34	0.06	0.45	0.26	4.88	0.10	0.12	0.12	0.02	0.07	0.07
from porphyry	WL-Y-11(17)-SP.102	0.41	16.6	0.68	4.78	0.35	0.05	0.46	0.24	4.85	0.09	0.12	0.12	0.01	0.06	0.08
from porphyry	WL-Y-11(17)-SP.103	0.35	18.0	0.69	5.32	0.35	0.05	0.48	0.27	4.66	0.08	0.12	0.13	0.01	0.06	0.08
from porphyry	WL-Y-11(17)-SP.104	0.38	18.3	0.65	5.26	0.38	0.05	0.49	0.26	4.76	0.08	0.13	0.12	0.01	0.06	0.08
from porphyry	WL-Y-11(17)-SP.105	0.41	17.9	0.65	5.14	0.38	0.05	0.51	0.23	5.14	0.09	0.12	0.12	0.01	0.06	0.08
from porphyry	WL-Y-4-SP.106	0.49	18.9	0.92	2.51	0.40	0.06	0.65	0.31	6.53	0.16	0.14	0.16	0.01	0.09	0.09
from porphyry	WL-Y-4-SP.107	0.37	15.0	0.68	2.95	0.36	0.05	0.47	0.25	5.03	0.12	0.11	0.12	0.01	0.06	0.07
from porphyry	WL-Y-4-SP.108	0.36	15.4	0.70	3.44	0.40	0.05	0.46	0.22	4.63	0.11	0.12	0.13	0.01	0.05	0.06
from porphyry	WL-Y-4-SP.109	0.38	16.6	0.73	3.58	0.38	0.05	0.48	0.22	4.38	0.10	0.13	0.13	0.01	0.05	0.06
from porphyry	WL-Y-4-SP.110	0.36	16.6	0.75	3.77	0.34	0.05	0.44	0.23	4.15	0.09	0.13	0.13	0.02	0.05	0.07
from porphyry	WL-Y-4-SP.111	0.39	16.1	0.79	4.57	0.37	0.06	0.47	0.24	4.69	0.07	0.15	0.13	0.02	0.06	0.08
from porphyry	WL-Y-4-SP.112	0.40	16.1	0.74	5.36	0.43	0.05	0.49	0.24	4.49	0.08	0.14	0.13	0.01	0.07	0.07
from porphyry	WL-Y-4-SP.113	0.35	15.7	0.77	5.28	0.49	0.05	0.45	0.28	3.93	0.08	0.13	0.13	0.01	0.06	0.07
from porphyry	WL-Y-4-SP.114	0.36	15.2	0.76	5.11	0.47	0.04	0.47	0.26	4.31	0.07	0.13	0.13	0.01	0.06	0.07
from porphyry	WL-Y-4-SP.115	0.40	15.2	0.73	5.66	0.38	0.04	0.48	0.22	4.80	0.08	0.12	0.12	0.01	0.05	0.07
from porphyry	WL-Y-4-SP.116	0.37	16.0	0.69	6.42	0.40	0.05	0.45	0.24	4.44	0.09	0.12	0.13	0.01	0.06	0.07
from porphyry	WL-Y-4-SP.117	0.33	15.6	0.61	6.43	0.49	0.05	0.44	0.28	3.91	0.11	0.10	0.13	0.01	0.06	0.07
from porphyry	WL-Y-4-SP.118	0.36	18.4	0.73	3.15	0.42	0.05	0.42	0.25	5.15	0.08	0.10	0.13	0.01	0.05	0.07
from porphyry	WL-Y-4-SP.119	0.39	17.1	0.72	4.23	0.45	0.05	0.43	0.23	4.50	0.08	0.11	0.14	0.01	0.05	0.06
from porphyry	WL-Y-4-SP.120	0.38	15.4	0.80	4.67	0.41	0.05	0.47	0.25	4.32	0.09	0.10	0.12	0.01	0.06	0.07
from porphyry	WL-Y-4-SP.121	0.37	15.2	0.75	5.01	0.40	0.06	0.47	0.25	4.93	0.09	0.11	0.12	0.01	0.06	0.08
from porphyry	WL-Y-4-SP.122	0.32	15.2	0.63	4.70	0.46	0.06	0.45	0.27	4.64	0.10	0.12	0.11	0.01	0.06	0.07
from porphyry	WL-Y-4-SP.123	0.28	14.5	0.58	4.60	0.45	0.05	0.45	0.27	3.92	0.11	0.12	0.11	0.01	0.05	0.08
from porphyry	WL-Y-4-SP.124	0.33	16.2	0.62	5.98	0.38	0.05	0.47	0.25	4.46	0.09	0.13	0.13	0.01	0.06	0.07
from porphyry	WL-Y-4-SP.125	0.36	16.3	0.67	5.63	0.41	0.05	0.44	0.22	4.18	0.08	0.11	0.12	0.01	0.06	0.05
from porphyry	WL-Y-4-SP.126	0.36	16.4	0.75	5.44	0.41	0.05	0.46	0.23	4.13	0.08	0.10	0.12	0.01	0.06	0.05

from crystal	WQ-6(6)-SP.1	0.34	15.0	0.69	4.31	0.55	0.05	0.41	0.23	3.45	0.06	0.13	0.12	0.01	0.05	0.06
from crystal	WQ-6(6)-SP.2	0.38	15.3	0.67	2.72	0.43	0.05	0.44	0.26	4.48	0.10	0.11	0.11	0.01	0.06	0.06
from crystal	WQ-6(6)-SP.3	0.30	13.1	0.59	3.69	0.42	0.04	0.45	0.26	4.26	0.09	0.11	0.10	0.01	0.05	0.06
from crystal	WQ-6(6)-SP.4	0.29	13.7	0.67	4.77	0.49	0.04	0.43	0.26	4.44	0.08	0.10	0.11	0.01	0.05	0.06
from crystal	WQ-6(6)-SP.5	0.37	14.9	0.77	5.51	0.54	0.04	0.44	0.24	4.18	0.08	0.11	0.13	0.01	0.06	0.06
from crystal	WQ-6(6)-SP.6	0.39	16.2	0.78	5.84	0.56	0.05	0.48	0.23	4.53	0.09	0.12	0.13	0.01	0.06	0.06
from crystal	WQ-6(6)-SP.7	0.38	17.9	0.78	5.65	0.59	0.06	0.48	0.25	4.62	0.09	0.12	0.13	0.01	0.06	0.07
from crystal	WQ-6(6)-SP.8	0.38	17.9	0.72	5.96	0.57	0.05	0.51	0.30	4.43	0.08	0.13	0.14	0.01	0.06	0.07
from crystal	WQ-6(6)-SP.9	0.30	14.3	0.71	5.28	0.48	0.05	0.45	0.27	3.96	0.07	0.11	0.12	0.01	0.06	0.06
from crystal	WQ-6(6)-SP.10	0.36	14.7	0.77	5.69	0.55	0.05	0.46	0.30	4.19	0.09	0.12	0.14	0.01	0.07	0.06
from crystal	WQ-6(6)-SP.11	0.39	17.1	0.75	6.21	0.57	0.06	0.48	0.28	4.33	0.10	0.12	0.14	0.01	0.06	0.06
from crystal	WQ-6(6)-SP.12	0.41	19.0	0.82	5.86	0.56	0.06	0.45	0.26	4.94	0.11	0.13	0.15	0.01	0.07	0.06
from crystal	WQ-6(6)-SP.13	0.41	16.1	0.72	5.42	0.54	0.04	0.42	0.24	5.04	0.12	0.13	0.13	0.01	0.05	0.06
from crystal	WQ-6(6)-SP.13	1.08	42.9	1.93	14.50	1.47	0.12	1.13	0.65	13.49	0.32	0.34	0.37	0.03	0.14	0.17
from crystal	WQ-6(6)-SP.14	0.45	16.6	0.78	6.23	0.60	0.05	0.48	0.26	5.51	0.12	0.14	0.14	0.01	0.06	0.07
from crystal	WQ-6(6)-SP.15	0.38	15.3	0.70	3.43	0.48	0.05	0.42	0.21	3.66	0.11	0.10	0.13	0.01	0.06	0.06
from crystal	WQ-6(6)-SP.16	0.48	21.6	0.90	7.07	0.69	0.07	0.58	0.33	5.51	0.14	0.15	0.18	0.02	0.08	0.09
from crystal	WQ-6(6)-SP.17	0.36	15.6	0.71	5.75	0.56	0.05	0.46	0.29	4.37	0.10	0.12	0.14	0.01	0.06	0.07
from crystal	WQ-6(6)-SP.18	0.31	13.7	0.66	4.33	0.52	0.04	0.39	0.24	3.56	0.09	0.11	0.13	0.01	0.07	0.05
from crystal	WQ-6(6)-SP.19	0.40	16.6	0.72	5.31	0.52	0.05	0.46	0.26	4.20	0.10	0.13	0.14	0.01	0.08	0.06
from crystal	WQ-6(6)-SP.20	0.43	17.8	0.73	6.00	0.47	0.05	0.51	0.26	4.87	0.09	0.14	0.15	0.01	0.07	0.07
from crystal	WQ-6(6)-SP.21	0.40	19.4	0.73	5.35	0.51	0.05	0.49	0.26	5.22	0.11	0.15	0.15	0.02	0.06	0.07
from crystal	WQ-6(6)-SP.22	0.38	17.9	0.65	4.73	0.51	0.05	0.48	0.27	5.11	0.12	0.14	0.13	0.02	0.05	0.06

2. Bairendaba deposit

Measured concentrations

Sample	S	Zn	Mn	Fe	Cd	Cu	V	Cr	Co	Ga	Ge	As	Se	Ag	In	Sn	Sb
	wt.%	wt.%	wt.%	wt.%	wt.%	wt.%	ppm	ppm	ppm	ppm	ppm	ppm	ppm	ppm	ppm	ppm	ppm
vein type;WB-97 - 1	33.3	58.4	0.07	7.98	0.29	0.01	0.05	b.d.l.	0.28	0.93	1.34	b.d.l.	1.82	3.79	182	3.08	b.d.l.
vein type;WB-97 - 2	33.3	58.3	0.07	8.09	0.29	0.01	0.07	b.d.l.	0.23	1.07	1.57	b.d.l.	1.69	3.71	191	2.93	0.17
vein type;WB-97 - 3	33.3	58.2	0.07	8.09	0.30	0.02	0.09	b.d.l.	0.25	1.50	1.31	b.d.l.	2.78	4.26	200	2.25	b.d.l.
vein type;WB-97 - 4	33.3	58.2	0.07	8.14	0.31	0.02	0.07	b.d.l.	0.27	1.73	1.30	b.d.l.	2.62	4.06	209	2.22	b.d.l.
vein type;WB-97 - 5	33.3	58.1	0.07	8.24	0.31	0.02	0.07	0.68	0.24	1.77	1.37	b.d.l.	3.03	5.37	214	2.71	b.d.l.
vein type;WB-97 - 6	33.3	58.2	0.07	8.15	0.31	0.02	0.09	b.d.l.	0.24	1.98	1.73	b.d.l.	2.86	5.12	213	6.35	b.d.l.
vein type;WB-97 - 7	33.2	58.8	0.07	7.57	0.32	0.02	0.07	b.d.l.	0.19	1.27	1.33	b.d.l.	4.52	5.20	210	2.45	b.d.l.
vein type;WB-97 - 8	33.3	58.3	0.08	8.03	0.32	0.02	0.05	0.51	0.16	1.50	1.26	b.d.l.	3.13	6.79	207	2.36	b.d.l.
vein type;WB-97 - 9	33.2	58.9	0.08	7.45	0.31	0.02	0.06	b.d.l.	0.12	1.67	1.40	b.d.l.	1.92	6.46	219	2.42	b.d.l.
vein type;WB-97 - 10	33.3	58.5	0.08	7.80	0.31	0.02	0.07	b.d.l.	0.14	1.83	1.52	b.d.l.	1.88	5.43	210	3.28	b.d.l.
vein type;WB-97 - 11	33.3	58.0	0.08	8.31	0.28	0.02	0.04	b.d.l.	0.19	1.16	1.44	b.d.l.	2.85	5.94	230	3.20	b.d.l.
vein type;WB-97 - 12	33.3	57.9	0.08	8.42	0.28	0.02	0.06	b.d.l.	0.14	1.21	1.42	b.d.l.	2.84	6.44	230	2.99	b.d.l.
vein type;WB-97 - 13	33.3	57.9	0.08	8.44	0.29	0.02	0.03	b.d.l.	0.15	1.25	1.52	b.d.l.	2.65	7.41	231	3.41	b.d.l.
vein type;WB-97 - 14	33.3	58.0	0.08	8.35	0.29	0.02	0.03	b.d.l.	0.15	1.38	1.62	b.d.l.	3.35	6.74	227	3.20	b.d.l.
vein type;WB-97 - 15	33.3	58.0	0.09	8.33	0.29	0.02	0.03	b.d.l.	0.13	1.75	b.d.l.	b.d.l.	2.12	6.54	230	3.71	b.d.l.
vein type;WB-97 - 16	33.3	58.1	0.09	8.17	0.28	0.02	0.03	b.d.l.	0.13	2.60	1.50	b.d.l.	1.75	7.72	230	2.52	b.d.l.
vein type;WB-97 - 17	33.3	58.4	0.09	7.96	0.28	0.02	0.06	b.d.l.	0.07	2.13	b.d.l.	b.d.l.	2.59	7.08	230	2.76	b.d.l.
vein type;WB-97 - 18	33.3	58.6	0.09	7.75	0.29	0.02	0.03	b.d.l.	0.08	1.51	1.49	b.d.l.	1.41	5.85	225	4.25	b.d.l.
vein type;WB-97 - 19	33.2	59.0	0.08	7.35	0.29	0.02	0.04	b.d.l.	0.06	2.39	1.20	b.d.l.	1.36	7.19	224	2.93	b.d.l.
vein type;WB-97 - 20	33.3	58.6	0.09	7.76	0.28	0.02	0.04	b.d.l.	0.07	1.37	1.34	b.d.l.	1.10	6.95	229	2.81	b.d.l.
vein type;WB-92 - 1	33.2	59.4	0.07	7.01	0.27	0.02	0.06	b.d.l.	0.04	1.85	1.32	b.d.l.	b.d.l.	5.73	243	1.56	b.d.l.
vein type;WB-92 - 2	33.2	59.6	0.07	6.82	0.27	0.02	0.04	b.d.l.	0.08	1.87	0.82	0.97	1.35	5.31	243	1.38	b.d.l.
vein type;WB-92 - 3	33.3	58.6	0.09	7.80	0.26	0.02	0.07	b.d.l.	0.04	1.33	b.d.l.	b.d.l.	0.90	3.14	239	2.83	b.d.l.

vein type;WB-92 - 4	33.3	58.1	0.09	8.24	0.28	0.02	0.03	b.d.l.	0.05	1.78	1.46	b.d.l.	1.37	4.08	234	3.91	b.d.l.
vein type;WB-92 - 5	33.3	58.0	0.09	8.32	0.28	0.02	b.d.l.	b.d.l.	0.03	3.07	1.30	b.d.l.	b.d.l.	3.93	247	3.12	b.d.l.
vein type;WB-92 - 6	33.3	57.9	0.10	8.41	0.28	0.02	b.d.l.	b.d.l.	0.06	2.35	1.70	b.d.l.	1.49	3.77	236	2.50	b.d.l.
vein type;WB-92 - 7	33.3	58.0	0.10	8.30	0.27	0.02	0.05	b.d.l.	0.04	3.12	1.35	0.70	1.95	3.61	234	2.31	b.d.l.
vein type;WB-92 - 8	33.3	58.1	0.10	8.19	0.27	0.02	0.05	b.d.l.	0.03	1.92	b.d.l.	b.d.l.	1.12	2.12	231	2.48	b.d.l.
vein type;WB-92 - 9	33.3	58.0	0.10	8.34	0.27	0.02	0.05	b.d.l.	0.03	2.28	1.52	b.d.l.	1.91	5.16	231	3.03	b.d.l.
vein type;WB-92 - 10	33.3	58.6	0.09	7.80	0.27	0.02	0.05	b.d.l.	0.03	1.93	1.19	b.d.l.	b.d.l.	3.10	241	0.99	b.d.l.
vein type;WB-91 - 6	33.3	57.8	0.10	8.54	0.29	0.02	0.03	b.d.l.	0.08	2.38	1.46	b.d.l.	2.31	4.63	227	2.27	b.d.l.
vein type;WB-91 - 7	33.3	57.7	0.09	8.62	0.29	0.02	0.03	b.d.l.	0.10	1.79	b.d.l.	b.d.l.	2.82	4.82	225	2.36	b.d.l.
vein type;WB-91 - 8	33.3	57.8	0.09	8.51	0.30	0.02	0.05	b.d.l.	0.08	2.58	1.35	b.d.l.	2.32	4.43	226	2.25	b.d.l.
vein type;WB-91 - 9	33.3	58.0	0.09	8.31	0.29	0.02	0.05	b.d.l.	0.09	2.58	1.35	b.d.l.	2.78	5.75	228	2.37	b.d.l.
vein type;WB-91 - 10	33.3	57.7	0.09	8.54	0.30	0.02	b.d.l.	b.d.l.	0.06	2.81	1.26	b.d.l.	3.72	4.97	227	2.40	b.d.l.

Detection limits (ppm)

Sample	Zn	Mn	Fe	Cd	Cu	V	Cr	Co	Ga	Ge	As	Se	Ag	In	Sn	Sb
vein type;WB-97 - 1	1.30	0.25	17.0	0.16	0.25	0.03	0.52	0.02	0.02	0.45	0.29	0.98	0.14	0.01	0.16	0.14
vein type;WB-97 - 2	1.32	0.25	17.2	0.16	0.25	0.03	0.51	0.02	0.03	0.46	0.29	0.94	0.14	0.01	0.16	0.15
vein type;WB-97 - 3	1.30	0.26	17.0	0.16	0.24	0.03	0.49	0.03	0.03	0.46	0.29	0.89	0.15	0.01	0.16	0.14
vein type;WB-97 - 4	1.28	0.26	16.8	0.16	0.23	0.03	0.49	0.03	0.03	0.46	0.28	0.86	0.15	0.01	0.16	0.14
vein type;WB-97 - 5	1.23	0.25	16.0	0.16	0.22	0.03	0.47	0.02	0.03	0.45	0.27	0.81	0.14	0.01	0.16	0.14
vein type;WB-97 - 6	1.22	0.25	15.7	0.15	0.22	0.03	0.48	0.02	0.03	0.45	0.26	0.81	0.14	0.01	0.16	0.14
vein type;WB-97 - 7	1.24	0.26	15.7	0.16	0.22	0.03	0.50	0.02	0.03	0.45	0.26	0.83	0.15	0.01	0.16	0.14
vein type;WB-97 - 8	1.14	0.24	14.1	0.14	0.21	0.03	0.47	0.02	0.02	0.42	0.24	0.78	0.13	0.01	0.14	0.13
vein type;WB-97 - 9	1.24	0.26	15.1	0.15	0.23	0.03	0.53	0.02	0.02	0.45	0.25	0.88	0.14	0.01	0.15	0.14
vein type;WB-97 - 10	1.26	0.26	15.0	0.15	0.24	0.03	0.56	0.02	0.02	0.45	0.25	0.93	0.14	0.01	0.15	0.14
vein type;WB-97 - 11	1.32	0.24	12.4	0.16	0.25	0.03	0.66	0.02	0.01	0.42	0.24	1.13	0.11	0.00	0.12	0.14
vein type;WB-97 - 12	1.48	0.26	13.6	0.18	0.27	0.03	0.74	0.02	0.02	0.47	0.26	1.26	0.12	0.00	0.13	0.15
vein type;WB-97 - 13	1.51	0.27	13.7	0.18	0.27	0.03	0.75	0.02	0.02	0.48	0.27	1.27	0.13	0.01	0.13	0.15

vein type;WB-97 - 14	1.49	0.26	13.3	0.18	0.27	0.03	0.74	0.02	0.02	0.47	0.26	1.24	0.12	0.01	0.13	0.15
vein type;WB-97 - 15	1.54	0.27	13.5	0.19	0.27	0.03	0.75	0.02	0.02	0.49	0.27	1.25	0.12	0.01	0.13	0.15
vein type;WB-97 - 16	1.38	0.24	12.0	0.17	0.24	0.03	0.67	0.02	0.01	0.44	0.24	1.09	0.11	0.01	0.12	0.14
vein type;WB-97 - 17	1.35	0.23	11.6	0.17	0.23	0.03	0.66	0.02	0.01	0.43	0.24	1.05	0.11	0.01	0.12	0.13
vein type;WB-97 - 18	1.55	0.27	13.3	0.20	0.26	0.03	0.74	0.02	0.02	0.49	0.28	1.16	0.13	0.01	0.13	0.15
vein type;WB-97 - 19	1.56	0.27	13.2	0.20	0.26	0.03	0.74	0.02	0.02	0.50	0.28	1.14	0.13	0.01	0.13	0.15
vein type;WB-97 - 20	1.49	0.26	12.6	0.19	0.25	0.03	0.70	0.02	0.02	0.48	0.27	1.06	0.12	0.01	0.13	0.15
vein type;WB-92 - 1	1.28	0.22	10.5	0.16	0.23	0.02	0.61	0.02	0.02	0.43	0.25	0.83	0.11	0.01	0.12	0.13
vein type;WB-92 - 2	1.59	0.28	13.0	0.20	0.29	0.03	0.76	0.03	0.03	0.53	0.31	1.05	0.14	0.01	0.15	0.17
vein type;WB-92 - 3	1.29	0.23	10.5	0.16	0.24	0.02	0.62	0.02	0.02	0.43	0.25	0.87	0.11	0.01	0.12	0.14
vein type;WB-92 - 4	1.26	0.22	10.1	0.15	0.24	0.02	0.61	0.02	0.02	0.42	0.25	0.87	0.11	0.01	0.12	0.13
vein type;WB-92 - 5	1.28	0.22	10.2	0.15	0.24	0.02	0.63	0.02	0.02	0.42	0.25	0.91	0.11	0.01	0.12	0.13
vein type;WB-92 - 6	1.34	0.23	10.6	0.15	0.26	0.03	0.67	0.02	0.02	0.44	0.27	0.99	0.11	0.01	0.13	0.14
vein type;WB-92 - 7	1.31	0.23	10.3	0.15	0.25	0.02	0.66	0.02	0.02	0.43	0.26	1.00	0.10	0.01	0.12	0.14
vein type;WB-92 - 8	1.32	0.23	10.2	0.15	0.25	0.03	0.67	0.02	0.02	0.42	0.27	1.03	0.10	0.01	0.12	0.14
vein type;WB-92 - 9	1.32	0.23	10.1	0.14	0.25	0.03	0.66	0.02	0.02	0.42	0.27	1.06	0.10	0.01	0.12	0.14
vein type;WB-92 - 10	1.34	0.23	10.3	0.15	0.25	0.03	0.68	0.02	0.02	0.43	0.27	1.11	0.10	0.01	0.12	0.14
vein type;WB-91 - 6	1.36	0.26	10.6	0.17	0.20	0.03	0.62	0.03	0.02	0.49	0.29	1.00	0.12	0.01	0.12	0.13
vein type;WB-91 - 7	1.41	0.27	11.1	0.18	0.22	0.03	0.64	0.03	0.02	0.51	0.30	1.00	0.13	0.01	0.13	0.14
vein type;WB-91 - 8	1.43	0.27	11.3	0.18	0.24	0.03	0.65	0.03	0.02	0.53	0.29	0.98	0.14	0.01	0.13	0.14
vein type;WB-91 - 9	1.38	0.27	11.0	0.17	0.25	0.03	0.64	0.03	0.03	0.52	0.28	0.93	0.14	0.01	0.13	0.14
vein type;WB-91 - 10	1.39	0.27	11.0	0.17	0.28	0.03	0.65	0.03	0.03	0.52	0.27	0.92	0.14	0.01	0.13	0.14

ESM Table 4. LA–ICP–MS analytical results for quartz from the Weilasituo deposit

Measured concentrations in quartz from different rock types

Sample	Lithology	Si wt.%	Li ppm	B ppm	Na ppm	Mg ppm	Al ppm	K ppm	Sc ppm	Ti ppm	Cu ppm	Zn ppm	Ga ppm	Ge ppm	Rb ppm	Sn ppm	Sb ppm	Cs ppm
WL-47(102).34	quartz phenocryst	46.7	25.7	1.54	98.5	0.27	275	3.19	2.22	2.70	b.d.l	b.d.l	0.12	2.65	0.09	0.57	0.07	0.08
WL-47(102).35	quartz phenocryst	46.7	28.8	1.48	30.1	0.26	211	3.60	2.14	3.13	b.d.l	0.21	0.08	2.70	0.14	0.47	0.14	0.16
WL-47(102).36	quartz phenocryst	46.5	16.0	1.53	1010	0.18	1427	17.2	2.23	2.88	b.d.l	b.d.l	1.17	3.11	0.15	0.65	0.11	0.08
WL-47(102).37	quartz phenocryst	46.7	24.7	1.41	50.8	0.35	208	3.68	2.30	2.75	b.d.l	b.d.l	0.09	3.18	0.10	0.55	0.11	0.10
WL-47(102).38	quartz phenocryst	46.7	24.6	1.55	198	0.13	388	4.44	2.26	2.89	b.d.l	b.d.l	0.27	3.04	0.11	0.56	0.08	0.15
WL-47(102).39	quartz phenocryst	46.6	25.2	1.57	436	0.12	696	7.02	2.17	2.93	b.d.l	0.20	0.51	3.08	0.04	0.69	0.09	0.04
WL-47(102).40	quartz phenocryst	46.7	26.3	1.52	117	0.18	300	6.82	2.18	2.60	0.11	b.d.l	0.18	3.00	0.40	0.41	0.09	0.16
WL-47(102).41	quartz phenocryst	46.7	35.1	2.87	5.12	0.39	203	5.07	2.10	2.51	b.d.l	0.17	0.05	2.61	0.23	0.38	0.13	0.18
WL-47(102).42	quartz phenocryst	46.7	31.3	1.28	44.2	0.31	200	6.08	2.20	2.73	b.d.l	0.22	0.07	2.49	0.22	0.48	0.09	0.15
WL-47(102).43	quartz phenocryst	46.7	25.9	1.40	311	0.35	555	7.91	2.19	3.14	b.d.l	0.29	0.37	3.17	0.21	0.46	0.07	0.19
WL-47(102).44	quartz phenocryst	46.7	28.7	1.35	12.0	0.10	189	3.99	2.15	2.82	b.d.l	b.d.l	0.07	2.97	0.18	0.39	0.08	0.16
WL-47(102).45	quartz phenocryst	46.7	21.9	1.37	19.9	0.17	185	3.84	2.09	2.67	b.d.l	0.17	0.16	2.65	0.12	0.36	0.09	0.11
WL-47(102).46	quartz phenocryst	46.7	25.5	1.11	6.12	0.20	165	3.91	2.10	2.33	b.d.l	b.d.l	0.05	2.57	0.13	0.53	0.05	0.04
WL-47(102).47	quartz phenocryst	46.7	25.0	1.31	32.0	0.28	228	4.59	2.14	3.22	b.d.l	0.28	0.10	3.47	0.15	0.50	0.09	0.09
WL-35.31	vein type	46.7	13.5	5.73	26.6	0.06	74.1	4.91	2.49	4.42	b.d.l	0.48	0.03	3.52	0.24	0.64	b.d.l	0.51
WL-35.32	vein type	46.7	14.1	0.99	1.14	0.07	80.6	b.d.l	2.52	5.91	b.d.l	0.17	0.03	4.08	0.02	0.67	0.12	b.d.l
WL-35.33	vein type	46.7	22.2	1.54	b.d.l	b.d.l	124	b.d.l	2.48	5.95	b.d.l	b.d.l	0.04	4.41	b.d.l	0.59	0.08	b.d.l
WL-35.34	vein type	46.7	7.80	0.61	b.d.l	b.d.l	38.9	b.d.l	2.47	4.72	b.d.l	b.d.l	b.d.l	4.29	b.d.l	0.57	b.d.l	b.d.l
WL-35.35	vein type	46.7	11.4	0.92	1.52	0.23	63.3	b.d.l	2.37	7.16	b.d.l	0.22	0.02	3.92	b.d.l	0.52	0.10	0.01

WL-35.36	vein type	46.7	11.0	0.98	0.88	0.53	59.5	b.d.l	2.62	5.89	b.d.l	b.d.l	0.02	3.87	b.d.l	0.55	0.10	b.d.l
WL-45.48	vein type	46.7	4.40	1.03	1.83	0.14	81.5	2.23	2.25	7.10	b.d.l	0.26	0.03	3.68	b.d.l	0.47	b.d.l	0.01
WL-45.49	vein type	46.7	3.60	0.72	1.80	0.28	72.9	b.d.l	2.31	6.92	b.d.l	b.d.l	0.02	3.81	0.01	0.43	b.d.l	0.01
WL-45.50	vein type	46.7	2.10	1.59	3.73	0.27	75.6	b.d.l	2.06	7.25	0.19	0.48	0.02	3.77	0.04	0.58	0.09	0.08
WL-45.51	vein type	46.7	0.10	0.89	2.34	0.09	62.9	b.d.l	2.14	6.51	b.d.l	0.35	0.04	3.67	b.d.l	0.49	0.04	0.02
WL-45.52	vein type	46.7	0.10	0.95	1.58	0.10	95.4	b.d.l	2.13	9.62	b.d.l	0.44	0.05	4.23	0.02	0.45	0.04	0.02
WL-45.53	vein type	46.7	1.20	0.85	1.75	0.08	84.0	2.04	1.99	7.29	b.d.l	0.31	0.04	3.73	0.01	0.49	0.06	0.02
WL-45.54	vein type	46.7	0.90	7.06	2.57	0.35	67.1	2.31	2.16	7.10	0.31	3.03	0.03	3.19	0.01	0.63	0.14	0.04
WL-45.55	vein type	46.7	0.20	1.37	7.63	0.08	86.7	3.59	2.05	6.69	0.24	b.d.l	0.03	4.00	0.08	0.48	0.07	0.09
WL-45.56	vein type	46.7	1.00	0.95	5.70	0.18	83.3	2.84	2.12	7.57	0.09	b.d.l	0.03	3.59	0.04	0.46	0.06	0.05
WL-45.57	vein type	46.7	5.20	0.94	4.62	0.12	87.2	2.55	2.17	7.86	b.d.l	b.d.l	0.03	3.79	0.02	0.53	0.04	0.04
WL-45.58	vein type	46.7	0.20	7.99	29.4	0.35	47.2	7.38	2.18	6.37	0.55	1.61	0.02	3.74	0.12	0.44	0.64	0.36
WL-45.59	vein type	46.7	0.30	3.38	26.3	0.13	96.8	5.12	2.17	7.85	1.55	0.99	0.02	3.62	0.11	0.73	0.33	0.20
WL-45.60	vein type	46.7	0.20	0.82	8.66	0.22	104	4.09	2.05	8.41	b.d.l	0.37	0.03	4.24	0.03	0.44	0.14	0.06
WL-45.61	vein type	46.7	0.10	0.86	0.96	0.10	39.1	b.d.l	2.12	5.38	0.08	0.31	b.d.l	3.62	0.02	0.52	b.d.l	0.05
WL-45.62	vein type	46.7	b.d.l	0.73	b.d.l	0.18	45.7	1.61	2.03	6.40	b.d.l	0.20	b.d.l	4.28	0.01	0.62	0.04	b.d.l
NW-Y-127(39).63	breccia pipe	46.7	19.0	1.21	1.81	0.10	102	1.72	2.01	8.44	b.d.l	b.d.l	0.03	4.57	0.04	0.40	0.05	0.02
NW-Y-127(39).64	breccia pipe	46.7	19.7	0.70	3.66	0.09	113	3.70	2.02	9.23	b.d.l	b.d.l	0.03	4.54	0.08	0.42	b.d.l	0.01
NW-Y-127(39).65	breccia pipe	46.7	18.8	1.00	1.42	0.11	96.5	b.d.l	2.07	8.00	b.d.l	b.d.l	0.03	3.82	0.02	0.62	b.d.l	0.01
NW-Y-127(39).66	breccia pipe	46.7	20.8	1.10	1.22	0.08	106	2.82	2.18	8.96	0.10	b.d.l	0.04	4.41	0.02	0.48	b.d.l	0.01
NW-Y-127(39).67	breccia pipe	46.7	22.0	1.18	1.86	0.09	117	2.77	2.11	9.52	b.d.l	b.d.l	0.04	4.82	0.11	0.45	b.d.l	0.03
NW-Y-127(39).68	breccia pipe	46.7	18.2	1.01	1.98	0.06	93.3	1.79	2.04	9.05	b.d.l	b.d.l	0.03	4.45	0.04	0.51	b.d.l	0.03
NW-Y-127(39).69	breccia pipe	46.7	18.3	1.31	1.57	0.10	99.2	b.d.l	2.16	8.57	b.d.l	b.d.l	0.04	4.22	0.01	0.48	0.03	0.02
NW-Y-127(39).70	breccia pipe	46.7	18.6	0.93	b.d.l	0.16	93.1	2.60	2.06	9.07	b.d.l	b.d.l	0.02	4.56	0.02	0.51	b.d.l	0.01
NW-Y-127(39).71	breccia pipe	46.7	22.6	1.12	2.95	0.19	123	3.12	2.04	13.1	b.d.l	b.d.l	0.03	5.17	0.10	0.57	b.d.l	0.01
NW-Y-127(39).72	breccia pipe	46.7	20.3	1.18	5.92	0.12	114	4.02	2.00	12.2	b.d.l	0.23	0.03	4.59	0.11	0.56	0.09	0.07
NW-Y-127(39).73	breccia pipe	46.7	29.4	1.16	2.92	0.06	219	3.07	2.08	17.8	b.d.l	0.21	0.06	6.17	0.32	0.57	0.04	0.13
NW-Y-127(39).74	breccia pipe	46.7	20.2	0.89	1.08	0.12	129	b.d.l	2.07	11.4	b.d.l	b.d.l	0.05	4.07	0.02	0.41	b.d.l	0.01
NW-Y-127(39).75	breccia pipe	46.7	20.9	1.15	3.43	0.13	121	4.25	2.15	11.7	b.d.l	b.d.l	0.04	4.39	0.19	0.41	b.d.l	0.08
NW-Y-127(39).76	breccia pipe	46.7	23.6	1.08	4.31	0.11	133	2.22	2.15	10.8	b.d.l	0.99	0.03	5.06	0.07	0.49	0.04	0.07
NW-Y-127(39).77	breccia pipe	46.7	17.7	0.89	1.52	0.06	105	2.67	2.07	9.61	b.d.l	b.d.l	0.03	4.84	0.09	0.53	b.d.l	0.10

Measured concentrations in quartz crystal (sample WQ-5) from a vug

Sample	Li ppm	B ppm	Al ppm	Sc ppm	Ti ppm	Ge ppm	Sn ppm	Ge/Ti ppm/ppm	Al/Ti ppm/ppm
Ep 2Rim-1-1	2.42	b.d.l	14.7	4.31	4.30	1.57	2.46	0.37	3.41
Ep 2Rim-1-2	18.6	3.61	101	4.86	5.14	1.23	2.70	0.24	19.8
Ep 2Rim-1-3	15.1	2.78	91.4	4.43	4.66	1.25	3.06	0.27	19.6
Ep 2Rim-1-4	12.3	0.01	63.1	4.77	3.09	2.20	0.44	0.71	20.4
Ep 2Rim-1-5	6.03	b.d.l	27.2	4.90	5.18	2.24	2.07	0.43	5.25
Ep 2Rim-1-6	8.96	2.04	44.7	4.32	5.04	1.78	2.01	0.35	8.87
Ep 2Rim-1-7	7.71	b.d.l	39.4	4.35	4.92	1.54	2.35	0.31	8.01
Ep 2Rim-1-8	8.59	0.98	46.5	4.80	5.31	2.09	2.80	0.39	8.75
Ep 2Rim-1-9	9.62	2.31	48.5	4.57	5.10	2.10	1.07	0.41	9.52
Ep 2Rim-1-10	10.5	b.d.l	52.0	4.10	5.85	3.53	1.84	0.60	8.88
Ep 2Rim-1-11	8.40	b.d.l	42.1	4.51	4.75	2.65	2.01	0.56	8.86
Ep 2Rim-1-12	9.90	b.d.l	54.2	3.69	3.77	2.64	2.58	0.70	14.4
Ep 2Rim-1-13	8.81	0.29	43.9	3.67	6.29	4.32	2.43	0.69	6.98
Ep 2Rim-1-14	5.95	b.d.l	30.6	3.79	3.62	3.75	2.65	1.04	8.46
Ep 2Rim-1-15	4.51	b.d.l	31.0	2.85	5.41	2.77	1.19	0.51	5.73
Ep 2Rim-1-16	7.63	11.6	46.9	4.54	4.96	2.74	1.94	0.55	9.46
Ep 2Rim-1-17	8.69	3.89	43.5	3.29	4.86	1.91	1.10	0.39	8.94
Ep 2Rim-1-18	3.53	0.42	17.9	2.73	2.58	1.42	0.95	0.55	6.95
Ep 2Rim-1-19	3.20	0.40	17.1	2.54	2.43	1.37	0.86	0.56	7.06
Ep 2Rim-1-20	10.4	0.42	52.7	2.38	3.32	1.63	0.90	0.49	15.9
Ep 2Rim-1-21	3.67	0.54	17.7	2.27	2.58	1.76	0.59	0.68	6.87
AVERAGE	8.31	1.40	44.1	3.89	4.44	2.21	1.81	0.52	10.1
Ep 2Core-1-1	20.0	b.d.l	103	4.38	5.92	2.58	1.43	0.44	17.4
Ep 2Core-1-2	14.2	3.39	68.3	3.93	5.25	3.22	1.33	0.61	13.0
Ep 2Core-1-3	15.3	2.64	73.7	4.03	6.26	2.31	2.31	0.37	11.8
Ep 2Core-1-4	23.9	2.52	123	4.10	5.83	3.35	1.38	0.57	21.0
Ep 2Core-1-5	4.13	0.69	19.6	4.06	2.76	1.60	2.08	0.58	7.10
Ep 2Core-1-6	23.0	0.30	127	4.12	5.25	1.87	1.47	0.36	24.2
Ep 2Core-1-7	13.7	2.21	69.4	3.84	5.23	1.99	1.64	0.38	13.3
Ep 2Core-1-8	20.1	1.17	104	3.65	5.09	2.37	1.08	0.47	20.4
Ep 2Core-1-9	25.0	0.99	122	3.51	5.67	2.51	1.32	0.44	21.6
Ep 2Core-1-10	8.60	3.71	38.3	3.72	4.54	2.32	1.90	0.51	8.44
Ep 2Core-1-11	13.9	0.30	69.9	3.64	5.23	2.32	1.66	0.44	13.4
Ep 2Core-1-12	10.3	3.41	46.9	3.35	4.87	2.41	1.25	0.49	9.64
Ep 2Core-1-13	23.1	5.64	119	2.94	4.99	2.40	0.95	0.48	23.8
Ep 2Core-1-14	16.1	4.67	75.6	3.29	5.58	2.29	1.63	0.41	13.6
Ep 2Core-1-15	15.2	b.d.l	92.8	3.13	4.88	1.95	1.39	0.40	19.0
Ep 2Core-1-16	15.6	0.93	79.4	3.24	4.46	1.44	0.88	0.32	17.8
Ep 2Core-1-17	11.2	2.04	56.3	3.19	3.79	2.40	1.03	0.63	14.9
Ep 2Core-1-18	16.5	0.88	84.8	2.37	3.33	1.87	0.69	0.56	25.4
Ep 2Core-1-19	16.9	1.07	87.3	2.32	4.93	1.63	0.51	0.33	17.7
Ep 2Core-1-20	8.94	0.57	41.9	2.34	4.81	2.73	0.63	0.57	8.72
Ep 2Core-1-21	6.44	0.71	31.9	2.35	4.89	2.16	0.52	0.44	6.51
AVERAGE	15.3	1.80	77.8	3.41	4.93	2.27	1.29	0.47	15.7
Ep 1Rim-1-1	5.57	b.d.l	21.9	3.59	3.34	2.15	1.54	0.64	6.55
Ep 1Rim-1-2	5.14	2.80	25.4	3.19	3.48	1.72	1.83	0.49	7.31
Ep 1Rim-1-3	5.27	0.10	23.7	3.31	3.62	2.64	1.49	0.73	6.53

Ep 1Rim-1-4	4.83	1.66	19.7	3.12	4.10	1.97	1.44	0.48	4.82
Ep 1Rim-1-5	6.60	b.d.l	32.8	3.43	4.66	1.96	1.56	0.42	7.04
Ep 1Rim-1-6	8.48	1.85	43.8	3.48	4.65	0.90	1.70	0.19	9.42
Ep 1Rim-1-7	6.27	3.72	30.8	2.91	3.54	3.40	1.08	0.96	8.70
Ep 1Rim-1-8	3.65	6.44	25.1	2.75	2.90	4.05	3.24	1.39	8.66
Ep 1Rim-1-9	2.89	b.d.l	17.8	2.93	3.89	2.38	1.71	0.61	4.58
Ep 1Rim-1-10	4.28	2.38	22.1	2.40	5.20	4.28	1.26	0.82	4.25
Ep 1Rim-1-11	17.8	1.80	86.6	3.33	5.56	2.64	1.40	0.47	15.6
Ep 1Rim-1-12	15.1	0.56	66.4	3.48	4.28	2.18	1.50	0.51	15.5
Ep 1Rim-1-13	17.6	0.83	83.4	3.24	5.32	2.47	2.07	0.46	15.7
Ep 1Rim-1-14	17.6	2.11	89.6	3.20	6.24	2.54	1.16	0.41	14.4
Ep 1Rim-1-15	16.3	1.49	80.9	3.40	4.76	2.34	1.52	0.49	17.0
Ep 1Rim-1-16	17.4	b.d.l	75.4	3.53	5.33	2.35	1.87	0.44	14.1
Ep 1Rim-1-17	11.2	5.92	38.8	3.39	5.95	3.30	0.07	0.55	6.52
Ep 1Rim-1-18	7.46	0.47	43.4	2.30	5.30	2.49	0.52	0.47	8.19
Ep 1Rim-1-19	4.31	0.65	21.5	2.37	3.62	2.04	0.61	0.56	5.95
Ep 1Rim-1-20	8.81	0.71	42.4	2.21	4.14	2.60	0.55	0.63	10.2
Ep 1Rim-1-21	5.04	0.61	29.6	2.33	2.42	2.97	0.63	1.23	12.2
AVERAGE	9.12	1.62	43.9	3.04	4.40	2.54	1.37	0.62	9.68
Ep 1Core-1-1	25.7	2.30	114	3.11	7.44	3.57	1.12	0.48	15.3
Ep 1Core-1-2	25.8	5.69	124	2.44	6.95	3.26	1.18	0.47	17.8
Ep 1Core-1-3	13.5	7.98	48.8	3.05	6.65	2.36	2.81	0.35	7.34
Ep 1Core-1-4	49.1	9.12	264	2.83	6.90	5.09	0.82	0.74	38.3
Ep 1Core-1-5	19.8	11.7	149	2.35	2.66	5.49	0.67	2.07	56.0
Ep 1Core-1-6	15.4	3.37	92.4	2.53	3.80	3.92	0.38	1.03	24.3
Ep 1Core-1-7	43.1	8.97	248	2.90	7.34	3.16	0.77	0.43	33.8
Ep 1Core-1-8	43.6	b.d.l	249	2.52	3.33	4.01	0.59	1.20	74.8
Ep 1Core-1-9	25.8	2.99	165	2.37	2.82	2.85	0.79	1.01	58.5
Ep 1Core-1-10	28.8	1.42	185	2.68	3.71	3.62	0.61	0.98	49.9
Ep 1Core-1-11	31.1	5.91	197	2.50	4.38	3.41	0.79	0.78	45.0
Ep 1Core-1-12	30.5	7.22	193	2.63	5.45	2.62	0.71	0.48	35.4
Ep 1Core-1-13	33.2	3.40	206	2.73	5.15	2.86	1.14	0.56	40.0
Ep 1Core-1-14	33.8	3.56	205	2.66	5.61	3.35	1.15	0.60	36.6
Ep 1Core-1-15	34.2	3.93	202	2.89	3.58	3.65	0.83	1.02	56.3
Ep 1Core-1-16	36.2	2.94	215	2.77	5.13	3.10	0.99	0.60	41.9
Ep 1Core-1-17	36.2	0.20	221	2.74	5.34	3.93	1.07	0.74	41.4
Ep 1Core-1-18	35.8	4.69	202	2.82	6.58	3.17	1.23	0.48	30.7
Ep 1Core-1-19	33.6	1.36	197	3.03	5.73	2.92	0.98	0.51	34.4
Ep 1Core-1-20	5.13	0.86	24.7	2.22	4.09	2.70	0.65	0.66	6.03
Ep 1Core-1-21	9.49	0.75	48.2	2.26	4.03	2.53	0.62	0.63	12.0
Ep 1Core-1-22	9.30	0.81	46.4	2.22	4.60	2.55	0.51	0.55	10.1
Ep 1Core-1-23	6.65	0.61	31.5	2.17	4.12	2.63	0.61	0.64	7.65
AVERAGE	27.2	3.90	158	2.63	5.02	3.34	0.91	0.74	33.6
